# Supplementary material for: Do mobile device apps designed to support medication adherence demonstrate efficacy? A systematic review of randomised controlled trials, with meta-analysis
Source: BMJ Open. 2020 Jan 30;10(1):e032045. doi: 10.1136/bmjopen-2019-032045 (PMC7045248; doi:10.1136/bmjopen-2019-032045)
Supplement: Supplementary data [file bmjopen-2019-032045supp001.pdf]

SUPPLEMENTAL MATERIAL FILE

Mobile device apps to support medication adherence:

A systematic review of randomised controlled trials,

with meta-analysis

Author Details:

Laura Armitage, MRCGP

Aikaterini Kassavou, PhD

Stephen Sutton, PhD

Armitage LC, *et al. BMJ Open* 2020; 10:e032045. doi: 10.1136/bmjopen-2019-032045

eText 1: Medline/Pubmed Search Strategy

1. medication adherence.mp. or exp Patient Compliance/ or exp Medication Adherence/
2. nonnadherence.mp.
3. non-adherence.mp.
4. non adherence.mp.
5. concordance.mp.
6. concordant.mp.
7. 1 or 2 or 3 or 4 or 5 or 6
8. app.mp.
9. apps.mp.
10. application.mp.
11. exp Mobile Applications/ or mobile application*.mp.
12. smartphone.mp. or exp Smartphone/ or exp Cell Phones/
13. mobile*.mp.
14. phone*.mp.
15. 8 or 9 or 10 or 11 or 12 or 13 or 14
16. randomized controlled trial.mp. or exp Randomized Controlled Trial/
17. RCT.mp.
18. randomised.mp.
19. randomized.mp.
20. control*.mp.
21. 16 or 17 or 18 or 19 or 20
22. 7 and 15 and 21
23. limit 22 to (english language and yr="1990 - 2017")

Armitage LC, *et al. BMJ Open* 2020; 10:e032045. doi: 10.1136/bmjopen-2019-032045

eFigure 1: Funnel Plot of effect estimates from studies included in meta-analysis

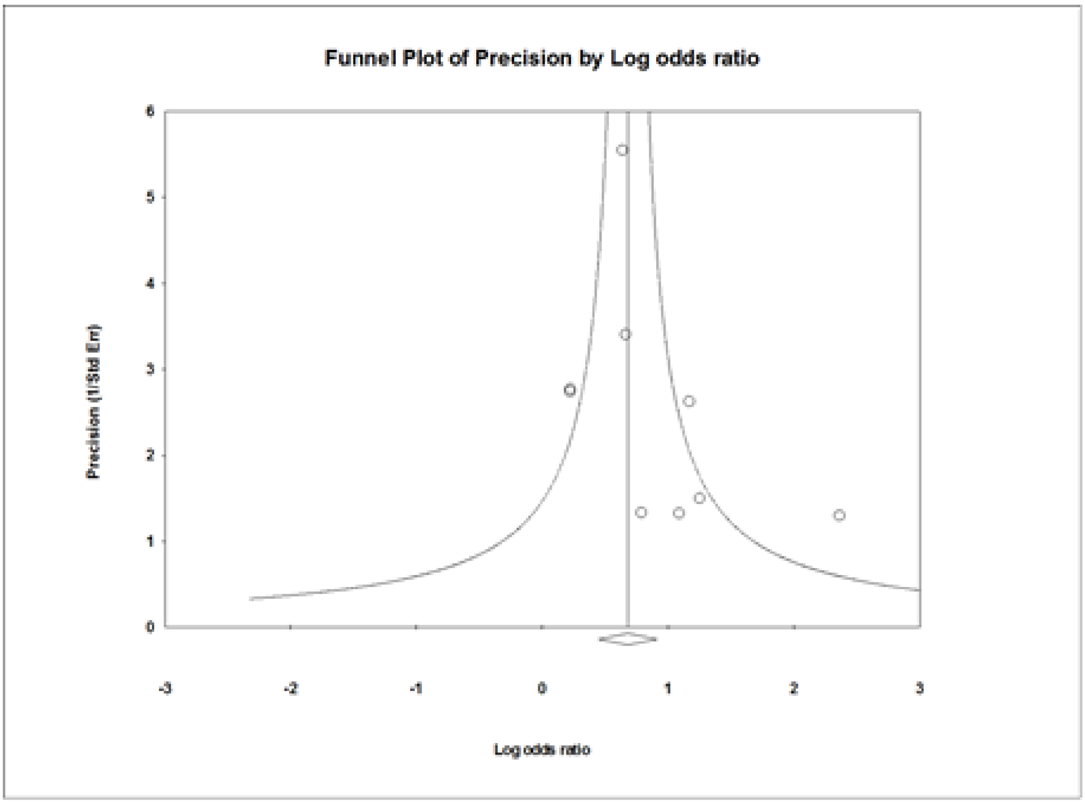

Armitage LC, *et al. BMJ Open* 2020; 10:e032045. doi: 10.1136/bmjopen-2019-032045

eTable 1: Relative weighting of each study included in the 9 study meta-analysis

Study	Weight (random)	Relative weight
Hammonds et al, 2015	2.16	3.79
Labovitz et al, 2017	1.63	2.86
Lakshminarayana et al, 2017	9.76	17.10
Mertens et al, 2018	1.70	2.97
Mira et al, 2014	6.69	11.72
Morawski et al, 2018	20.58	36.03
Santo et al, 2018	6.68	11.69
Shah et al, 2016	1.72	3.01
Svendsen et al, 2018	6.19	10.84

Armitage LC, *et al. BMJ Open* 2020; 10:e032045. doi: 10.1136/bmjopen-2019-032045

eTable 2: Raw data extracted for each study for meta-analysis

Study	Adherence measurement	Intervention group				Comparator group			
		Mean (SD)	N	Events	Totals	Mean (SD)	N	Events	Totals
Hammonds et al, 2015	Number of participants taken ≥80% prescribed tablets per day	-	-	12	20	-	-	6	20
Labovitz et al, 2017	Mean cumulative adherence, measured by pill count	97.2 (4.4)	15	-	-	90.6 (5.8)	12	-	-
Lakshminaray-ana et al, 2017	MMAS-8	6.3 (1.52)	68	-	-	5.74 (1.53)	90	-	-
Mertens et al, 2018	A14 Scale	53.96 (2.01)	12	-	-	52.6 (2.49)	12	-	-
Mira et al, 2014	MMAS-4	7.4 (0.9)	51	-	-	7.3 (0.7)	48	-	-
Morawski et al, 2018	MMAS-8	6.3 (1.6)	209	-	-	5.7 (1.8)	202	-	-
Santo et al, 2018	MMAS-8	7.02 (n/a)	50	-	-	6.63 (n/a)	51	-	-
Shah et al, 2016	MMAS-8	2.2 (1.2)	5	-	-	2.1 (1)	3	-	-
Svendsen et al, 2018	Number of patients having applied medication ≥80% days in treatment period	-	-	39	59	-	-	23	61

A14 = A14 Medication Adherence Scale; MMAS-8 = 8 Item Morisky Medication Adherence Scale; MMAS 4 = 4 Item Morisky Medication Adherence Scale

Armitage LC, *et al. BMJ Open* 2020; 10:e032045. doi: 10.1136/bmjopen-2019-032045

eTable 3: Behaviour Change Techniques utilised by in apps in the included studies.

Study	BCTs Coded							
	Tailored	Prompts and cues	Report whether behaviour was performed	Monitoring of behaviour by others without feedback	Feedback on behaviour	Social support (unspecified)	Habit formation	Goal setting of behaviour
Hammonds et al, 2015	+	+	+	-	-	-	-	-
Labovitz et al, 2017	+	+	+	+	+	-	-	-
Lakshminarayana et al, 2017	+	+	+	+	+	-	-	-
Mertens et al, 2016	+	+	+	-	-	-	-	-
Mira et al, 2014	+	+	+	+	+	-	+	+
Morawski et al, 2018	+	-	+	-	+	+	+	+
Santo et al, 2018	+	-	+	-	+	+	+	-
Shah et al, 2016	+	-	-	-	-	+	-	-
Svendsen et al, 2018	+	-	-	-	+	-	-	-

Those Behaviour Change Techniques coded in 3 but not more than 6 studies were included for meta-regression.

eTable 4: Definitions of Behaviour Change Techniques coded as present in apps of the included studies and examples of each.

Behaviour Change Technique	Definition	Examples
Tailored	Use information gained about the person to define the content, frequency or format of an intervention directed to the behaviour.	Shah et al, 2016: information about the participant including their symptoms and coronary artery catheterising procedure were recorded and used to tailor the information delivered to the participant via the MyIDEA application.[1]<br><br>Mira et al, 2014: The ALICE app delivered a customised system of alerts and reminders, according to their personalised prescription to remind participants when to take their medication.[2]
Prompts and Cues	"Introduce or define environmental or social stimulus with the purpose of prompting or cueing the behaviour. The prompt and cue would normally occur at the time or place of performance".[3]	Mira et al, 2014: The ALICE app delivered a customised system of alerts and reminders, according to their personalised prescription to remind participants when to take their medication.[2]<br><br>Lakshminarayana et al, 2017: The app included a reminder system for participants to set up and allow medication alerts.[4]
Report whether or not behaviour was performed	Ask a person to report when they have performed a behaviour without the requirement of monitoring or recording the behaviour.[5]	Santo et al, 2018: Reminders from the application could be snoozed, rescheduled and/or marked as 'taken' or 'missed dose'.[6]<br><br>Hammonds et al, 2015: Participants indicated when they had taken their medication by responding to the alert message.[7]

Armitage LC, *et al. BMJ Open* 2020; 10:e032045. doi: 10.1136/bmjopen-2019-032045

Monitoring of behaviour by others without feedback	"Observe or record behaviour with the person's knowledge as part of a behaviour change strategy".[3]	Labovitz et al, 2017: Clinic staff received automated text messages or emails if doses were missed, late or based on incorrect usage.[8]<br><br>Lakshminarayana et al, 2017: Clinicians had access to a dedicated web portal where they could see the participants' progress over the trial period.[9]
Feedback on behaviour	"Monitor and provide informative or evaluative feedback on performance of the behaviour (e.g. form, frequency, duration, intensity".[3]	Mira et al, 2014: The ALICE app reported to the participant medication that they had taken that day and those that the participant had not taken.[2]<br><br>Santo et al, 2018: The app included a feature of medication adherence statistics.[10]
Social support (unspecified)	"Advise on, arrange or provide social support (e.g. from friends, relatives, colleagues, buddies or staff) or non-contingent praise or reward for performance of the behaviour. It includes encouragement and counselling, but only when it is directed at the behaviour".[3]	Morawski et al, 2018: Users were able to designate a "Medfriend" who was granted access to the person's medication taking history and received alerts when doses were missed and could provide peer support.[11]<br><br>Santo et al, 2018: The app included the ability to export and share information with others such as family members and the ability to alert them if the participant missed a dose.[10]
Habit formation	"Prompt rehearsal and repetition of the behaviour in the same context repeatedly so that the context elicits the behaviour".[3]	Mira et al 2014: alerts and reminders encouraged participants to put healthy habits into practice, e.g. the association of taking their medicines with particular meals or daily activities.[2]<br><br>Santo et al, 2018: Scheduled reminders occurred up to three times at 10 minute intervals.[10]

Armitage LC, *et al. BMJ Open* 2020; 10:e032045. doi: 10.1136/bmjopen-2019-032045

Goal setting of behaviour	"Set or agree a goal defined in terms of the behaviour to be achieved".[3]	Mira et al, 2014: The ALICE app included a function for the participant to store details of their prescriptions and related instructions.[2]<br><br>Morawski et al, 2018: Users were able to enter their medication lists manually, along with their preferred timing of administration.[11]

References

1 Shah V, Dileep A, Dickens C, *et al.* Patient-Centered Tablet Application for Improving Medication Adherence after a Drug-Eluting Stent. *Front public Heal* 2016;**4**:272. doi:10.3389/fpubh.2016.00272

2 Mira JJ, Navarro I, Botella F, *et al.* A Spanish pillbox app for elderly patients taking multiple medications: randomized controlled trial. *J Med Internet Res* 2014;**16**:e99. doi:10.2196/jmir.3269

3 Michie S, Richardson M, Johnston M, *et al.* The Behavior Change Technique Taxonomy (v1) of 93 Hierarchically Clustered Techniques: Building an International Consensus for the Reporting of Behavior Change Interventions. *Ann Behav Med* 2013;**46**:81–95. doi:10.1007/s12160-013-9486-6

4 Lakshminarayana R, Wang D, Burn D, *et al.* Using a smartphone-based self-management platform to support medication adherence and clinical consultation in Parkinson ' s disease. *NPJ Park Dis* 2017;**3**:1–8. doi:10.1038/s41531-016-0003-z

5 Kassavou A, Sutton S. Automated telecommunication interventions to promote adherence to cardio-metabolic medications: meta-analysis of effectiveness and meta-regression of behaviour change techniques. *Health Psychol Rev* 2018;**12**:25–42. doi:10.1080/17437199.2017.1365617

6 Santo K, Singleton A, Rogers K, *et al.* Medication reminder applications to improve adherence in coronary heart disease: a randomised clinical trial. *Heart* 2018;:heartjnl-2018-313479. doi:10.1136/heartjnl-2018-313479

7 Hammonds T, Rickert K, Goldstein C, *et al.* Adherence to Antidepressant Medications: A Randomized Controlled Trial of Medication Reminding in

College Students. 2015;**8481**. doi:10.1080/07448481.2014.975716

8 Labovitz DL, Shafner L, Gil MR, *et al.* Using Artificial Intelligence to Reduce the Risk of Nonadherence in Patients on Anticoagulation Therapy. *Stroke* 2017;**48**:1416–9. doi:10.1161/STROKEAHA.116.016281

9 Lakshminarayana R, Wang D, Burn D, *et al.* and adherence tools to manage Parkinson ' s disease (SMART-PD): study protocol for a randomised controlled trial (v7 ; 15 August 2014). 2014;:1–11.

10 Santo K, Chow CK, Thiagalingam A, *et al.* MEDication reminder APPs to improve medication adherence in Coronary Heart Disease (MedApp-CHD) Study : a randomised controlled trial protocol. 2017;:1–10. doi:10.1136/bmjopen-2017-017540

11 Morawski K, Ghazinouri R, Krumme A, *et al.* Association of a Smartphone Application With Medication Adherence and Blood Pressure Control. *JAMA Intern Med* 2018;**178**:802. doi:10.1001/jamainternmed.2018.0447
