# Supplementary material for: Do mobile device apps designed to support medication adherence demonstrate efficacy? A systematic review of randomised controlled trials, with meta-analysis
Source: BMJ Open. 2020 Jan 30;10(1):e032045. doi: 10.1136/bmjopen-2019-032045 (PMC7045248; doi:10.1136/bmjopen-2019-032045)
Supplement: Author's manuscript [file bmjopen-2019-032045.draft_revisions.pdf]

BMJ Open

BMJ Open is committed to open peer review. As part of this commitment we make the peer review history of every article we publish publicly available.

When an article is published we post the peer reviewers' comments and the authors' responses online. We also post the versions of the paper that were used during peer review. These are the versions that the peer review comments apply to.

The versions of the paper that follow are the versions that were submitted during the peer review process. They are not the versions of record or the final published versions. They should not be cited or distributed as the published version of this manuscript.

BMJ Open is an open access journal and the full, final, typeset and author-corrected version of record of the manuscript is available on our site with no access controls, subscription charges or pay-per-view fees (http://bmjopen.bmj.com).

If you have any questions on BMJ Open's open peer review process please email info.bmjopen@bmj.com

BMJ Open

Are mobile device applications designed to support medication adherence effective? A systematic review of randomised controlled trials, with meta-analysis

Journal:	*BMJ Open*
Manuscript ID	bmjopen-2019-032045
Article Type:	Research
Date Submitted by the Author:	30-May-2019
Complete List of Authors:	Armitage, Laura; University of Oxford, Nuffield Department of Primary Care Health Sciences;<br>Kassavou, Aikaterini; University of Cambridge, Public Health and Primary Care ; University of Cambridge,<br>Sutton, Stephen; University of Cambridge,
Keywords:	Telemedicine < BIOTECHNOLOGY & BIOINFORMATICS, PRIMARY CARE, PUBLIC HEALTH, World Wide Web technology < BIOTECHNOLOGY & BIOINFORMATICS

SCHOLARONE™
Manuscripts

I, the Submitting Author has the right to grant and does grant on behalf of all authors of the Work (as defined in the below author licence), an exclusive licence and/or a non-exclusive licence for contributions from authors who are: i) UK Crown employees; ii) where BMJ has agreed a CC-BY licence shall apply, and/or iii) in accordance with the terms applicable for US Federal Government officers or employees acting as part of their official duties; on a worldwide, perpetual, irrevocable, royalty-free basis to BMJ Publishing Group Ltd ("BMJ") its licensees and where the relevant Journal is co-owned by BMJ to the co-owners of the Journal, to publish the Work in this journal and any other BMJ products and to exploit all rights, as set out in our licence.

The Submitting Author accepts and understands that any supply made under these terms is made by BMJ to the Submitting Author unless you are acting as an employee on behalf of your employer or a postgraduate student of an affiliated institution which is paying any applicable article publishing charge ("APC") for Open Access articles. Where the Submitting Author wishes to make the Work available on an Open Access basis (and intends to pay the relevant APC), the terms of reuse of such Open Access shall be governed by a Creative Commons licence – details of these licences and which Creative Commons licence will apply to this Work are set out in our licence referred to above.

Other than as permitted in any relevant BMJ Author's Self Archiving Policies, I confirm this Work has not been accepted for publication elsewhere, is not being considered for publication elsewhere and does not duplicate material already published. I confirm all authors consent to publication of this Work and authorise the granting of this licence.

For peer review only

Title

Are mobile device applications designed to support medication adherence effective? A systematic review of randomised controlled trials, with meta-analysis

Word Count of Manuscript Text: 3108

Author Details:

Laura Armitage, MRCGP[1,2,3]

Aikaterini Kassavou, PhD[1]

Stephen Sutton, PhD[1]

Affiliations:

1. Department of Public Health and Primary Care, University of Cambridge, UK
2. National Institute for Health Research, Collaboration for Leadership in Applied Health Research and Care, East of England, UK
3. Nuffield Department of Primary Care Health Sciences, University of Oxford, UK

Author for Correspondence:

Laura Armitage

Email: laura.armitage@phc.ox.ac.uk Tel: +447791366355

Nuffield Department of Primary Care Health Sciences,

Radcliffe Primary Care Building, Radcliffe Observatory Quarter,

Woodstock Road,

Oxford,

OX2 6GG

For peer review only
For peer review only - http://bmjopen.bmj.com/site/about/guidelines.xhtml

ABSTRACT

Objectives

To estimate the effectiveness of app-based interventions designed to support medication adherence and establish which behaviour change techniques utilised by applications are associated with an adherence effect.

Design and Methods

We conducted a systematic review of randomised controlled trials where the intervention group received a mobile application to support medication adherence and the comparator group received usual care. Medline/PubMed, PsycINFO, CINAHL, Embase and Web of Science databases were searched from 1990 to November 2018. Reference searches of included articles were conducted.

Data from included studies were extracted independently by two reviewers. Authors were contacted by email for data which was not available in published articles.

Main Outcome and Measure

The main outcome was the pooled effect size of changes in medication adherence. We also sought to perform meta-regression of the behaviour change techniques utilised by the applications, to investigate any association with effect size.

Results

The initial search identified 13 295 citations. After title and abstract screening, the full text articles of 83 studies were screened for eligibility. Nine studies, reporting data on 1005 participants were eligible and included in the analysis. Meta-analysis indicated that patients who use mobile applications to support medication taking are more likely to adhere to medications (OR 2.120, 95% CI 1.635-2.747) in comparison to usual care. I^2 estimate of heterogeneity was low, at 9.8. Meta-

For peer review only - http://bmjopen.bmj.com/site/about/guidelines.xhtml

For peer review only

regression of the behaviour change techniques did not reveal any significant associations with effect size.

Conclusions

App-based medication adherence interventions have a significant positive effect on patients' adherence. Larger scale studies are required to further evaluate this effect and explore intervention characteristics associated with the most effective apps. As mobile devices reach ubiquity, the routine recommendation of medication adherence apps may serve to improve adherence at a population level and help patients achieve full benefit of prescribed medications.

Article Summary – Strengths and Limitations of this Study

- To our knowledge, this is the first non-disease specific systematic review of randomised controlled trials investigating mobile device applications to support medication adherence.
- This review is reported according to the PRISMA Statement.
- A sensitive search strategy was designed and screening of articles and data extraction was performed independently by one clinical researcher and one non-clinical researcher.
- This review cannot report on the likely sustainability of the effect of mobile applications on medication adherence, given the short study duration of the studies included in this review.

For peer review only - http://bmjopen.bmj.com/site/about/guidelines.xhtml

For peer review only

MANUSCRIPT TEXT

Introduction

Adherence to medication is defined as the extent to which patients take medications as prescribed and agreed with their health care providers.[1] The therapeutic benefit of prescribed medications is often limited owing to an estimated 50% of patients not adhering to medications.[2] The annual cost of non-adherence is estimated to be in excess of £930 million in England[3] and between $100 and $300 billion in the US.[4] Furthermore, in 2003 the Adherence to Long-term Therapy report published by the World Health Organisation stated that unless medication adherence is addressed, advances in biomedical technology will not achieve their potential.[5]

Interventions for improving adherence should be reliable, contemporary, acceptable and readily available to the person. Mobile devices can meet these needs and in 2018, Ofcom reported that 78% of adults and 95% of 16-24 year olds in the UK own a smartphone, demonstrating that smartphones are a growing part of modern life.[6] Patients who are prescribed multiple medications are commonly in the older age group[7] where the percentage of smartphone users may be lower.[8] However, smartphone ownership in those aged over 65 more than trebled between the years 2012 and 2015 from 5% to 18%, and is set to continue to increase.[9]

Hundreds of applications are now available to patients to support them in taking regular medication.[10] Such applications frequently utilise behaviour change techniques (BCTs) to promote improvements in adherence. A behaviour change technique is defined as an active component of an intervention that is designed to change behaviour.[11] To date, the effectiveness of smartphone apps to support patient adherence to regular prescribed medications has not been evaluated within the scientific literature using rigorous quantitative review. Consequently, we have performed a novel systematic review with meta-analysis, to evaluate the effectiveness of smartphone applications in supporting medication adherence.

For peer review only - http://bmjopen.bmj.com/site/about/guidelines.xhtml

The objectives of this review are (i) to establish whether applications designed to support medication adherence are effective and (ii) to identify the intervention characteristics and Behavioural Change Techniques (BCTs) associated with effective applications. The conclusions drawn have potential to inform future large-scale public health interventions for the improvement of medication adherence.

METHODS

This systematic review is reported according to the PRISMA statement.[12] The protocol for the review is registered with and published on the PROSPERO database of systematic reviews (registration number: CRD42017080150).[13] The scope of the review, inclusion and exclusion criteria remained unchanged throughout.

Patient and Public Involvement

This research was done without patient involvement. Patients were not invited to comment on the study design and were not consulted to develop patient relevant outcomes or interpret the results. Patients were not invited to contribute to the writing or editing of this document for readability or accuracy.

Data Sources

Medline/PubMed, PsycINFO, CINAHL, Embase and Web of Science electronic databases were searched from 1990 to November 2018. A sensitive search strategy was designed based on preliminary searches, relevant papers and key words. Key search terms included adherence, non-adherence, smart phone application and randomised control trials (RCTs). An example detailed search strategy for the Medline/PubMed database can be found in supplementary information, (eText 1). Reference lists of included studies were screened for additional relevant trials.

For peer review only - http://bmjopen.bmj.com/site/about/guidelines.xhtml

Two reviewers (LA and AK) independently screened all citations by title and abstract and excluded those which were irrelevant or clearly did not meet eligibility criteria. Full text articles were obtained for all remaining citations and screened against the eligibility criteria by the same reviewers. Any discrepancies at any stage in the screening process were settled through discussion between LA, AK and with a third reviewer (SS).

Study Selection

RCTs relevant to this review were those which investigated the use of an application (app) on a mobile device to support medication adherence. Eligibility criteria were as follows:

(i) participants of any age who were taking one or more medications prescribed for treatment of a medical condition

(ii) an intervention group which received an app-based intervention to help, support or advise about medication adherence

(iii) a comparator group which received usual care, another medication adherence intervention which did not utilise an app,[14] or an app which did not include any behaviour change techniques (for example an application that was a non-health related game[15]).

Studies were eligible for meta-analysis if they measured medication adherence for the intervention and comparator groups.[14–19] Studies were excluded from the meta-analysis if the outcome assessed was adherence to a number of treatment components out with prescribed medication. For example one study reported outcome data as a composite score, combining adherence to other aspects of the medical regimen (such as clinic attendance and laboratory work) with adherence to medication.[20] Published articles were required to be in English language.

Data Extraction and synthesis

For peer review only - http://bmjopen.bmj.com/site/about/guidelines.xhtml

Data were extracted independently by two reviewers (LA and AK) using a custom data extraction form. Participant baseline characteristics were extracted, including age and sex. Raw data on medication adherence were extracted either from the published articles or by contacting the authors of the primary studies by e-mail. When two measures of adherence were reported, that which was considered most comparable to the outcome measures of the other included studies was used for meta-analysis. For example, one study reported adherence according to both the number of times an electronically chipped dispenser was actioned by the patient and the weight of the dispensing cannister at study follow up and the reviewers agreed that the action of dispensing was most comparable to that of the other included studies.[21] Authors were contacted by email for any other data required which was not available in the published articles. BCTs that were utilised in the app-based interventions were coded according to the Behaviour Change Taxonomy.[22] This is a structured taxonomy of techniques utilised by interventions to change behaviour. The BCT Taxonomy does not have a code relating to performance of behaviour and therefore where participants were required to enter whether or not medication was taken, this was coded as 'Report whether or not the behaviour was performed'.

The included trials were assessed for risk of bias using the Cochrane Risk of Bias Tool.[23] Two reviewers (LA and AK) individually assessed each of the included articles against criteria outlined in the tool. Any discrepancies at any stage in the coding process were settled through discussion between the three reviewers (LA, AK, SS). Studies were considered at unclear risk of bias for an item when the primary article did not provide sufficient information for the reviewers to decide categorically between high or low risk of bias.

Statistical Analysis

Outcome data on medication adherence was extracted from each of the included studies at the final observation point. Authors were contacted for raw final observation point adherence data if this was

For peer review only - http://bmjopen.bmj.com/site/about/guidelines.xhtml

not reported in published manuscripts. Trials were considered homogenous based on the content of the intervention and comparator groups and the outcome measurement of medication adherence. Comprehensive Meta-Analysis software[24] was used to investigate the overall effect of app-delivered interventions on medication adherence using a random-effects model. The same software was used to perform meta-regression using the behaviour change techniques utilised by the applications. Comprehensive Meta-analysis Software conversion function was used where required to convert extracted raw data into odds ratio.[25] The I^2 calculation was used to estimate heterogeneity between included studies.

RESULTS

The initial electronic database search identified 13 295 potentially eligible studies and two were identified from reference lists of identified articles. After removal of duplicates 9971 citations were screened by title and abstract. We considered 80 of these to be eligible and retrieved the full-text articles. Of these, nine studies reported outcome data eligible for meta-analysis[14–19,21,26,27]. The screening process and reasons for exclusion are reported in Figure 1.

Study characteristics of the nine studies are displayed in Table 1. Dates of publication of included studies ranged from 2014-2018 Mean age in the intervention groups ranged from 20.3 (SD 4.0)[16] to 70.9 (SD 8).[18] One did not report the mean age but presented the percentage of participants whose age fell within a series of bandwidths.[21] The percentage of participants in intervention groups who were female ranged from 24%[17] to 57.4%.[19]

A number of measures of medication adherence were utilised by the included studies. Of the 9 studies, four measured the 8 Item Moriksy Medication Adherence Scale[15,17,19,28], with one of these also measuring a medication possession ratio, defined as the percentage of time the participant had access to the prescribed medication. [15] A further study measured the 4 Item

For peer review only - http://bmjopen.bmj.com/site/about/guidelines.xhtml

Morisky Medication Adherence Scale.[18] Of the remaining studies, one reported adherence according to pill count[16,26], one the A14 self-report scale of medication adherence[14] and one through a digital chip record fitted within the medication dispenser.[21]

Health problem of the target population varied between studies. Three studies sampled a patient population with cardiovascular disease[14,15,27] and each of the remaining six studies enrolled patient populations with different health problems (stroke[26], depression[16], Parkinson's disease,[17] hypertension,[19] psoriasis[21] and multimorbidity[18]).

Outcome Measurement Methods

A variety of methods were utilised across studies to measure medication adherence. Methods of outcome measurement included medication adherence self-reported scales,[14,17–19,27] medication possession ratio,[15] pill counts[16,26] and chip monitored medication dispensation from a cannister[29] (Table 1).

Meta-analysis

Meta-analysis was performed, pooling results from the nine eligible studies which overall reported data on 1005 participants, to estimate the effect of mobile applications to improve medication adherence.[14–19,21,26,27] The result of this meta-analysis indicates that patients who participate in medication adherence interventions delivered by mobile applications are more likely to adhere to prescribed medications (OR 2.120, 95% CI 1.635-2.747) than those that do not use such interventions. The I^2 value was 9.8 indicating low statistical heterogeneity between the studies included in the meta-analysis. The forest plot of the meta-analysis can be seen in Figure 2. The weighting of each individual study in the meta-analysis can be seen in eTable 1 and raw data extracted for the meta-analysis can be found in eTable 2.

For peer review only - http://bmjopen.bmj.com/site/about/guidelines.xhtml

A further meta-analysis was performed, pooling results from the 5 studies which reported medication adherence according to the Morisky Medication Adherence Scale.[15,17–19,27] The result of this also indicates that patients using medication adherence interventions delivered by mobile applications are more likely to adhere to prescribed medications (OR 1.83, 95% CI 1.42-2.36).

Behaviour Change Techniques

The most common BCT utilised by applications was 'Tailored", which was utilised by all studies. The next most common was 'Prompts and Cues' which was utilised by seven studies[14,16–18,21,26,27] and 'Report whether or not the behaviour was performed' which was utilised by six studies.[14,16–18,26,27] Agreement between reviewers for classification of Behaviour Change Techniques utilised by the interventions was assessed using the kappa statistic. This revealed a 'very good' level of agreement (kappa = 0.902, 95% CI 0.835 – 0.969).

Risk of bias at primary study level

A summary of the risk of bias analysis can be seen in Table 2**.** Overall, all studies were deemed as unclear in their risk of bias.

The most common source of risk of bias amongst the studies was the absence of blinding of participants and personnel to the outcome measure. The second most common risk of bias arose from a lack of objective measurements of medication adherence. This was due to reliance on participant self-report of adherence in several studies: five of the studies included in the meta-analysis reported adherence based on a self-assessment scale[14,17–19,27], and one study each used patient-entered medication logs in an app[16], an electronically chipped medicated foam dispenser[21], pill counts at clinic visits[26] and a medication possession ratio.[15]

Self-assessment scales are a form of self-report and therefore are a source of potential bias, including those of social desirability, and those arising from selective recall and the duration of the

For peer review only - http://bmjopen.bmj.com/site/about/guidelines.xhtml

recall period.[30] Two studies included in this review, which measured adherence through participant self-report, sought to compare their subjective measurements of adherence with serum measurements of either drug-reactivity units or a medication's anticoagulant effect.[15,26] One study showed that serum measurement of the medication's anticoagulant effect was similar between both the intervention and comparator groups, despite adherence measures being better in the intervention group.[26] The other study revealed that drug-reactivity units were worse in the intervention group than the control group, which conflicted with measured adherence outcome data.[15] However, the presence of a drug biomarker does not equate to compliance and the absence does not equate to non-compliance.[31] Indeed, drug metabolism should be taken into account for such measures of medication adherence, as physiological state and metabolic rate vary among individuals.[32]

Publication Bias

A funnel plot of effect size estimates of each of the studies included in the meta-analysis is provided in the online supplementary file (eFigure 1). Formal analysis of plot symmetry was not performed owing to the total number of studies included being less than 10.[33] Visual inspection reveals moderate symmetry of the plot.

DISCUSSION

Principle Findings

We conducted a rigorous systematic review of the literature to investigate whether mobile device applications are effective at supporting medication adherence. We identified nine studies eligible for inclusion in the meta-analysis.[14–19,26] All nine studies reported that their mobile app-delivered interventions were effective at supporting medication adherence although in five studies the interventions did not have a statistically significant effect (see Figure 2).[14–16,18,27] A random-effects meta-analysis of the pooled results from all nine studies indicated that people who use

For peer review only - http://bmjopen.bmj.com/site/about/guidelines.xhtml

For peer review only

medication reminder apps are significantly more likely to adhere to their medications than those who do not.

Intervention effects reported between the studies varied, as shown in the forest plot (Figure 2). Although the I^2 measure of statistical heterogeneity was low, and meta-regression of the BCTs utilised by the studies was not able to account for variance in effect observed between studies, there are other potential sources of methodological and clinical heterogeneity which may account for this. One potential explanation for this might be the variation in adherence measurement. This may be illustrated by three studies which incorporated five of the same BCTs[17,18,26] but where one[26] reported better medication adherence (OR 10.633, 95% CI 2.339-48.344) than the other two (OR 1.946, 95% CI 1.094-3.461 and OR 1.251, 95% CI 0.612-2.559).[17,18] In Labovitz et al's study, the medication adherence was measured by pill counts at clinic follow up visits[26], whereas in the other two studies, adherence was measured by retrospective self-scoring on a medication adherence scale[17,18] It could be argued that both of these different measures of adherence are vulnerable to bias with pill counts potentially being vulnerable to expectation bias and retrospective self-scores being vulnerable to recall bias. We subsequently performed a focused meta-analysis of the five studies which measured adherence using the Morisky Medication Adherence Scale to investigate whether this influenced the effect size observed. Whilst the result of meta-analysis remained significant, the effect size was lower. This may support the use of real-time or prospective adherence monitoring, over retrospective self-report for future research in the field of medication adherence.

A further possible explanation for variance in effect size might be techniques, beyond those of behaviour change, utilised by the apps. For example, in the study by Labovitz et al, the application intervention employed artificial intelligence to identify the participant, their medication and ingestion of the medication, using the camera of the mobile device and software algorithms. This could be regarded as a highly tailored intervention and it one might hypothesis that the degree of

For peer review only

For peer review only - http://bmjopen.bmj.com/site/about/guidelines.xhtml

tailoring could influence the effect size observed. A further example of a highly tailored intervention was that seen in the study by Lakshminarayana et al. All of the app-based interventions in the included studies were tailored to the prescribed medication regimen, but in this instance the tailoring went beyond the prescribed medication regimen, to include tailoring to the patients' intentional and non-intentional non-adherence, beliefs about medication taking, mood, cognitive impairment, symptom control, quality of life, age and disease duration. A significant effect size was observed in this study and this may support a hypothesis that highly tailored apps are more effective at supporting medication adherence, than or minimally tailored apps.

Strengths and Weaknesses of the Primary Studies

Based on the assessment of the risk of bias undertaken as part of this review, the evidence included in this meta-analysis is of moderate quality. The most common weaknesses in this evidence are small sample size, lack of objectivity of the outcome measurement, a lack of blinding of participants and personnel and limited follow up time.

Strengths and Weaknesses at Review Level

The pooling of data from a group of studies which used a variety of methods for measurement of medication adherence, targeted at patients with a variety of healthcare problems is a limitation of this review. As further studies in this field are published it will become more feasible to conduct subgroup analysis and meta-regression against variables such as age, health condition, targeted medication, intervention content and application characteristics. A standardised protocol for measuring adherence and reporting the content and characteristics of application interventions may facilitate further analysis of the latter two variables.

This review cannot report on the likely sustainability of the effect on medication adherence given the short study duration of the studies included in this review; across the 9 studies, the maximum follow up period was 16 weeks.[17] Furthermore, the optimal frequency and duration of

For peer review only - http://bmjopen.bmj.com/site/about/guidelines.xhtml

application use that is required to achieve improvements in medication adherence is not known. Long term and large-scale research is required in this field to addresses the question regarding sustainability of the observed effect found in this meta-analysis.

This systematic review and meta-analysis included studies with a range of ages of participants. The lowest mean age of participants in the included studies was 20.9 years[16] and the highest was 70.9 years. The ubiquity and use of mobile devices varies across age groups[9] and further research to establish the acceptability and usability of adherence applications among older adults would be valuable.

Finally, the reviewers set out to perform a meta-regression of the BCTs utilised by application interventions in the included studies.[13] However, this did not reveal any significant association between the BCTs utilised and observed effect size. This may be owing to the low estimate of heterogeneity and the small number of eligible included studies.

Conclusions

This meta-analysis indicates that medication adherence interventions delivered by smartphone applications can be effective at improving patients' adherence to their prescribed medications. However, when considering whether to recommend an application to patients, these results should be interpreted with caution owing to variance in effect size seen between the studies, which all investigated different applications. Further research should aim to first establish a standard protocol for the measuring and reporting of adherence in the context of evaluating digital interventions. Subsequently, further research investigating intervention characteristics associated with an application's effect and the sustainability of any effect gained would add to this field.

.

For peer review only - http://bmjopen.bmj.com/site/about/guidelines.xhtml

References

1 Osterberg L, Blaschke T. Adherence to Medication. *N Engl J Med* 2005;**353**:487–97. doi:10.1056/NEJMra050100

2 Brown MT, Bussell JK. Medication Adherence: WHO Cares? *Mayo Clin Proc* 2011;**86**:304. doi:10.4065/MCP.2010.0575

3 Elliott RA, Boyd MJ, Waring J, *et al.* Understanding & evaluating the New Medicines Service in the NHS in England (029/0124). 2014. http://www.nottingham.ac.uk/~pazmjb/nms/downloads/report/files/assets/common/downloads/108842 A4 Main Report.v4.pdf (accessed 12 Feb 2018).

4 Iuga AO, McGuire MJ. Adherence and health care costs. *Risk Manag Healthc Policy* 2014;**7**:35–44. doi:10.2147/RMHP.S19801

5 Sabaté E. Evidence for action. *World Heal Organ* Published Online First: 2003.http://www.who.int/chp/knowledge/publications/adherence_introduction.pdf (accessed 12 Feb 2018).

6 Ofcom. Communications Market Report. 2018. https://www.ofcom.org.uk/__data/assets/pdf_file/0022/117256/CMR-2018-narrative-report.pdf (accessed 3 Aug 2018).

7 Dagli RJ, Sharma A. Polypharmacy: a global risk factor for elderly people. *J Int oral Heal JIOH* 2014;**6**:i–ii.http://www.ncbi.nlm.nih.gov/pubmed/25628499 (accessed 20 May 2018).

8 Smith A. Nearly half of American adults are smartphone owners. 2012. http://www.pewinternet.org/2012/03/01/nearly-half-of-american-adults-are-smartphone-owners/ (accessed 9 Jun 2018).

For peer review only - http://bmjopen.bmj.com/site/about/guidelines.xhtml

9 Ofcom. The Communications Market Report. 2015. https://www.ofcom.org.uk/__data/assets/pdf_file/0022/20668/cmr_uk_2015.pdf (accessed 12 Feb 2018).

10 Ahmed I, Ahmad NS, Ali S, *et al.* Medication Adherence Apps: Review and Content Analysis. *JMIR mHealth uHealth* 2018;**6**:e62. doi:10.2196/mhealth.6432

11 Wideman TH, Sullivan MJL, Inada S, *et al.* Behaviour Change Techniques. In: Gellman Marc D., Turner J. Rick., eds. *Encyclopedia of Behavioral Medicine*. New York, NY: : Springer New York 2013. 12. doi:10.1007/978-1-4419-1005-9_1661

12 Moher D, Liberati A, Tetzlaff J, *et al.* Preferred Reporting Items for Systematic Reviews and Meta-Analyses: The PRISMA Statement. *PLoS Med* 2009;**6**:e1000097. doi:10.1371/journal.pmed.1000097

13 Armitage L, Kassavou A, Sutton S. A systematic review of the literature with met-analysis and meta-regression: the use of smartphone applications to support medication adherence/. PROSPERO Int. Prospect. Regist. Syst. Rev. 2017. doi:CRD42017080150

14 Mertens A, Brandl C, Miron-shatz T, *et al.* A mobile application improves therapy-adherence rates in elderly patients undergoing rehabilitation. *Medicine (Baltimore)* 2016;**95**:e4446.

15 Shah V, Dileep A, Dickens C, *et al.* Patient-Centered Tablet Application for Improving Medication Adherence after a Drug-Eluting Stent. *Front public Heal* 2016;**4**:272. doi:10.3389/fpubh.2016.00272

16 Hammonds T, Rickert K, Goldstein C, *et al.* Adherence to Antidepressant Medications: A Randomized Controlled Trial of Medication Reminding in College Students. 2015;**8481**. doi:10.1080/07448481.2014.975716

For peer review only

For peer review only - http://bmjopen.bmj.com/site/about/guidelines.xhtml

17 Lakshminarayana R, Wang D, Burn D, *et al.* Using a smartphone-based self-management platform to support medication adherence and clinical consultation in Parkinson ' s disease. *NPJ Park Dis* 2017;**3**:1–8. doi:10.1038/s41531-016-0003-z

18 Mira JJ, Navarro I, Botella F, *et al.* A Spanish pillbox app for elderly patients taking multiple medications: randomized controlled trial. *J Med Internet Res* 2014;**16**:e99. doi:10.2196/jmir.3269

19 Morawski K, Ghazinouri R, Krumme A, *et al.* Association of a Smartphone Application With Medication Adherence and Blood Pressure Control. *JAMA Intern Med* 2018;**178**:802. doi:10.1001/jamainternmed.2018.0447

20 DeVito Dabbs A, Song MK, Myers BA, *et al.* A Randomized Controlled Trial of a Mobile Health Intervention to Promote Self-Management After Lung Transplantation. *Am J Transplant* 2016;**16**:2172–80. doi:10.1111/ajt.13701

21 Svendsen MT, Andersen F, Andersen KH, *et al.* A smartphone application supporting patients with psoriasis improves adherence to topical treatment: a randomized controlled trial. *Br J Dermatol* 2018;**179**:1062–71. doi:10.1111/bjd.16667

22 Michie S, Richardson M, Johnston M, *et al.* The Behavior Change Technique Taxonomy (v1) of 93 Hierarchically Clustered Techniques: Building an International Consensus for the Reporting of Behavior Change Interventions. *Ann Behav Med* 2013;**46**:81–95. doi:10.1007/s12160-013-9486-6

23 Higgins JP, Altman DG, Sterne JA. Chapter 8: Assessing risk of bias in included studies. https://training.cochrane.org/handbook/chapter-08 (accessed 14 Feb 2018).

24 Comprehensive Meta-Analysis Software (CMA). https://www.meta-analysis.com/ (accessed 15 Feb 2018).

For peer review only

For peer review only - http://bmjopen.bmj.com/site/about/guidelines.xhtml

25 Borenstein M, Hedges L., Higgins PT, *et al. Introduction to Meta-Analysis, Converting Among Effect Sizes*. John Wiley and Sons 2009. https://www.meta-analysis.com/downloads/Meta-analysis Converting among effect sizes.pdf (accessed 8 Mar 2019).

26 Labovitz DL, Shafner L, Gil MR, *et al.* Using Artificial Intelligence to Reduce the Risk of Nonadherence in Patients on Anticoagulation Therapy. *Stroke* 2017;**48**:1416–9. doi:10.1161/STROKEAHA.116.016281

27 Santo K, Singleton A, Rogers K, *et al.* Medication reminder applications to improve adherence in coronary heart disease: a randomised clinical trial. *Heart* 2018;:heartjnl-2018-313479. doi:10.1136/heartjnl-2018-313479

28 Santo K, Chow CK, Thiagalingam A, *et al.* MEDication reminder APPs to improve medication adherence in Coronary Heart Disease (MedApp-CHD) Study : a randomised controlled trial protocol. 2017;:1–10. doi:10.1136/bmjopen-2017-017540

29 Svenson JE, Repplinger M. Hypertension in the ED: still an unrecognized problem. *Am J Emerg Med* 2008;**26**:913–7. doi:10.1016/j.ajem.2007.11.024

30 Althubaiti A. Information bias in health research: definition, pitfalls, and adjustment methods. *J Multidiscip Healthc* 2016;**9**:211–7. doi:10.2147/JMDH.S104807

31 Farmer KC. Methods for measuring and monitoring medication regimen adherence in clinical trials and clinical practice. *Clin Ther* 1999;**21**:1074–90. doi:10.1016/S0149-2918(99)80026-5

32 Lam WY, Fresco P. Medication Adherence Measures: An Overview. *Biomed Res Int* 2015;**2015**:217047. doi:10.1155/2015/217047

33 Sterne JAC, Sutton AJ, Ioannidis JPA, *et al.* Recommendations for examining and interpreting funnel plot asymmetry in meta-analyses of randomised controlled trials. *BMJ*

For peer review only

For peer review only - http://bmjopen.bmj.com/site/about/guidelines.xhtml

2011;**343**:d4002. doi:10.1136/BMJ.D4002

Sources of funding

This paper presents independent research funded by the National Institute for Health Research (NIHR) under its Programme Grants for Applied Research Programme (Grant Reference Number RP-PG-0615-20013). The views expressed are those of the authors and not necessarily those of the NIHR or the Department of Health and Social Care. The National Institute for Health Research's (NIHR) Collaboration for Leadership in Applied Health Research and Care (CLAHRC) in East of England provided salary support to LA during conduction of this review. The funders had no role in review design, data collection, data analysis, data interpretation, the writing of the manuscript, and decision to submit the manuscript for publication.

Author Contributions

SS All authors were involved in design of the review and undertook methodological planning. LA performed and refined the searches in consultation with a medical librarian. LA and KK performed initial screening and data extraction and SS gave screening advice where any disagreements arose. All authors contributed to performing the meta-analysis to data interpretation. LA led the writing and all authors contributed to successive drafts and approved the final manuscript.

Competing Interests

The authors have no competing interests to declare

Figure Legends

Figure 1: PRISMA Flowchart

For peer review only

For peer review only - http://bmjopen.bmj.com/site/about/guidelines.xhtml

Figure 2. Forest Plot of Randomised Trials evaluating a mobile device application to support medication adherence against usual care.

For peer review only

For peer review only - http://bmjopen.bmj.com/site/about/guidelines.xhtml

Table 1: Study Characteristics

Source (Sample size)	Study Nation	Study Design	Health Problem of Target Population	Recruitment setting	Participant characteristics at recruitment				Application intervention	Outcome measure	Follow up interval
					Intervention group		Comparator group				
					Mean age (SD)	% female	Mean age (SD)	Female, N (%)			
Hammonds et al, 2015 (40)	USA	RCT	Depression	College students were recruited via campus based advertisements and the university research recruitment system	20.3 (4.0)	25 (83)	20.9 (4.7)	24 (89)	Medication reminder app	Pill count	25-35d
Labowitz et al, 2017 (27)	USA	RCT	Ischaemic stroke requiring secondary oral anti-coagulation	Outpatient clinic	58.3 (9.79)	6 (40)	55.5 (16.55)	9 (69)	Artificial intelligence app	Pill count	12w
Lakshmin-arayana et al, 2017 (158)	UK	RCT	Parkinson's disease	outpatient clinic	59.86 (9.13)	34 (36.2)	60.71 (10.26)	45 (42.1)	Parkinson's tracker app	8 Item MMAS	16w
Mertens et al, 2016 (24)	Germany	RCT Cross-over	Cardiovascular disease	Cardiac rehab groups	73.8[a]	12 (50)[a]	73.8[a]	12 (50)[a]	Medication Plan app (version 1.3)	Self-report:A14 scale	28d
Mira et al, 2014 (99)	Spain	RCT	Multi-comorbidity	Community health centres	70.9 (8)	21 (41)	72.9 (6)	23 (48)	ALICE medication self-management app	MMAS 4	3m

For peer review only - http://bmjopen.bmj.com/site/about/guidelines.xhtml

Morawski et al, 2018 (412)	USA	RCT	Hypertension	Social media, mobile apps, targeted advertisements and online communications	51.7 (10.5)	120 (57.4)	52.4 (10.1)	127 (62.9)	MediSAFE-BP app	8 Item MMAS	12w
Santo et al, 2018 (101)	Australia	RCT	Cardiovascular Disease	Inpatient, outpatient and rehabilitative care settings.	60.2 (9.04)	14 (13)	56.8 (8.74)	6 (11)	MedApp-CHD	8 Item MMAS	3m
Shah et al, 2016 (24)	USA	RCT	Cardiovascular stent	Patients in the immediate post-operative phase of insertion of a drug-eluting cardiovascular stent	60.5 (8.9)	6 (46)	60.6 (4.1)	4 (36)	MyIDEA education app	Medication possession ratio and MMAS?	90d
Svendsen et al, 2018 (120)	Denmark	RCT	Psoriasis	Hospital posters and the dermatology outpatient clinic.	18-40 yrs 29[b]<br>41-50 21<br>51-60 26<br>61-75 24	27 (40)	18-40 yrs 32[b]<br>41-50 20<br>51-60 24<br>61-75 24	25 (38)	My Pso SmarTop Version 1.0 or Smartop number 0536776	Measured via a digital chip record in the medication dispenser.	38d

MMAS = Morisky Medication Adherence Scale.
[a]Baseline characteristics presented for whole cohort as study design was a randomised cross-over trial; [b]number of participants in each age group presented as mean age not available.

For peer review only - http://bmjopen.bmj.com/site/about/guidelines.xhtml

Table 2: Risk of Bias Summary Table

Source	Allocation sequence adequately:		Free from baseline imbalance?			Blinding of participants, personnel and objective outcome assessment	Incomplete outcome data adequately addressed	Free from selective outcome reporting	Was the intervention independent of other bias
	Generated	Concealed	Age	Gender	Adherence				
Hammonds et al, 2015									
Labowitz et al, 2017									
Lakshminarayana et al, 2017									
Mertens et al, 2016									
Mira et al, 2014									
Morawski et al, 2018									
Santo et al, 2018									
Shah et al, 2016									
Svendsen et al, 2018									

For peer review only - http://bmjopen.bmj.com/site/about/guidelines.xhtml

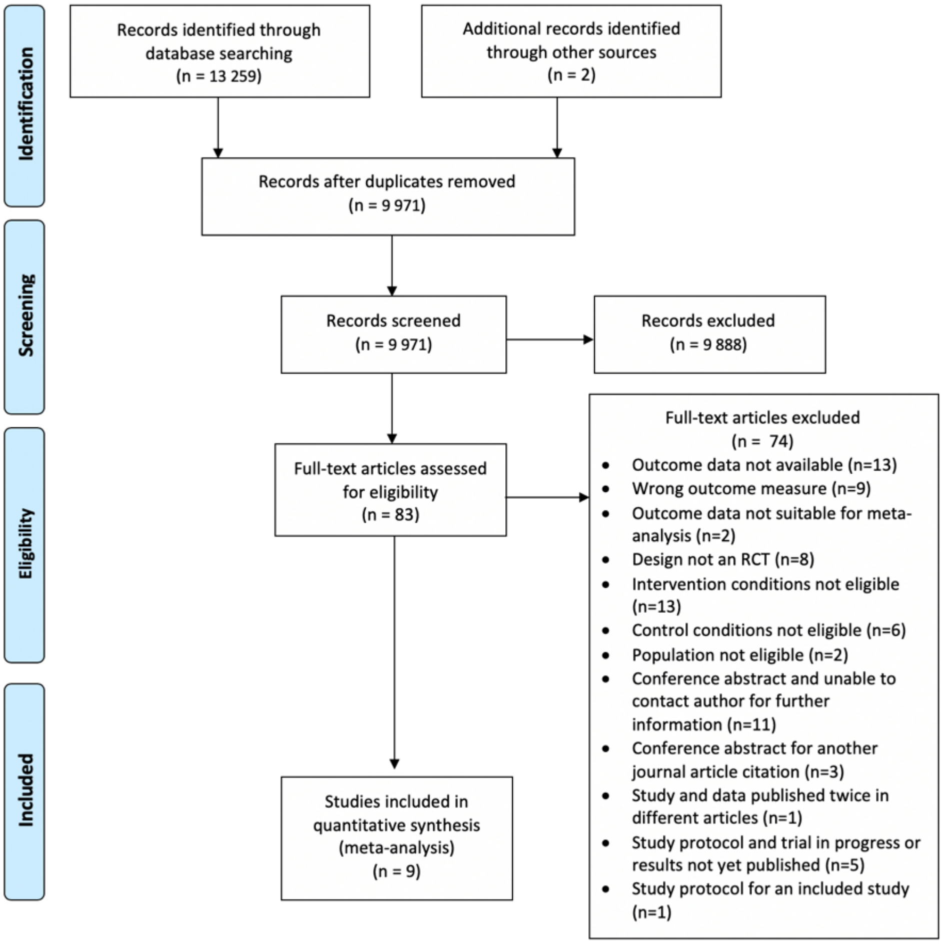

Figure 1: PRISMA Flowchart

Study name	Odds ratio	Lower limit	Upper limit	Z-Value	p-Value
Hammonds 2015	3.500	0.945	12.966	1.875	0.061
Labovitz 2017	10.633	2.339	48.344	3.060	0.002
Lakshminarayana 2017	1.946	1.094	3.461	2.266	0.023
Mertens 2016	2.975	0.675	13.117	1.440	0.150
Mira 2014	1.251	0.612	2.559	0.614	0.539
Morawski 2018	1.896	1.331	2.700	3.547	0.000
Shah 2016	2.206	0.505	9.626	1.052	0.293
Svendsen 2018	3.222	1.526	6.803	3.068	0.002
Santo 2018	2.029	0.991	4.154	1.934	0.053
	2.120	1.635	2.747	5.677	0.000

Statistics for each study

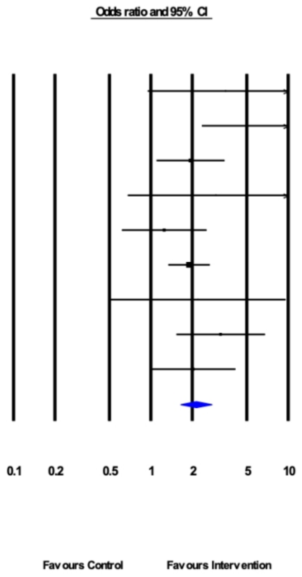

Figure 2. Forest Plot of Randomised Trials evaluating a mobile device application to support medication adherence against usual care.

SUPPLEMENTAL MATERIAL FILE

Mobile device applications to support medication adherence: A systematic review of randomised controlled trials, with meta-analysis

For peer review only

Author Details:

Laura Armitage, MRCGP

Aikaterini Kassavou, PhD

Stephen Sutton, PhD

For peer review only - http://bmjopen.bmj.com/site/about/guidelines.xhtml

eText 1: Medline/Pubmed Search Strategy

1. medication adherence.mp. or exp Patient Compliance/ or exp Medication Adherence/
2. nonadherence.mp.
3. non-adherence.mp.
4. non adherence.mp.
5. concordance.mp.
6. concordant.mp.
7. 1 or 2 or 3 or 4 or 5 or 6
8. app.mp.
9. apps.mp.
10. application.mp.
11. exp Mobile Applications/ or mobile application*.mp.
12. smartphone.mp. or exp Smartphone/ or exp Cell Phones/
13. mobile*.mp.
14. phone*.mp.
15. 8 or 9 or 10 or 11 or 12 or 13 or 14
16. randomized controlled trial.mp. or exp Randomized Controlled Trial/
17. RCT.mp.
18. randomised.mp.
19. randomized.mp.
20. control*.mp.
21. 16 or 17 or 18 or 19 or 20
22. 7 and 15 and 21
23. limit 22 to (english language and yr="1990 - 2017")

For peer review only

eFigure 1: Funnel Plot of effect estimates from studies included in meta-analysis

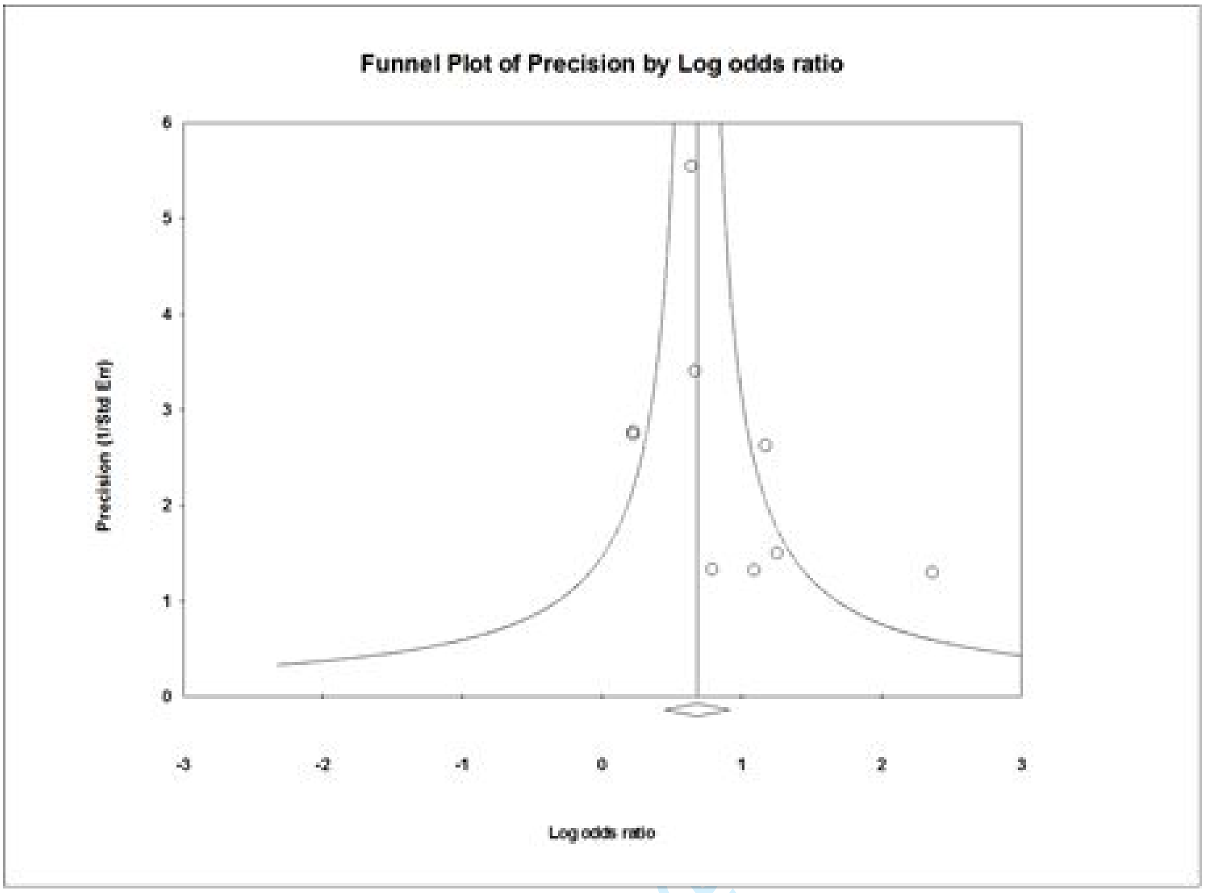

ew only

For peer review only - http://bmjopen.bmj.com/site/about/guidelines.xhtml

eTable 1: Relative weighting of each study included in the 9 study meta-analysis

Study	Weight (random)	Relative weight
Hammonds et al, 2015	2.16	3.79
Labovitz et al, 2017	1.63	2.86
Lakshminarayana et al, 2017	9.76	17.10
Mertens et al, 2018	1.70	2.97
Mira et al, 2014	6.69	11.72
Morawski et al, 2018	20.58	36.03
Santo et al, 2018	6.68	11.69
Shah et al, 2016	1.72	3.01
Svendsen et al, 2018	6.19	10.84

For peer review only

eTable 2: Raw data extracted for each study for meta-analysis

Study	Adherence measurement	Intervention group				Comparator group			
		Mean (SD)	N	Events	Totals	Mean (SD)	N	Events	Totals
Hammonds et al, 2015	No. participants taken ≥80% prescribed tablets per day	-	-	12	20	-	-	6	20
Labovitz et al, 2017	Mean cumulative adherence, measured by pill count	97.2 (4.4)	15	-	-	90.6 (5.8)	12	-	-
Lakshminaray-ana et al, 2017	MMAS-8	6.3 (1.52)	68	-	-	5.74 (1.53)	90	-	-
Mertens et al, 2018	A14 Scale	53.96 (2.01)	12	-	-	52.6 (2.49)	12	-	-
Mira et al, 2014	MMAS-4	7.4 (0.9)	51	-	-	7.3 (0.7)	48	-	-
Morawski et al, 2018	MMAS-8	6.3 (1.6)	210	-	-	5.74 (1.53)	90	-	-
Santo et al, 2018	MMAS-8	7.02 (n/a)	50	-	-	6.63 (n/a)	51	-	-
Shah et al, 2016	MMAS-8	2.2 (1.2)	5	-	-	2.1 (1)	3	-	-
Svendsen et al, 2018	No. of patients having applied medication ≥80% dats in treatment period	-	-	39	59	-	-	23	61

A14 = A14 Medication Adherence Scale; MMAS-8 = 8 Item Morisky Medication Adherence Scale; MMAS 4 = 4 Item Morisky Medication Adherence Scale

For peer review only - http://bmjopen.bmj.com/site/about/guidelines.xhtml

PRISMA 2009 Checklist

Section/topic	#	Checklist item	Reported on page #
**TITLE**			
Title	1	Identify the report as a systematic review, meta-analysis, or both.	1
**ABSTRACT**			
Structured summary	2	Provide a structured summary including, as applicable: background; objectives; data sources; study eligibility criteria, participants, and interventions; study appraisal and synthesis methods; results; limitations; conclusions and implications of key findings; systematic review registration number.	2-3
**INTRODUCTION**			
Rationale	3	Describe the rationale for the review in the context of what is already known.	4-5
Objectives	4	Provide an explicit statement of questions being addressed with reference to participants, interventions, comparisons, outcomes, and study design (PICOS).	6
**METHODS**			
Protocol and registration	5	Indicate if a review protocol exists, if and where it can be accessed (e.g., Web address), and, if available, provide registration information including registration number.	5
Eligibility criteria	6	Specify study characteristics (e.g., PICOS, length of follow-up) and report characteristics (e.g., years considered, language, publication status) used as criteria for eligibility, giving rationale.	6
Information sources	7	Describe all information sources (e.g., databases with dates of coverage, contact with study authors to identify additional studies) in the search and date last searched.	5
Search	8	Present full electronic search strategy for at least one database, including any limits used, such that it could be repeated.	Supplementary file p 2
Study selection	9	State the process for selecting studies (i.e., screening, eligibility, included in systematic review, and, if applicable, included in the meta-analysis).	6
Data collection process	10	Describe method of data extraction from reports (e.g., piloted forms, independently, in duplicate) and any processes for obtaining and confirming data from investigators.	6-7
Data items	11	List and define all variables for which data were sought (e.g., PICOS, funding sources) and any assumptions and simplifications made.	6-8 and Table 1, p21
Risk of bias in individual studies	12	Describe methods used for assessing risk of bias of individual studies (including specification of whether this was done at the study or outcome level), and how this information is to be used in any data synthesis.	7
Summary measures	13	State the principal summary measures (e.g., risk ratio, difference in means).	7
Synthesis of results	14	Describe the methods of handling data and combining results of studies, if done, including measures of consistency (e.g., I^2) for each meta-analysis.	7-8

For peer review only

For peer review only - http://bmjopen.bmj.com/site/about/guidelines.xhtml

PRISMA 2009 Checklist

Section/topic	#	Checklist item	Reported on page #
Risk of bias across studies	15	Specify any assessment of risk of bias that may affect the cumulative evidence (e.g., publication bias, selective reporting within studies).	7
Additional analyses	16	Describe methods of additional analyses (e.g., sensitivity or subgroup analyses, meta-regression), if done, indicating which were pre-specified.	7
**RESULTS**			
Study selection	17	Give numbers of studies screened, assessed for eligibility, and included in the review, with reasons for exclusions at each stage, ideally with a flow diagram.	8
Study characteristics	18	For each study, present characteristics for which data were extracted (e.g., study size, PICOS, follow-up period) and provide the citations.	P8-9 and Table 1, p 21
Risk of bias within studies	19	Present data on risk of bias of each study and, if available, any outcome level assessment (see item 12).	P10 and Table 2, p 23
Results of individual studies	20	For all outcomes considered (benefits or harms), present, for each study: (a) simple summary data for each intervention group (b) effect estimates and confidence intervals, ideally with a forest plot.	Figure 2,<br>(and eTable 1 for weighting of each study and eTable 2 for raw extracted data found in supplementary file, p 4-5)
Synthesis of results	21	Present results of each meta-analysis done, including confidence intervals and measures of consistency.	9
Risk of bias across studies	22	Present results of any assessment of risk of bias across studies (see Item 15).	11 and efigure 1 on p 3 of supplementary material file
Additional analysis	23	Give results of additional analyses, if done (e.g., sensitivity or subgroup analyses, meta-regression [see Item 16]).	11
**DISCUSSION**			

For peer review only

PRISMA 2009 Checklist

Summary of evidence	24	Summarize the main findings including the strength of evidence for each main outcome; consider their relevance to key groups (e.g., healthcare providers, users, and policy makers).	11
Limitations	25	Discuss limitations at study and outcome level (e.g., risk of bias), and at review-level (e.g., incomplete retrieval of identified research, reporting bias).	13
Conclusions	26	Provide a general interpretation of the results in the context of other evidence, and implications for future research.	14
**FUNDING**			
Funding	27	Describe sources of funding for the systematic review and other support (e.g., supply of data); role of funders for the systematic review.	19

From: Moher D, Liberati A, Tetzlaff J, Altman DG, The PRISMA Group (2009). Preferred Reporting Items for Systematic Reviews and Meta-Analyses: The PRISMA Statement. PLoS Med 6(7): e1000097. doi:10.1371/journal.pmed1000097

For more information, visit: **www.prisma-statement.org**.

For peer review only

For peer review only - http://bmjopen.bmj.com/site/about/guidelines.xhtml

BMJ Open

Are mobile device applications designed to support medication adherence effective? A systematic review of randomised controlled trials, with meta-analysis

Journal:	*BMJ Open*
Manuscript ID	bmjopen-2019-032045.R1
Article Type:	Original research
Date Submitted by the Author:	03-Oct-2019
Complete List of Authors:	Armitage, Laura; University of Oxford, Nuffield Department of Primary Care Health Sciences;<br>Kassavou, Aikaterini; University of Cambridge, Public Health and Primary Care ; University of Cambridge,<br>Sutton, Stephen; University of Cambridge,
**Primary Subject Heading**:	Public health
Secondary Subject Heading:	General practice / Family practice
Keywords:	Telemedicine < BIOTECHNOLOGY & BIOINFORMATICS, PRIMARY CARE, PUBLIC HEALTH, World Wide Web technology < BIOTECHNOLOGY & BIOINFORMATICS, GENERAL MEDICINE (see Internal Medicine)

SCHOLARONE™
Manuscripts

I, the Submitting Author has the right to grant and does grant on behalf of all authors of the Work (as defined in the below author licence), an exclusive licence and/or a non-exclusive licence for contributions from authors who are: i) UK Crown employees; ii) where BMJ has agreed a CC-BY licence shall apply, and/or iii) in accordance with the terms applicable for US Federal Government officers or employees acting as part of their official duties; on a worldwide, perpetual, irrevocable, royalty-free basis to BMJ Publishing Group Ltd ("BMJ") its licensees and where the relevant Journal is co-owned by BMJ to the co-owners of the Journal, to publish the Work in this journal and any other BMJ products and to exploit all rights, as set out in our licence.

The Submitting Author accepts and understands that any supply made under these terms is made by BMJ to the Submitting Author unless you are acting as an employee on behalf of your employer or a postgraduate student of an affiliated institution which is paying any applicable article publishing charge ("APC") for Open Access articles. Where the Submitting Author wishes to make the Work available on an Open Access basis (and intends to pay the relevant APC), the terms of reuse of such Open Access shall be governed by a Creative Commons licence – details of these licences and which Creative Commons licence will apply to this Work are set out in our licence referred to above.

Other than as permitted in any relevant BMJ Author's Self Archiving Policies, I confirm this Work has not been accepted for publication elsewhere, is not being considered for publication elsewhere and does not duplicate material already published. I confirm all authors consent to publication of this Work and authorise the granting of this licence.

For peer review only

Title

Are mobile device applications designed to support medication adherence effective? A systematic review of randomised controlled trials, with meta-analysis

Word Count of Manuscript Text: 3432

Author Details:

Laura Armitage, MRCGP[1,2,3]

Aikaterini Kassavou, PhD[1]

Stephen Sutton, PhD[1]

Affiliations:

1. Department of Public Health and Primary Care, University of Cambridge, UK
2. National Institute for Health Research, Collaboration for Leadership in Applied Health Research and Care, East of England, UK
3. Nuffield Department of Primary Care Health Sciences, University of Oxford, UK

Author for Correspondence:

Laura Armitage

Email: laura.armitage@phc.ox.ac.uk Tel: +447791366355

Nuffield Department of Primary Care Health Sciences,

Radcliffe Primary Care Building, Radcliffe Observatory Quarter,

Woodstock Road,

Oxford,

OX2 6GG

For peer review only

For peer review only - http://bmjopen.bmj.com/site/about/guidelines.xhtml

ABSTRACT

Objectives

Estimate the effectiveness of app-based interventions designed to support medication adherence and establish which behaviour change techniques utilised by applications are associated with efficacy.

Design

Systematic review of randomised controlled trials (RCTs), with meta-analysis.

Setting

Medline/PubMed, PsycINFO, CINAHL, Embase and Web of Science databases were searched from 1990 to November 2018 for RCTs investigating efficacy of a mobile application to support medication adherence versus usual care, in any healthcare setting.

Participants

Studies of participants of any age, taking prescribed medication for any health condition, for any duration were eligible for inclusion.

Intervention

An application-based intervention delivered through a smartphone, tablet computer or personal desktop assistant to help, support or advise about medication adherence.

Primary and Secondary Outcome Measures

The primary outcome was the pooled effect size of changes in medication adherence. The secondary outcome was to investigate for an association between behaviour change techniques utilised by applications and effect size.

For peer review only

For peer review only - http://bmjopen.bmj.com/site/about/guidelines.xhtml

Results

The initial search identified 13 259 citations. After title and abstract screening, full text articles of 83 studies were screened for eligibility. Nine RCTs with 1159 recruited participants were included in this review. Mean age of participants was >50 years in all but one study. Health conditions of the target populations included cardiovascular disease, depression, Parkinson's disease, psoriasis and multimorbidity. Meta-analysis indicated that patients who use mobile applications to support medication taking are more likely to adhere to medications (OR 2.120, 95% CI 1.635-2.747, N=988) in comparison to usual care. Meta-regression of the behaviour change techniques did not reveal any significant associations with effect size.

Conclusions

App-based medication adherence interventions may have a positive effect on patient adherence. Larger scale studies are required to further evaluate this effect, including its long-term sustainability and characteristics of the intervention and participants which are associated with efficacy and app usage.

Article Summary – Strengths and Limitations of this Study

- This systematic review and meta-analysis of randomised controlled trials offers new information on the efficacy of mobile device applications to support medication adherence across a range of medical conditions.
- This review is reported according to the PRISMA Statement.
- A sensitive search strategy was designed and screening of articles and data extraction was performed independently by one clinical researcher and one non-clinical researcher.

For peer review only - http://bmjopen.bmj.com/site/about/guidelines.xhtml

- The review protocol was prospectively registered and the scope of the review, inclusion and exclusion criteria remained unchanged throughout.
- Two reviewers independently considered abstracts and full text articles for inclusion and extracted data and assessed included studies for risk of bias in the same way.

For peer review only

For peer review only - http://bmjopen.bmj.com/site/about/guidelines.xhtml

MANUSCRIPT TEXT

Introduction

Adherence to medication is defined as the extent to which patients take medications as prescribed and agreed with their health care providers.[1] The therapeutic benefit of prescribed medications is limited owing to an estimated 50% of patients not adhering to medications.[2] The annual cost of non-adherence is estimated exceed £930 million in England[3] and between $100 and $300 billion in the US.[4] Furthermore, in 2003 the Adherence to Long-term Therapy report published by the World Health Organisation stated that unless medication adherence is addressed, advances in biomedical technology will not achieve their potential.[5]

Interventions for improving adherence should be reliable, contemporary, acceptable and readily available to the person. Mobile devices can meet these needs and in 2018, Ofcom reported that 78% of adults and 95% of 16-24 year olds in the UK own a smartphone, demonstrating that smartphones are a growing part of modern life.[6] Patients who are prescribed multiple medications are commonly in the older age group, where the percentage of smartphone users may be lower However, smartphone ownership in those aged over 65 more than trebled between the years 2012 and 2015 from 5% to 18%, and is set to continue to increase. [7]

Hundreds of applications are now available to patients to support them in taking regular medication.[8] Such applications frequently utilise behaviour change techniques (BCTs) to promote improvements in adherence. A behaviour change technique is defined as an active component of an intervention that is designed to change behaviour.[9] To date, the effectiveness of smartphone apps to support patient adherence to regular prescribed medications has not been evaluated within the scientific literature using rigorous quantitative review. We have performed a novel systematic review with meta-analysis, to evaluate the effectiveness of smartphone applications in supporting medication adherence.

For peer review only

For peer review only - http://bmjopen.bmj.com/site/about/guidelines.xhtml

The objectives of this review are (i) to establish whether applications designed to support medication adherence are effective and (ii) to identify the intervention characteristics and Behavioural Change Techniques (BCTs) associated with effective applications. The conclusions drawn have potential to inform future large-scale public health interventions for the improvement of medication adherence.

METHODS

This systematic review is reported according to the PRISMA statement.[10] The protocol for the review is registered with and published on the PROSPERO database of systematic reviews (registration number: CRD42017080150).[11] The scope of the review, inclusion and exclusion criteria remained unchanged throughout.

Patient and Public Involvement

This research was done without patient involvement. Patients were not invited to comment on the study design and were not consulted to develop patient relevant outcomes or interpret the results. Patients were not invited to contribute to the writing or editing of this document for readability or accuracy.

Data Sources

Medline/PubMed, PsycINFO, CINAHL, Embase and Web of Science electronic databases were searched from 1990 to November 2018. A sensitive search strategy was designed based on preliminary searches, relevant papers and key words. Key search terms included adherence, non-adherence, smart phone application and randomised control trials (RCTs). An example detailed search strategy for the Medline/PubMed database can be found in supplementary information, (eText 1). Reference lists of included studies were screened for additional relevant trials.

For peer review only

For peer review only - http://bmjopen.bmj.com/site/about/guidelines.xhtml

Two reviewers (LA and AK) independently screened all citations by title and abstract and excluded those which were irrelevant or clearly did not meet eligibility criteria. Full text articles were obtained for all remaining citations and screened against the eligibility criteria by the same reviewers. Any discrepancies at any stage in the screening process were settled through discussion between LA, AK and with a third reviewer (SS).

Study Selection

RCTs relevant to this review were those which investigated the use of an application (app) on a mobile device to support medication adherence. Inclusion criteria were as follows:

(i) Participants of any age who were taking one or more medications prescribed for treatment of a medical condition.

(ii) An intervention group which received an app-based intervention delivered through a smartphone, tablet computer or personal desktop assistant to help, support or advise about medication adherence.

(iii) A comparator group which received usual care, another medication adherence intervention which did not utilise an app,[12] or an app which did not include any behaviour change techniques (for example an application that was a non-health related game[13]).

(iv) Medication adherence data reported for both the intervention and comparator groups.

Exclusion criteria were as follows:

(i) Complex interventions of which an application was just one component were not eligible.

(ii) Interventions delivered by text messaging or telemedicine.

(iii) Outcome assessed was adherence to treatment components other than prescribed medication. For example one study reported outcome data as a composite score,

For peer review only - http://bmjopen.bmj.com/site/about/guidelines.xhtml

combining adherence to other aspects of the medical regimen (such as clinic attendance and laboratory work) with adherence to medication.[14]

(iv) A study population of healthy recruits who were required to adhere to a placebo medication.

(v) Comparator group received an adherence intervention in another format, such as paper-based or a different application.

Published articles were required to be in English language.

Data Extraction and synthesis

Data were extracted independently by two reviewers (LA and AK) using a custom data extraction form. Participant baseline characteristics were extracted, including age and sex. Raw data on medication adherence were extracted either from the published articles or by contacting the authors of the primary studies by e-mail. When two measures of adherence were reported, that which was considered most comparable to the outcome measures of the other included studies was used for meta-analysis. For example, one study reported adherence according to both the number of times an electronically chipped dispenser was actioned by the patient and the weight of the dispensing cannister at study follow up and the reviewers agreed that the action of dispensing was most comparable to that of the other included studies.[15] Authors were contacted by email for any other data required which was not available in the published articles. Where more than one application intervention was tested, data for the intervention considered most comparable to the other studies was extracted for meta-analysis. For example, one study investigated a ‘basic app’ (which provided a uni-directional once daily reminder) as well as an ‘advanced app’ (which provided customisable and interactive features) versus usual care.[16] The reviewers agreed that the advanced app was most comparable to the interventions of the other studies and so data pertaining to this intervention was extracted. BCTs that were utilised in the app-based interventions were coded according to the Behaviour Change Taxonomy.[17] This is a structured taxonomy of

For peer review only - http://bmjopen.bmj.com/site/about/guidelines.xhtml

techniques utilised by interventions to change behaviour. The BCT Taxonomy does not have a code relating to performance of behaviour and therefore where participants were required to enter whether or not medication was taken, this was coded as 'Report whether or not the behaviour was performed'. Similarly, the Taxonomy does not have a code relating to tailoring of an intervention and therefore where a study used information gained about a person to define the content, frequency or format of an intervention, this was coded as a 'Tailored' intervention.[18]

The included trials were assessed for risk of bias using the Cochrane Risk of Bias Tool.[19] Two reviewers (LA and AK) individually assessed each of the included articles against criteria outlined in the tool. Any discrepancies at any stage in the coding process were settled through discussion between the three reviewers (LA, AK, SS). Studies were considered at unclear risk of bias for an item when the primary article did not provide sufficient information for the reviewers to decide categorically between high or low risk of bias.

Statistical Analysis

Outcome data on medication adherence was extracted from each of the included studies at the final observation point. Authors were contacted for raw final observation point adherence data if this was not reported in published manuscripts. Trials were considered homogenous based on the content of the intervention and comparator groups and the outcome measurement of medication adherence. Comprehensive Meta-Analysis software[20] was used to investigate the overall effect of app-delivered interventions on medication adherence using a random-effects model. The same software was used to perform meta-regression using the behaviour change techniques utilised by the applications. Comprehensive Meta-analysis Software conversion function was used where required to convert extracted raw data into odds ratio and standardised mean difference between the intervention and control groups was used.[21] The I^2 calculation was used to estimate heterogeneity between included studies.

For peer review only - http://bmjopen.bmj.com/site/about/guidelines.xhtml

RESULTS

The initial electronic database search identified 13 259 potentially eligible studies and two were identified from reference lists of identified articles. After removal of duplicates 9971 citations were screened by title and abstract. We considered 83 of these to be eligible and retrieved the full-text articles. Of these, nine studies reported outcome data eligible for meta-analysis[12,13,15,16,22–26]. The screening process and reasons for exclusion are reported in Figure 1.

Study characteristics of the nine studies are displayed in Table 1. Publication year of included studies ranged from 2014-2018. Mean age in the intervention groups ranged from 20.3 (SD 4.0)[22] to 73.8 (SD 8).[12] One did not report the mean age but presented the percentage of participants whose age fell within a series of bandwidths.[15] The percentage of participants in intervention groups who were female ranged from 11%[16] to 89%.[22]

Health problem of the target population varied between studies. Five studies sampled a patient population with cardiovascular disease[12,13,16,23,26] and each of the remaining four studies enrolled patient populations with different health problems: depression[22], Parkinson's disease,[24] psoriasis[15] and multimorbidity.[25]

Outcome Measurement Methods

A number of measures of medication adherence were utilised by the included studies. Of the 9 studies, four measured adherence using the 8 Item Morisky Medication Adherence Scale[24,26,27], with one of these also measuring adherence through a medication possession ratio (see Supplementary Material file, Shah et al, 2016).[13] For this study, the Morisky Medication Adherence Scale data was extracted for the meta-analysis as this was most comparable to the data available for other studies. A further study measured the 4 Item Morisky Medication Adherence

For peer review only - http://bmjopen.bmj.com/site/about/guidelines.xhtml

Scale.[25] Of the remaining studies, two reported adherence according to pill count[22,23], one the A14 self-report scale of medication adherence[12] and one through a digital chip record fitted within the medication dispenser (Table 1).[15]

Meta-analysis

Meta-analysis was performed, pooling results from the nine eligible studies which reported data on 988 participants, to estimate the effect of mobile applications to improve medication adherence.[12,13,15,16,22–26] The result of this meta-analysis indicates that patients who participate in medication adherence interventions delivered by mobile applications are more likely to adhere to prescribed medications (OR 2.120, 95% CI 1.635-2.747) than those that do not use such interventions. The I^2 value was 9.8 indicating low statistical heterogeneity between the studies included in the meta-analysis. The forest plot of the meta-analysis can be seen in Figure 2. The weighting of each individual study in the meta-analysis can be seen in eTable 1 and raw data extracted for the meta-analysis can be found in eTable 2.

A focussed meta-analysis was performed, pooling results from the 5 studies which reported medication adherence according to the Morisky Medication Adherence Scale.[13,16,24–26] The result of this also indicated that patients using medication adherence interventions delivered by mobile applications report higher adherence to prescribed medications (OR 1.83, 95% CI 1.42-2.36).

Behaviour Change Techniques

The most common BCT utilised by applications was 'Tailored", which was utilised by all studies. The next most common was 'Prompts and Cues', utilised by seven studies[12,15,16,22–25] and 'Report whether or not the behaviour was performed' which was utilised by six studies.[12,16,22–25] Information on the BCTs coded for each of the interventions and definitions of each of the BCTs is available in eTables 3 and 4. Agreement between reviewers for classification of Behaviour Change Techniques utilised by the interventions was assessed using the kappa statistic. This revealed a 'very

For peer review only - http://bmjopen.bmj.com/site/about/guidelines.xhtml

good' level of agreement (kappa = 0.902, 95% CI 0.835 – 0.969). All behaviour change techniques coded for each of the study interventions are provided in eTable3. Those coded in more than three but less than 6 of the 9 studies were included in the meta-regression; this did not reveal any significant associations between the behaviour change techniques utilised and effect size.

Risk of bias at primary study level

A summary of the risk of bias analysis can be seen in Table 2**.** Overall, all studies were deemed as unclear in their risk of bias.

The most common source of risk of bias amongst the studies was the absence of blinding of participants and personnel to the outcome measure. The second most common risk of bias arose from a lack of objective measurements of medication adherence. This was due to the use of self-report measures of adherence: six of the studies included in the meta-analysis reported adherence based on a self-assessment scale[12,13,16,24–26], and one study each used patient-entered medication logs in an app[22], an electronically chipped medicated foam dispenser[15] and pill counts at clinic visits.[23]

Self-assessment scales are a form of self-report and therefore are a source of potential bias, including those of social desirability, and those arising from selective recall and the duration of the recall period.[28] Two studies included in this review, which measured adherence through participant self-report, sought to compare their subjective measurements of adherence with serum measurements of either drug-reactivity units or a medication's anticoagulant effect.[13,23] One study showed that serum measurement of the medication's anticoagulant effect was similar between both the intervention and comparator groups, despite adherence measures being better in the intervention group.[23] The other study revealed that drug-reactivity units were worse in the intervention group than the control group, which conflicted with measured adherence outcome

For peer review only - http://bmjopen.bmj.com/site/about/guidelines.xhtml

data.[13] However, the presence of a drug biomarker does not equate to compliance and the absence does not equate to non-compliance.[29] Indeed, drug metabolism should be taken into account for such measures of medication adherence, as physiological state and metabolic rate vary among individuals.[30]

Publication Bias

A funnel plot of effect size estimates of each of the studies included in the meta-analysis is provided in the online supplementary file (eFigure 1). Formal analysis of plot symmetry was not performed owing to the total number of studies included being less than 10.[31] Visual inspection reveals moderate symmetry of the plot.

DISCUSSION

Principle Findings

We conducted a rigorous systematic review of the literature to investigate whether mobile device applications are effective at supporting medication adherence. A random-effects meta-analysis of the pooled results from all nine studies indicated that people who use medication reminder apps are significantly more likely to adhere to their medications than those who do not, but the results should be interpreted with caution owing data for 6 of the 9 studies included in the meta-analysis being self-reported measures of adherence.

Intervention effects reported between the studies varied, as shown in the forest plot (Figure 2). All nine studies reported that their mobile app-delivered interventions were effective at supporting medication adherence although in five studies the interventions did not have a statistically significant effect.[12,13,16,22,25] Although the I^2 measure of statistical heterogeneity was low, and meta-regression of the BCTs utilised by the studies was not able to account for variance in effect observed between studies, there are other potential sources of methodological and clinical heterogeneity which may account for this. One potential explanation might be the variation in

For peer review only

For peer review only - http://bmjopen.bmj.com/site/about/guidelines.xhtml

adherence measurement. This may be illustrated by three studies which incorporated five of the same BCTs[23–25] but where one[23] reported better medication adherence (OR 10.633, 95% CI 2.339-48.344) than the other two (OR 1.946, 95% CI 1.094-3.461 and OR 1.251, 95% CI 0.612-2.559).[24,25] In Labovitz et al's study, the medication adherence was measured by pill counts at clinic follow up visits[23], whereas in the other two studies, adherence was measured by retrospective self-scoring on a medication adherence scale[24,25] It could be argued that both of these different measures of adherence are vulnerable to bias with pill counts potentially being vulnerable to expectation bias and retrospective self-scores being vulnerable to recall bias. We subsequently performed a focused meta-analysis of the five studies which measured adherence using the Morisky Medication Adherence Scale to investigate whether this influenced the effect size observed. Whilst the result of meta-analysis remained significant, the effect size was lower. This may support the use of real-time or prospective adherence monitoring, over retrospective self-report for future research in the field of medication adherence.

A further possible explanation for variance in effect size might be techniques, beyond those reported in the behaviour change techniques taxonomy, utilised by the apps. For example, in the study by Labovitz et al, the application intervention employed artificial intelligence to identify the participant, their medication and ingestion of the medication, using the camera of the mobile device and software algorithms. This could be regarded as a highly tailored intervention and one might hypothesise that the degree of tailoring could influence the effect size observed. A further example of a highly tailored intervention was seen in the study by Lakshminarayana et al. All of the app-based interventions in the included studies were tailored to the prescribed medication regimen, but in this instance the tailoring went beyond the prescribed medication regimen, to include tailoring to the patients' intentional and non-intentional non-adherence, beliefs about medication taking, mood, cognitive impairment, symptom control, quality of life, age and disease duration. A significant effect

For peer review only - http://bmjopen.bmj.com/site/about/guidelines.xhtml

size was observed in this study and this may support a hypothesis that highly tailored apps are more effective at supporting medication adherence, than minimally tailored apps.

Further factors which may have contributed to variance in the effect size seen between studies relate to the participants, including disease burden, non-adherence behaviours, perceived benefit from the medication and any side effects. Interpretation of the overall result of the meta-analysis and generalisability to the wider population should therefore be cautious.

Strengths and Weaknesses of the Primary Studies

Based on the assessment of the risk of bias undertaken as part of this review, the evidence included in this meta-analysis is of moderate quality. The most common weaknesses in this evidence are small sample size, lack of objectivity of the outcome measurement, a lack of blinding of participants and personnel and limited follow up time.

Strengths and Weaknesses at Review Level

The pooling of data from a group of studies which used a variety of methods for measurement of medication adherence, targeted at patients with different healthcare problems is a limitation of this review. As further studies in this field are published it will become more feasible to conduct subgroup analysis or meta-regression analyses against variables such as age, health condition, targeted medication, intervention content and application characteristics. A standardised protocol for measuring adherence and reporting the content and characteristics of application interventions may facilitate further analysis of the latter two variables.

This review cannot report on the likely sustainability of the effect on medication adherence given the short study duration of the studies included in this review; across the 9 studies, the maximum follow up period was 16 weeks. Furthermore, the optimal frequency and duration of application use that is required to achieve improvements in medication adherence is not known. Long term and

For peer review only
For peer review only - http://bmjopen.bmj.com/site/about/guidelines.xhtml

large-scale research is required in this field to addresses the question regarding sustainability of the observed effect found in this meta-analysis.

This systematic review and meta-analysis included studies with a range of ages of participants. The lowest mean age of participants in the included studies was 20.9 years and the highest was 70.9 years. The ubiquity and use of mobile devices varies across age groups[7] and whilst the majority of participants in these studies were aged 50 and over, further research to establish the acceptability and usability of adherence applications among different age groups adults would be valuable.

Finally, the reviewers set out to perform a meta-regression of the BCTs utilised by application interventions in the included studies.[11] However, this did not reveal any significant association between the BCTs utilised and observed effect size. This may be owing to the low estimate of heterogeneity and the small number of eligible included studies.

Conclusions

This meta-analysis indicates that medication adherence interventions delivered by smartphone applications can be effective at improving patients' adherence to their prescribed medications. However, when considering whether to recommend an application to patients, these results should be interpreted with caution owing to variance in effect size seen between the studies, the small sample sizes taken from different patient populations, heterogeneity among included studies and the prominence of self-reported scales to measure medication adherence. Further research should aim to first establish a standard protocol for the measuring and reporting of adherence in the context of evaluating digital interventions. Subsequently, further research investigating intervention characteristics associated with an application's effect and the sustainability of any effect gained would add to this field.

.

For peer review only - http://bmjopen.bmj.com/site/about/guidelines.xhtml

References

1 Osterberg L, Blaschke T. Adherence to Medication. *N Engl J Med* 2005;**353**:487–97. doi:10.1056/NEJMra050100

2 Brown MT, Bussell JK. Medication Adherence: WHO Cares? *Mayo Clin Proc* 2011;**86**:304. doi:10.4065/MCP.2010.0575

3 Elliott RA, Boyd MJ, Waring J, *et al.* Understanding & evaluating the New Medicines Service in the NHS in England (029/0124). 2014. http://www.nottingham.ac.uk/~pazmjb/nms/downloads/report/files/assets/common/downloads/108842 A4 Main Report.v4.pdf (accessed 12 Feb 2018).

4 Iuga AO, McGuire MJ. Adherence and health care costs. *Risk Manag Healthc Policy* 2014;**7**:35–44. doi:10.2147/RMHP.S19801

5 Sabaté E. Evidence for action. *World Heal Organ* Published Online First: 2003.http://www.who.int/chp/knowledge/publications/adherence_introduction.pdf (accessed 12 Feb 2018).

6 Ofcom. Communications Market Report. 2018. https://www.ofcom.org.uk/__data/assets/pdf_file/0022/117256/CMR-2018-narrative-report.pdf (accessed 3 Aug 2018).

7 Ofcom. The Communications Market Report. 2015. https://www.ofcom.org.uk/__data/assets/pdf_file/0022/20668/cmr_uk_2015.pdf (accessed 12 Feb 2018).

8 Ahmed I, Ahmad NS, Ali S, *et al.* Medication Adherence Apps: Review and Content Analysis. *JMIR mHealth uHealth* 2018;**6**:e62. doi:10.2196/mhealth.6432

For peer review only - http://bmjopen.bmj.com/site/about/guidelines.xhtml

9 Wideman TH, Sullivan MJL, Inada S, *et al.* Behaviour Change Techniques. In: Gellman Marc D., Turner J. Rick., eds. *Encyclopedia of Behavioral Medicine*. New York, NY: : Springer New York 2013. 12. doi:10.1007/978-1-4419-1005-9_1661

10 Moher D, Liberati A, Tetzlaff J, *et al.* Preferred Reporting Items for Systematic Reviews and Meta-Analyses: The PRISMA Statement. *PLoS Med* 2009;**6**:e1000097. doi:10.1371/journal.pmed.1000097

11 Armitage L, Kassavou A, Sutton S. A systematic review of the literature with met-analysis and meta-regression: the use of smartphone applications to support medication adherence/. PROSPERO Int. Prospect. Regist. Syst. Rev. 2017. doi:CRD42017080150

12 Mertens A, Brandl C, Miron-shatz T, *et al.* A mobile application improves therapy-adherence rates in elderly patients undergoing rehabilitation. *Medicine (Baltimore)* 2016;**95**:e4446.

13 Shah V, Dileep A, Dickens C, *et al.* Patient-Centered Tablet Application for Improving Medication Adherence after a Drug-Eluting Stent. *Front public Heal* 2016;**4**:272. doi:10.3389/fpubh.2016.00272

14 DeVito Dabbs A, Song MK, Myers BA, *et al.* A Randomized Controlled Trial of a Mobile Health Intervention to Promote Self-Management After Lung Transplantation. *Am J Transplant* 2016;**16**:2172–80. doi:10.1111/ajt.13701

15 Svendsen MT, Andersen F, Andersen KH, *et al.* A smartphone application supporting patients with psoriasis improves adherence to topical treatment: a randomized controlled trial. *Br J Dermatol* 2018;**179**:1062–71. doi:10.1111/bjd.16667

16 Santo K, Singleton A, Rogers K, *et al.* Medication reminder applications to improve adherence in coronary heart disease: a randomised clinical trial. *Heart* 2018;:heartjnl-2018-313479. doi:10.1136/heartjnl-2018-313479

For peer review only - http://bmjopen.bmj.com/site/about/guidelines.xhtml

17 Michie S, Richardson M, Johnston M, *et al.* The Behavior Change Technique Taxonomy (v1) of 93 Hierarchically Clustered Techniques: Building an International Consensus for the Reporting of Behavior Change Interventions. *Ann Behav Med* 2013;**46**:81–95. doi:10.1007/s12160-013-9486-6

18 Kassavou A, Sutton S. Automated telecommunication interventions to promote adherence to cardio-metabolic medications: meta-analysis of effectiveness and meta-regression of behaviour change techniques. *Health Psychol Rev* 2018;**12**:25–42. doi:10.1080/17437199.2017.1365617

19 Higgins JP, Altman DG, Sterne JA. Chapter 8: Assessing risk of bias in included studies. https://training.cochrane.org/handbook/chapter-08 (accessed 14 Feb 2018).

20 Comprehensive Meta-Analysis Software (CMA). https://www.meta-analysis.com/ (accessed 15 Feb 2018).

21 Borenstein M, Hedges L., Higgins PT, *et al. Introduction to Meta-Analysis, Converting Among Effect Sizes*. John Wiley and Sons 2009. https://www.meta-analysis.com/downloads/Meta-analysis Converting among effect sizes.pdf (accessed 8 Mar 2019).

22 Hammonds T, Rickert K, Goldstein C, *et al.* Adherence to Antidepressant Medications: A Randomized Controlled Trial of Medication Reminding in College Students. 2015;**8481**. doi:10.1080/07448481.2014.975716

23 Labovitz DL, Shafner L, Gil MR, *et al.* Using Artificial Intelligence to Reduce the Risk of Nonadherence in Patients on Anticoagulation Therapy. *Stroke* 2017;**48**:1416–9. doi:10.1161/STROKEAHA.116.016281

24 Lakshminarayana R, Wang D, Burn D, *et al.* Using a smartphone-based self-management platform to support medication adherence and clinical consultation in Parkinson ' s disease.

For peer review only - http://bmjopen.bmj.com/site/about/guidelines.xhtml

NPJ Park Dis 2017;**3**:1–8. doi:10.1038/s41531-016-0003-z

25 Mira JJ, Navarro I, Botella F, *et al.* A Spanish pillbox app for elderly patients taking multiple medications: randomized controlled trial. *J Med Internet Res* 2014;**16**:e99. doi:10.2196/jmir.3269

26 Morawski K, Ghazinouri R, Krumme A, *et al.* Association of a Smartphone Application With Medication Adherence and Blood Pressure Control. *JAMA Intern Med* 2018;**178**:802. doi:10.1001/jamainternmed.2018.0447

27 Santo K, Chow CK, Thiagalingam A, *et al.* MEDication reminder APPs to improve medication adherence in Coronary Heart Disease (MedApp-CHD) Study : a randomised controlled trial protocol. 2017;:1–10. doi:10.1136/bmjopen-2017-017540

28 Althubaiti A. Information bias in health research: definition, pitfalls, and adjustment methods. *J Multidiscip Healthc* 2016;**9**:211–7. doi:10.2147/JMDH.S104807

29 Farmer KC. Methods for measuring and monitoring medication regimen adherence in clinical trials and clinical practice. *Clin Ther* 1999;**21**:1074–90. doi:10.1016/S0149-2918(99)80026-5

30 Lam WY, Fresco P. Medication Adherence Measures: An Overview. *Biomed Res Int* 2015;**2015**:217047. doi:10.1155/2015/217047

31 Sterne JAC, Sutton AJ, Ioannidis JPA, *et al.* Recommendations for examining and interpreting funnel plot asymmetry in meta-analyses of randomised controlled trials. *BMJ* 2011;**343**:d4002. doi:10.1136/BMJ.D4002

Sources of funding

For peer review only - http://bmjopen.bmj.com/site/about/guidelines.xhtml

This paper presents independent research funded by the National Institute for Health Research (NIHR) under its Programme Grants for Applied Research Programme (Grant Reference Number RP-PG-0615-20013). The views expressed are those of the authors and not necessarily those of the NIHR or the Department of Health and Social Care. The National Institute for Health Research's (NIHR) Collaboration for Leadership in Applied Health Research and Care (CLAHRC) in East of England provided salary support to LA during conduction of this review. The funders had no role in review design, data collection, data analysis, data interpretation, the writing of the manuscript, and decision to submit the manuscript for publication.

Author Contributions

All authors were involved in design of the review and undertook methodological planning. LA performed and refined the searches in consultation with a medical librarian. LA and KK performed initial screening and data extraction and SS gave screening advice where any disagreements arose. All authors contributed to performing the meta-analysis to data interpretation. LA led the writing and all authors contributed to successive drafts and approved the final manuscript.

Data Availability

All data relevant to the study are included in the article or uploaded as supplementary information

Competing Interests

The authors have no competing interests to declare

Figure Legends

Figure 1: PRISMA Flowchart

Figure 2. Forest Plot of Randomised Trials evaluating a mobile device application to support medication adherence against usual care.

For peer review only - http://bmjopen.bmj.com/site/about/guidelines.xhtml

For peer review only

For peer review only - http://bmjopen.bmj.com/site/about/guidelines.xhtml

Table 1: Study Characteristics

Source (Sample size)	Study Nation	Study Design	Health Problem of Target Population	Recruitment setting	Participant characteristics at recruitment				Application intervention	Outcome measure	Follow up interval
					Intervention group		Comparator group				
					Mean age (SD)	Female, N (%)	Mean age (SD)	Female, N (%)			
Hammonds et al, 2015 (57)	USA	RCT	Depression	College students were recruited via campus based advertisements and the university research recruitment system	20.3 (4.0)	25 (83)	20.9 (4.7)	24 (89)	Medication reminder app	No. of participants taken ≥80% prescribed tablets per day.	25-35d
Labovitz et al, 2017 (28)	USA	RCT	Ischaemic stroke requiring secondary oral anti-coagulation	Outpatient clinic	58.3 (9.79)	6 (40)	55.5 (16.55)	9 (69)	Artificial intelligence app	Pill count	12w
Lakshmin-arayana et al, 2017 (215)	UK	RCT	Parkinson's disease	outpatient clinic	59.86 (9.13)	34 (36.2)	60.71 (10.26)	45 (42.1)	Parkinson's tracker app	Self-report: 8 Item MMAS	16w
Mertens et al, 2016 (24)	Germany	RCT Cross-over	Cardiovascular disease	Cardiac rehab groups	73.8[a]	12 (50)[a]	73.8[a]	12 (50)[a]	Medication Plan app (version 1.3)	Self-report: A14 scale	28d
Mira et al, 2014 (102)	Spain	RCT	Multi-comorbidity	Community health centres	70.9 (8)	21 (41)	72.9 (6)	23 (48)	ALICE medication self-management app	Self-report: 4 Item MMAS	3m

For peer review only - http://bmjopen.bmj.com/site/about/guidelines.xhtml

Morawski et al, 2018<br><br>(412)	USA	RCT	Hypertension	Social media, mobile apps, targeted advertisements and online communications	51.7 (10.5)	120 (57.4)	52.4 (10.1)	127 (62.9)	MediSAFE-BP app	Self-report: 8 Item MMAS	12w
Santo et al, 2018<br><br>(163)	Australia	RCT	Coronary Heart Disease	Inpatient, outpatient and rehabilitative care settings.	58.4 (9.04)	14 (13)	56.8 (8.64)	6 (11)	MedApp-CHD	Self-report: 8 Item MMAS	3m
Shah et al, 2016<br><br>(24)	USA	RCT	Cardiovascular stent	Patients in the immediate post-operative phase of insertion of a drug-eluting cardiovascular stent	60.5 (8.9)	6 (46)	60.6 (4.1)	4 (36)	MyIDEA education app	Medication possession ratio and Self-report: 8 Item MMAS	90d
Svendsen et al, 2018<br><br>(134)	Denmark	RCT	Psoriasis	Hospital posters and the dermatology outpatient clinic.	18-40 yrs 29[b]<br>41-50 21<br>51-60 26<br>61-75 24	27 (40)	18-40 yrs 32[b]<br>41-50 20<br>51-60 24<br>61-75 24	25 (38)	My Pso SmarTop Version 1.0 or Smartop number 0536776	Measured via a digital chip record in the medication dispenser.	28d

MMAS = Morisky Medication Adherence Scale.

[a]Baseline characteristics presented for whole cohort as study design was a randomised cross-over trial; [b]number of participants in each age group presented as mean age not available.

For peer review only

For peer review only - http://bmjopen.bmj.com/site/about/guidelines.xhtml

Table 2: Risk of Bias Summary Table

Source	Allocation sequence adequately:		Free from baseline imbalance?			Blinding of participants and personnel and	Objective outcome assessment	Incomplete outcome data adequately addressed	Free from selective outcome reporting	Was the intervention independent of other bias
	Generated	Concealed	Age	Gender	Adherence					
Hammonds et al, 2015	🟡	🟡	🟢	🟢	🟢	🔴	🔴	🟢	🟢	🟢
Labovitz et al, 2017	🟡	🟡	🟡	🟡	🟡	🔴	🔴	🟢	🟢	🟢
Lakshminarayana et al, 2017	🟢	🟢	🟡	🟢	🟢	🔴	🟡	🟢	🟡	🟢
Mertens et al, 2016	🟡	🟡	🟡	🟡	🟡	🔴	🔴	🟢	🔴	🟡
Mira et al, 2014	🟡	🟡	🟡	🟢	🟢	🔴	🔴	🟡	🟢	🟢
Morawski et al, 2018	🟢	🟡	🟡	🟢	🟢	🟡	🔴	🟡	🟢	🟢
Santo et al, 2018	🟢	🟡	🟡	🟢	🟢	🔴	🔴	🟡	🟢	🟢
Shah et al, 2016	🟢	🟢	🟡	🟡	🟡	🟡	🟡	🟢	🟢	🟢
Svendsen et al, 2018	🟢	🟢	🟡	🟡	🟡	🔴	🟢	🟢	🟢	🟢

🟢 = Low risk of bias, 🟡 = Unclear risk of bias, 🔴 = High risk of bias

For peer review only

For peer review only - http://bmjopen.bmj.com/site/about/guidelines.xhtml

For peer review only

For peer review only - http://bmjopen.bmj.com/site/about/guidelines.xhtml

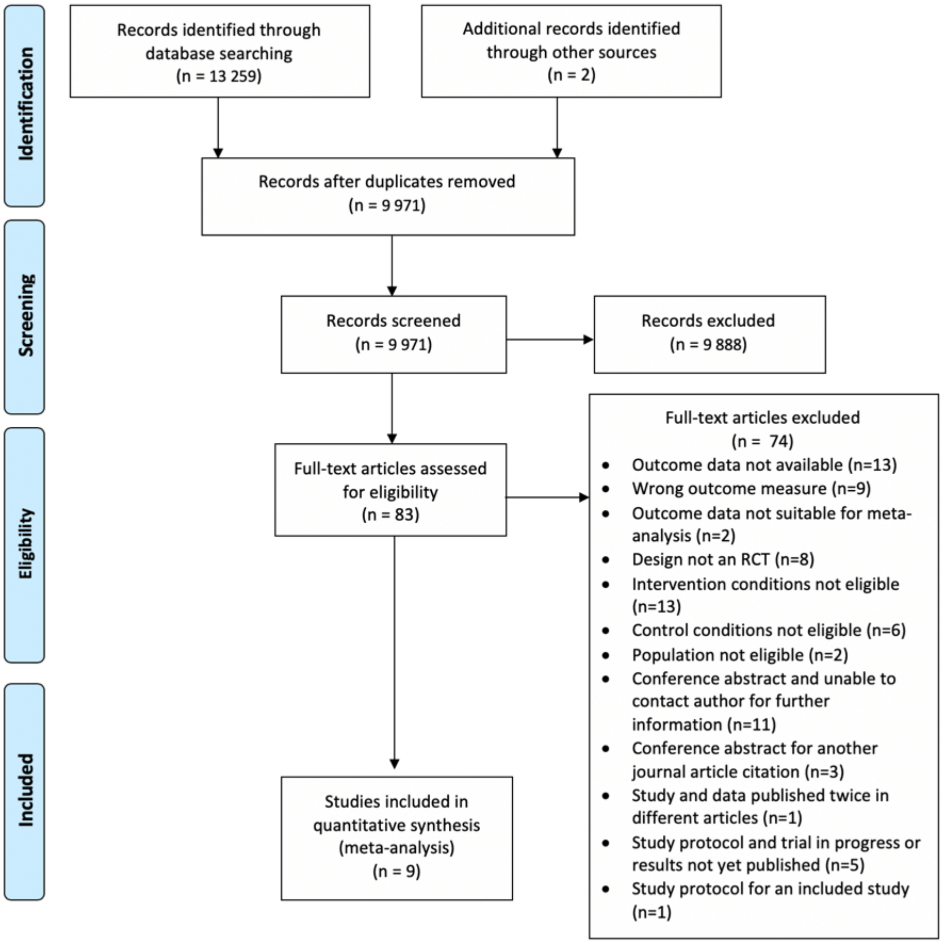

Figure 1: PRISMA Flowchart

Study name	Statistics for each study				
	Odds ratio	Lower limit	Upper limit	Z-Value	p-Value
Hammonds 2015	3.500	0.945	12.966	1.875	0.061
Labovitz 2017	10.633	2.339	48.344	3.060	0.002
Lakshminarayana 2017	1.946	1.094	3.461	2.266	0.023
Mertens 2016	2.975	0.675	13.117	1.440	0.150
Mira 2014	1.251	0.612	2.559	0.614	0.539
Morawski 2018	1.896	1.331	2.700	3.547	0.000
Shah 2016	2.206	0.505	9.626	1.052	0.293
Svendsen 2018	3.222	1.526	6.803	3.068	0.002
Santo 2018	2.029	0.991	4.154	1.934	0.053
	2.120	1.635	2.747	5.677	0.000

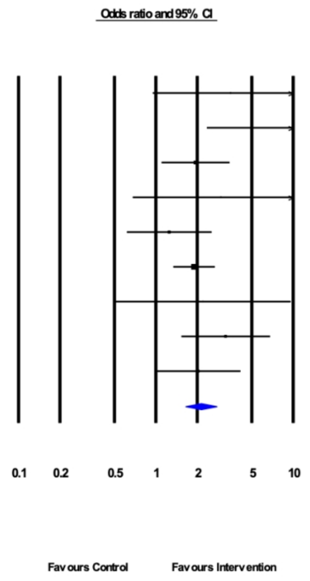

Figure 2. Forest Plot of Randomised Trials evaluating a mobile device application to support medication adherence against usual care.

SUPPLEMENTAL MATERIAL FILE

Mobile device applications to support medication adherence: A systematic review of randomised controlled trials, with meta-analysis

Author Details:

Laura Armitage, MRCGP

Aikaterini Kassavou, PhD

Stephen Sutton, PhD

For peer review only

For peer review only - http://bmjopen.bmj.com/site/about/guidelines.xhtml

eText 1: Medline/Pubmed Search Strategy

1. medication adherence.mp. or exp Patient Compliance/ or exp Medication Adherence/
2. nonnadherence.mp.
3. non-adherence.mp.
4. non adherence.mp.
5. concordance.mp.
6. concordant.mp.
7. 1 or 2 or 3 or 4 or 5 or 6
8. app.mp.
9. apps.mp.
10. application.mp.
11. exp Mobile Applications/ or mobile application*.mp.
12. smartphone.mp. or exp Smartphone/ or exp Cell Phones/
13. mobile*.mp.
14. phone*.mp.
15. 8 or 9 or 10 or 11 or 12 or 13 or 14
16. randomized controlled trial.mp. or exp Randomized Controlled Trial/
17. RCT.mp.
18. randomised.mp.
19. randomized.mp.
20. control*.mp.
21. 16 or 17 or 18 or 19 or 20
22. 7 and 15 and 21
23. limit 22 to (english language and yr="1990 - 2017")

For peer review only - http://bmjopen.bmj.com/site/about/guidelines.xhtml

eFigure 1: Funnel Plot of effect estimates from studies included in meta-analysis

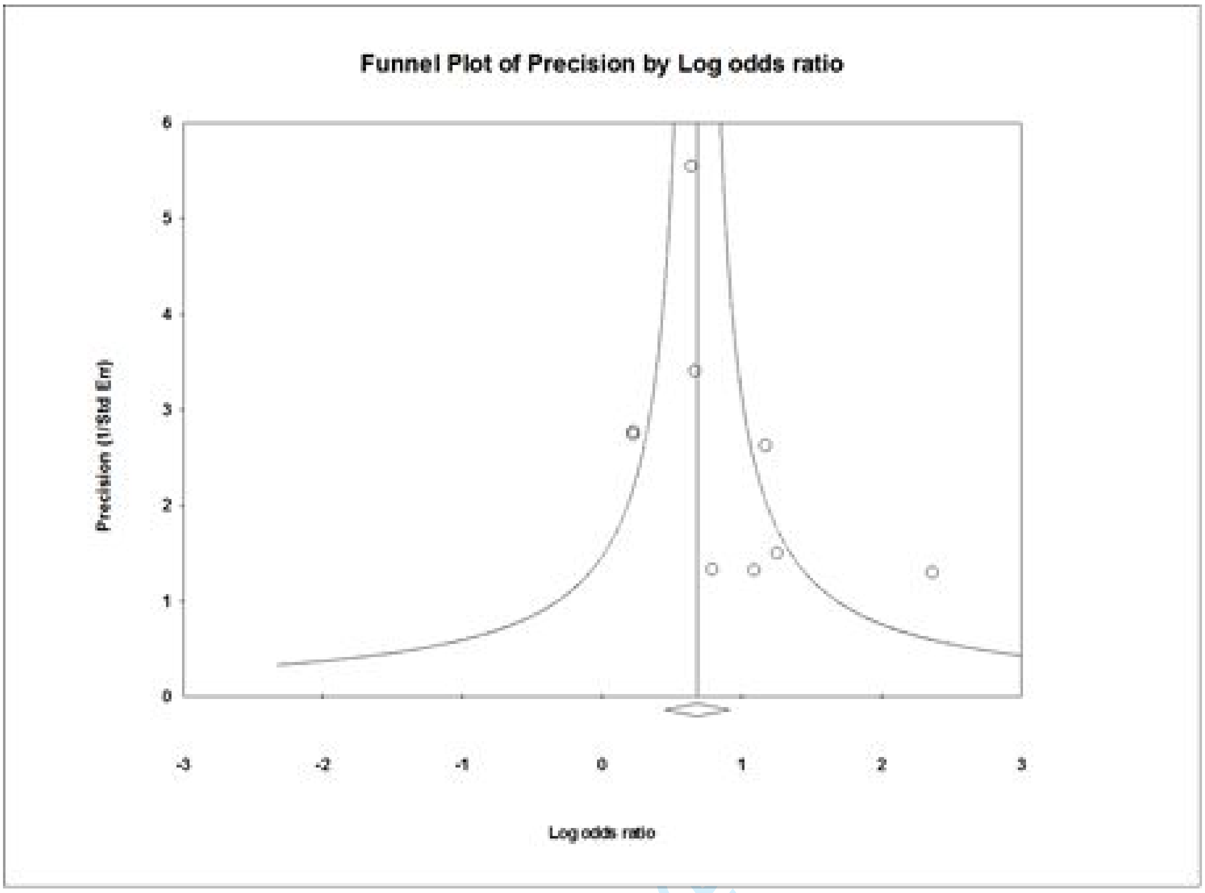

For peer review only - http://bmjopen.bmj.com/site/about/guidelines.xhtml

eTable 1: Relative weighting of each study included in the 9 study meta-analysis

Study	Weight (random)	Relative weight
Hammonds et al, 2015	2.16	3.79
Labovitz et al, 2017	1.63	2.86
Lakshminarayana et al, 2017	9.76	17.10
Mertens et al, 2018	1.70	2.97
Mira et al, 2014	6.69	11.72
Morawski et al, 2018	20.58	36.03
Santo et al, 2018	6.68	11.69
Shah et al, 2016	1.72	3.01
Svendsen et al, 2018	6.19	10.84

For peer review only

eTable 2: Raw data extracted for each study for meta-analysis

Study	Adherence measurement	Intervention group				Comparator group			
		Mean (SD)	N	Events	Totals	Mean (SD)	N	Events	Totals
Hammonds et al, 2015	Number of participants taken ≥80% prescribed tablets per day	-	-	12	20	-	-	6	20
Labovitz et al, 2017	Mean cumulative adherence, measured by pill count	97.2 (4.4)	15	-	-	90.6 (5.8)	12	-	-
Lakshminaray-ana et al, 2017	MMAS-8	6.3 (1.52)	68	-	-	5.74 (1.53)	90	-	-
Mertens et al, 2018	A14 Scale	53.96 (2.01)	12	-	-	52.6 (2.49)	12	-	-
Mira et al, 2014	MMAS-4	7.4 (0.9)	51	-	-	7.3 (0.7)	48	-	-
Morawski et al, 2018	MMAS-8	6.3 (1.6)	209	-	-	5.7 (1.8)	202	-	-
Santo et al, 2018	MMAS-8	7.02 (n/a)	50	-	-	6.63 (n/a)	51	-	-
Shah et al, 2016	MMAS-8	2.2 (1.2)	5	-	-	2.1 (1)	3	-	-
Svendsen et al, 2018	Number of patients having applied medication ≥80% days in treatment period	-	-	39	59	-	-	23	61

A14 = A14 Medication Adherence Scale; MMAS-8 = 8 Item Morisky Medication Adherence Scale; MMAS 4 = 4 Item Morisky Medication Adherence Scale

For peer review only - http://bmjopen.bmj.com/site/about/guidelines.xhtml

eTable 3: Behaviour Change Techniques utilised by in applications in the included studies.

Study	BCTs Coded							
	Tailored	Prompts and cues	Report whether behaviour was performed	Monitoring of behaviour by others without feedback	Feedback on behaviour	Social support (unspecified)	Habit formation	Goal setting of behaviour
Hammonds et al, 2015	+	+	+	-	-	-	-	-
Labovitz et al, 2017	+	+	+	+	+	-	-	-
Lakshminarayana et al, 2017	+	+	+	+	+	-	-	-
Mertens et al, 2016	+	+	+	-	-	-	-	-
Mira et al, 2014	+	+	+	+	+	-	+	+
Morawski et al, 2018	+	-	+	-	+	+	+	+
Santo et al, 2018	+	-	+	-	+	+	+	-
Shah et al, 2016	+	-	-	-	-	+	-	-
Svendsen et al, 2018	+	-	-	-	+	-	-	-

Those Behaviour Change Techniques coded in 3 but not more than 6 studies were included for meta-regression.

For peer review only - http://bmjopen.bmj.com/site/about/guidelines.xhtml

eTable 4: Definitions of Behaviour Change Techniques coded as present in applications of the included studies and examples of each.

**Behaviour Change Technique**	**Definition**	**Examples**
Tailored	Use information gained about the person to define the content, frequency or format of an intervention directed to the behaviour.	Shah et al, 2016: information about the participant including their symptoms and coronary artery catheterising procedure were recorded and used to tailor the information delivered to the participant via the MyIDEA application.[1]<br><br>Mira et al, 2014: The ALICE app delivered a customised system of alerts and reminders, according to their personalised prescription to remind participants when to take their medication.[2]
Prompts and Cues	*Introduce or define environmental or social stimulus with the purpose of prompting or cueing the behaviour. The prompt and cue would normally occur at the time or place of performance.*[3]	Mira et al, 2014: The ALICE app delivered a customised system of alerts and reminders, according to their personalised prescription to remind participants when to take their medication.[2]<br><br>Lakshminarayana et al, 2017: The app included a reminder system for participants to set up and allow medication alerts.[4]
Report whether or not behaviour was performed	Ask a person to report when they have performed a behaviour without the requirement of monitoring or recording the behaviour.[5]	Santo et al, 2018: Reminders from the application could be snoozed, rescheduled and/or marked as 'taken' or 'missed dose'.[6]<br><br>Hammonds et al, 2015: Participants indicated when they had taken their medication by responding to the alert message.[7]

For peer review only - http://bmjopen.bmj.com/site/about/guidelines.xhtml

Monitoring of behaviour by others without feedback	*Observe or record behaviour with the person's knowledge as part of a behaviour change strategy.*[3]	Labovitz et al, 2017: Clinic staff received automated text messages or emails if doses were missed, late or based on incorrect usage.[8]<br><br>Lakshminarayana et al, 2017: Clinicians had access to a dedicated web portal where they could see the participants' progress over the trial period.[9]
Feedback on behaviour	*Monitor and provide informative or evaluative feedback on performance of the behaviour (e.g. form, frequency, duration, intensity.*[3]	Mira et al, 2014: The ALICE app reported to the participant medication that they had taken that day and those that the participant had not taken.[2]<br><br>Santo et al, 2018: The app included a feature of medication adherence statistics.[10]
Social support (unspecified)	*Advise on, arrange or provide social support (e.g. from friends, relatives, colleagues, buddies or staff) or non-contingent praise or reward for performance of the behaviour. It includes encouragement and counselling, but only when it is directed at the behaviour.*[3]	Morawski et al, 2018: Users were able to designate a "Medfriend" who was granted access to the person's medication taking history and received alerts when doses were missed and could provide peer support.[11]<br><br>Santo et al, 2018: The app included the ability to export and share information with others such as family members and the ability to alert them if the participant missed a dose.[10]
Habit formation	*Prompt rehearsal and repetition of the behaviour in the same context repeatedly so that the context elicits the behaviour.*[3]	Mira et al 2014: alerts and reminders encouraged participants to put healthy habits into practice, e.g. the association of taking their medicines with particular meals or daily activities.[2]<br><br>Santo et al, 2018: Scheduled reminders occurred up to three times at 10 minute intervals.[10]

For peer review only - http://bmjopen.bmj.com/site/about/guidelines.xhtml

Goal setting of behaviour	*Set or agree a goal defined in terms of the behaviour to be achieved.*[3]	Mira et al, 2014: The ALICE app included a function for the participant to store details of their prescriptions and related instructions.[2]<br><br>Morawski et al, 2018: Users were able to enter their medication lists manually, along with their preferred timing of administration.[11]

For peer review only

References

1 Shah V, Dileep A, Dickens C, *et al.* Patient-Centered Tablet Application for Improving Medication Adherence after a Drug-Eluting Stent. *Front public Heal* 2016;**4**:272. doi:10.3389/fpubh.2016.00272

2 Mira JJ, Navarro I, Botella F, *et al.* A Spanish pillbox app for elderly patients taking multiple medications: randomized controlled trial. *J Med Internet Res* 2014;**16**:e99. doi:10.2196/jmir.3269

3 Michie S, Richardson M, Johnston M, *et al.* The Behavior Change Technique Taxonomy (v1) of 93 Hierarchically Clustered Techniques: Building an International Consensus for the Reporting of Behavior Change Interventions. *Ann Behav Med* 2013;**46**:81–95. doi:10.1007/s12160-013-9486-6

4 Lakshminarayana R, Wang D, Burn D, *et al.* Using a smartphone-based self-management platform to support medication adherence and clinical consultation in Parkinson ' s disease. *NPJ Park Dis* 2017;**3**:1–8. doi:10.1038/s41531-016-0003-z

5 Kassavou A, Sutton S. Automated telecommunication interventions to promote adherence to cardio-metabolic medications: meta-analysis of effectiveness and meta-regression of behaviour change techniques. *Health Psychol Rev* 2018;**12**:25–42. doi:10.1080/17437199.2017.1365617

6 Santo K, Singleton A, Rogers K, *et al.* Medication reminder applications to improve adherence in coronary heart disease: a randomised clinical trial. *Heart* 2018;:heartjnl-2018-313479. doi:10.1136/heartjnl-2018-313479

7 Hammonds T, Rickert K, Goldstein C, *et al.* Adherence to Antidepressant Medications: A Randomized Controlled Trial of Medication Reminding in

For peer review only - http://bmjopen.bmj.com/site/about/guidelines.xhtml

College Students. 2015;**8481**. doi:10.1080/07448481.2014.975716

8 Labovitz DL, Shafner L, Gil MR, *et al.* Using Artificial Intelligence to Reduce the Risk of Nonadherence in Patients on Anticoagulation Therapy. *Stroke* 2017;**48**:1416–9. doi:10.1161/STROKEAHA.116.016281

9 Lakshminarayana R, Wang D, Burn D, *et al.* and adherence tools to manage Parkinson ' s disease (SMART-PD): study protocol for a randomised controlled trial (v7 ; 15 August 2014). 2014;:1–11.

10 Santo K, Chow CK, Thiagalingam A, *et al.* MEDication reminder APPs to improve medication adherence in Coronary Heart Disease (MedApp-CHD) Study : a randomised controlled trial protocol. 2017;:1–10. doi:10.1136/bmjopen-2017-017540

11 Morawski K, Ghazinouri R, Krumme A, *et al.* Association of a Smartphone Application With Medication Adherence and Blood Pressure Control. *JAMA Intern Med* 2018;**178**:802. doi:10.1001/jamainternmed.2018.0447

For peer review only

PRISMA 2009 Checklist

Section/topic	#	Checklist item	Reported on page #
**TITLE**			
Title	1	Identify the report as a systematic review, meta-analysis, or both.	1
**ABSTRACT**			
Structured summary	2	Provide a structured summary including, as applicable: background; objectives; data sources; study eligibility criteria, participants, and interventions; study appraisal and synthesis methods; results; limitations; conclusions and implications of key findings; systematic review registration number.	2-3
**INTRODUCTION**			
Rationale	3	Describe the rationale for the review in the context of what is already known.	4-5
Objectives	4	Provide an explicit statement of questions being addressed with reference to participants, interventions, comparisons, outcomes, and study design (PICOS).	6
**METHODS**			
Protocol and registration	5	Indicate if a review protocol exists, if and where it can be accessed (e.g., Web address), and, if available, provide registration information including registration number.	5
Eligibility criteria	6	Specify study characteristics (e.g., PICOS, length of follow-up) and report characteristics (e.g., years considered, language, publication status) used as criteria for eligibility, giving rationale.	6
Information sources	7	Describe all information sources (e.g., databases with dates of coverage, contact with study authors to identify additional studies) in the search and date last searched.	5
Search	8	Present full electronic search strategy for at least one database, including any limits used, such that it could be repeated.	Supplementary file p 2
Study selection	9	State the process for selecting studies (i.e., screening, eligibility, included in systematic review, and, if applicable, included in the meta-analysis).	6
Data collection process	10	Describe method of data extraction from reports (e.g., piloted forms, independently, in duplicate) and any processes for obtaining and confirming data from investigators.	6-7
Data items	11	List and define all variables for which data were sought (e.g., PICOS, funding sources) and any assumptions and simplifications made.	6-8 and Table 1, p21
Risk of bias in individual studies	12	Describe methods used for assessing risk of bias of individual studies (including specification of whether this was done at the study or outcome level), and how this information is to be used in any data synthesis.	7
Summary measures	13	State the principal summary measures (e.g., risk ratio, difference in means).	7
Synthesis of results	14	Describe the methods of handling data and combining results of studies, if done, including measures of consistency (e.g., I^2) for each meta-analysis.	7-8

For peer review only

For peer review only - http://bmjopen.bmj.com/site/about/guidelines.xhtml

PRISMA 2009 Checklist

Section/topic	#	Checklist item	Reported on page #
Risk of bias across studies	15	Specify any assessment of risk of bias that may affect the cumulative evidence (e.g., publication bias, selective reporting within studies).	7
Additional analyses	16	Describe methods of additional analyses (e.g., sensitivity or subgroup analyses, meta-regression), if done, indicating which were pre-specified.	7
**RESULTS**			
Study selection	17	Give numbers of studies screened, assessed for eligibility, and included in the review, with reasons for exclusions at each stage, ideally with a flow diagram.	8
Study characteristics	18	For each study, present characteristics for which data were extracted (e.g., study size, PICOS, follow-up period) and provide the citations.	P8-9 and Table 1, p 21
Risk of bias within studies	19	Present data on risk of bias of each study and, if available, any outcome level assessment (see item 12).	P10 and Table 2, p 23
Results of individual studies	20	For all outcomes considered (benefits or harms), present, for each study: (a) simple summary data for each intervention group (b) effect estimates and confidence intervals, ideally with a forest plot.	Figure 2, (and eTable 1 for weighting of each study and eTable 2 for raw extracted data found in supplementary file, p 4-5)
Synthesis of results	21	Present results of each meta-analysis done, including confidence intervals and measures of consistency.	9
Risk of bias across studies	22	Present results of any assessment of risk of bias across studies (see Item 15).	11 and efigure 1 on p 3 of supplementary material file
Additional analysis	23	Give results of additional analyses, if done (e.g., sensitivity or subgroup analyses, meta-regression [see Item 16]).	11
**DISCUSSION**			

For peer review only

PRISMA 2009 Checklist

Summary of evidence	24	Summarize the main findings including the strength of evidence for each main outcome; consider their relevance to key groups (e.g., healthcare providers, users, and policy makers).	11
Limitations	25	Discuss limitations at study and outcome level (e.g., risk of bias), and at review-level (e.g., incomplete retrieval of identified research, reporting bias).	13
Conclusions	26	Provide a general interpretation of the results in the context of other evidence, and implications for future research.	14
**FUNDING**			
Funding	27	Describe sources of funding for the systematic review and other support (e.g., supply of data); role of funders for the systematic review.	19

From: Moher D, Liberati A, Tetzlaff J, Altman DG, The PRISMA Group (2009). Preferred Reporting Items for Systematic Reviews and Meta-Analyses: The PRISMA Statement. PLoS Med 6(7): e1000097. doi:10.1371/journal.pmed1000097

For more information, visit: **www.prisma-statement.org**.

For peer review only

For peer review only - http://bmjopen.bmj.com/site/about/guidelines.xhtml

BMJ Open

Do mobile device applications designed to support medication adherence demonstrate efficacy? A systematic review of randomised controlled trials, with meta-analysis

Journal:	*BMJ Open*
Manuscript ID	bmjopen-2019-032045.R2
Article Type:	Original research
Date Submitted by the Author:	27-Nov-2019

Complete List of Authors:	Armitage, Laura; University of Oxford, Nuffield Department of Primary Care Health Sciences;<br>Kassavou, Aikaterini; University of Cambridge, Public Health and Primary Care ; University of Cambridge,<br>Sutton, Stephen; University of Cambridge,

**Primary Subject Heading**:	Public health
Secondary Subject Heading:	General practice / Family practice, Patient-centred medicine
Keywords:	Telemedicine < BIOTECHNOLOGY & BIOINFORMATICS, PRIMARY CARE, PUBLIC HEALTH, World Wide Web technology < BIOTECHNOLOGY & BIOINFORMATICS, GENERAL MEDICINE (see Internal Medicine)

SCHOLARONE™
Manuscripts

I, the Submitting Author has the right to grant and does grant on behalf of all authors of the Work (as defined in the below author licence), an exclusive licence and/or a non-exclusive licence for contributions from authors who are: i) UK Crown employees; ii) where BMJ has agreed a CC-BY licence shall apply, and/or iii) in accordance with the terms applicable for US Federal Government officers or employees acting as part of their official duties; on a worldwide, perpetual, irrevocable, royalty-free basis to BMJ Publishing Group Ltd ("BMJ") its licensees and where the relevant Journal is co-owned by BMJ to the co-owners of the Journal, to publish the Work in this journal and any other BMJ products and to exploit all rights, as set out in our licence.

The Submitting Author accepts and understands that any supply made under these terms is made by BMJ to the Submitting Author unless you are acting as an employee on behalf of your employer or a postgraduate student of an affiliated institution which is paying any applicable article publishing charge ("APC") for Open Access articles. Where the Submitting Author wishes to make the Work available on an Open Access basis (and intends to pay the relevant APC), the terms of reuse of such Open Access shall be governed by a Creative Commons licence – details of these licences and which Creative Commons licence will apply to this Work are set out in our licence referred to above.

Other than as permitted in any relevant BMJ Author's Self Archiving Policies, I confirm this Work has not been accepted for publication elsewhere, is not being considered for publication elsewhere and does not duplicate material already published. I confirm all authors consent to publication of this Work and authorise the granting of this licence.

For peer review only

Title

Do mobile device applications designed to support medication adherence demonstrate efficacy? A systematic review of randomised controlled trials, with meta-analysis

Word Count of Manuscript Text: 3524

Author Details:

Laura Armitage, MRCGP[1,2,3]

Aikaterini Kassavou, PhD[1]

Stephen Sutton, PhD[1]

Affiliations:

1. Department of Public Health and Primary Care, University of Cambridge, UK
2. National Institute for Health Research, Collaboration for Leadership in Applied Health Research and Care, East of England, UK
3. Nuffield Department of Primary Care Health Sciences, University of Oxford, UK

Author for Correspondence:

Laura Armitage

Email: laura.armitage@phc.ox.ac.uk Tel: +447791366355

Nuffield Department of Primary Care Health Sciences,

Radcliffe Primary Care Building, Radcliffe Observatory Quarter,

Woodstock Road,

Oxford,

OX2 6GG

For peer review only

ABSTRACT

Objectives

To estimate the efficacy of app-based interventions designed to support medication adherence and investigate which behaviour change techniques (BCTs) utilised by applications (apps) are associated with efficacy.

Design

Systematic review of randomised controlled trials (RCTs), with meta-analysis.

Setting

Medline/PubMed, PsycINFO, CINAHL, Embase and Web of Science were searched from 1990 to November 2018 for RCTs conducted in any health care setting.

Participants

Studies of participants of any age, taking prescribed medication for any health condition, for any duration.

Intervention

An app-based intervention delivered through a smartphone, tablet computer or personal digital assistant to help, support or advise about medication adherence.

Comparator

One of (i) usual care; (ii) a control app which did not use any BCTs to improve medication adherence or (iii) a non-app based comparator.

Primary and Secondary Outcome Measures

For peer review only - http://bmjopen.bmj.com/site/about/guidelines.xhtml

The primary outcome was the pooled effect size of changes in medication adherence. The secondary outcome was an association between BCTs utilised by apps and effect size.

Results

The initial search identified 13,259 citations. After title and abstract screening, full text articles of 83 studies were screened for eligibility. Nine RCTs with 1159 recruited participants were included. Mean age of participants was >50 years in all but one study. Health conditions of target populations included cardiovascular disease, depression, Parkinson's disease, psoriasis and multimorbidity. Meta-analysis indicated that patients who use mobile apps to support medication taking are more likely to adhere to medications (OR 2.120, 95% CI 1.635-2.747, N=988) than those in comparator groups. Meta-regression of the BCTs did not reveal any significant associations with effect size.

Conclusions

App-based medication adherence interventions may have a positive effect on patient adherence. Larger scale studies are required to further evaluate this effect, including long-term sustainability and characteristics of the intervention and participants which are associated with efficacy and app usage.

Article Summary – Strengths and Limitations of this Study

- This systematic review and meta-analysis of randomised controlled trials offers new information on the efficacy of mobile device applications to support medication adherence across a range of medical conditions.
- This review is reported according to the PRISMA Statement.

For peer review only

For peer review only - http://bmjopen.bmj.com/site/about/guidelines.xhtml

- A sensitive search strategy was designed and screening of articles and data extraction was performed independently by one clinical researcher and one non-clinical researcher.
- The review protocol was prospectively registered and the scope of the review, inclusion and exclusion criteria remained unchanged throughout.
- Two reviewers independently considered abstracts and full text articles for inclusion and extracted data and assessed included studies for risk of bias in the same way.

For peer review only

For peer review only - http://bmjopen.bmj.com/site/about/guidelines.xhtml

MANUSCRIPT TEXT

Introduction

Adherence to medication is defined as the extent to which patients take medications as prescribed and agreed with their health care providers.[1] The therapeutic benefit of prescribed medications is limited owing to an estimated 50% of patients not adhering to medications.[2] The annual cost of non-adherence is estimated to exceed £930 million in England[3] and between $100 and $300 billion in the US.[4] Furthermore, in 2003 the Adherence to Long-term Therapy report published by the World Health Organisation stated that unless medication adherence is addressed, advances in biomedical technology will not achieve their potential.[5]

Interventions for improving adherence should be reliable, contemporary, acceptable and readily available to the person. Mobile devices can meet these needs and in 2018, Ofcom reported that 78% of adults and 95% of 16-24 year olds in the UK own a smartphone, demonstrating that smartphones are a growing part of modern life.[6] Patients who are prescribed multiple medications are commonly in the older age group, where the percentage of smartphone users may be lower. However, smartphone ownership in those aged over 65 more than trebled between the years 2012 and 2015 from 5% to 18%, and is set to continue to increase. [7]

Hundreds of applications (hereafter *apps*) are now available to patients to support them in taking regular medication.[8] Such apps frequently utilise behaviour change techniques (BCTs) to promote improvements in adherence. A BCT is defined as an active component of an intervention that is designed to change behaviour.[9] To date, the efficacy of smartphone apps to support patient adherence to regular prescribed medications has not been evaluated within the scientific literature using rigorous quantitative review. We have performed a novel systematic review with meta-analysis to evaluate the efficacy of smartphone apps in supporting medication adherence.

For peer review only - http://bmjopen.bmj.com/site/about/guidelines.xhtml

The objectives of this review are (i) to establish whether apps designed to support medication adherence demonstrate efficacy and (ii) to identify the intervention characteristics and Behaviour Change Techniques (BCTs) associated with efficacy. The conclusions drawn have potential to inform future large-scale public health interventions for the improvement of medication adherence.

METHODS

This systematic review is reported according to the PRISMA statement.[10] The protocol for the review is registered with and published on the PROSPERO database of systematic reviews (registration number: CRD42017080150).[11] The scope of the review, inclusion and exclusion criteria remained unchanged throughout.

Patient and Public Involvement

This research was done without patient involvement. Patients were not invited to comment on the study design and were not consulted to develop patient relevant outcomes or interpret the results. Patients were not invited to contribute to the writing or editing of this document for readability or accuracy.

Data Sources

Medline/PubMed, PsycINFO, CINAHL, Embase and Web of Science electronic databases were searched from 1990 to November 2018. A sensitive search strategy was designed based on preliminary searches, relevant papers and key words. Key search terms included adherence, non-adherence, smart phone app and randomised control trials (RCTs). An example detailed search strategy for the Medline/PubMed database can be found in supplementary information, (eText 1). Reference lists of included studies were screened for additional relevant trials.

For peer review only - http://bmjopen.bmj.com/site/about/guidelines.xhtml

Two reviewers (LA and AK) independently screened all citations by title and abstract and excluded those which were irrelevant or clearly did not meet eligibility criteria. Full text articles were obtained for all remaining citations and screened against the eligibility criteria by the same reviewers. Any discrepancies at any stage in the screening process were settled through discussion between LA, AK and a third reviewer (SS).

Study Selection

RCTs relevant to this review were those that investigated the use of an app on a mobile device to support medication adherence. Inclusion criteria were as follows:

(i) Participants of any age who were taking one or more prescribed medications for any health condition, for any duration.

(ii) An intervention group which received an app-based intervention delivered through a smartphone, tablet computer or personal digital assistant to help, support or advise about medication adherence.

(iii) A comparator group which received usual care, a control condition which did not utilise an app,[12] or a control app which did not include any BCTs (for example an app that was a non-health related game[13]).

(iv) Medication adherence data reported for both the intervention and comparator groups.

Exclusion criteria were as follows:

(i) Complex interventions of which an app was just one component.

(ii) Interventions delivered by text messaging or telemedicine.

(iii) Outcome assessed was adherence to treatment components other than prescribed medication. For example one study reported outcome data as a composite score, combining adherence to other aspects of the medical regimen (such as clinic attendance and laboratory work) with adherence to medication.[14]

For peer review only - http://bmjopen.bmj.com/site/about/guidelines.xhtml

(iv) A study population of healthy recruits who were required to adhere to a placebo medication.

(v) Comparator group received an adherence intervention in another format, such as a different medication adherence app.

Published articles were required to be in English language.

Data Extraction and synthesis

Data were extracted independently by two reviewers (LA and AK) using a custom data extraction form. Participant baseline characteristics were extracted, including age and sex. Raw data on medication adherence were extracted either from the published articles or by contacting the authors of the primary studies by e-mail. When two measures of adherence were reported, that which was considered most comparable to the outcome measures of the other included studies was used for meta-analysis. For example, one study reported adherence according to both the number of times an electronically chipped dispenser was actioned by the patient and the weight of the dispensing cannister at study follow up, and the reviewers agreed that the action of dispensing was most comparable to that of the other included studies.[15] Authors were contacted by email for any other data required which was not available in the published articles. Where more than one app intervention was tested, data for the intervention considered most comparable to the other studies was extracted for meta-analysis. For example, one study investigated a ‘basic app’ (which provided a uni-directional once daily reminder) as well as an ‘advanced app’ (which provided customisable and interactive features) versus usual care.[16] The reviewers agreed that the advanced app was most comparable to the interventions of the other studies and so data pertaining to this intervention was extracted. BCTs that were utilised in the app-based interventions were coded according to the Behaviour Change Taxonomy.[17] This is a structured taxonomy of techniques utilised by interventions to change behaviour. The BCT Taxonomy does not have a code relating to performance of behaviour and therefore where participants were required to enter whether or not

For peer review only - http://bmjopen.bmj.com/site/about/guidelines.xhtml

medication was taken, this was coded as 'Report whether or not the behaviour was performed'. Similarly, the Taxonomy does not have a code relating to tailoring of an intervention and therefore where a study used information gained about a person to define the content, frequency or format of an intervention, this was coded as a 'Tailored' intervention.[18]

The included trials were assessed for risk of bias using the Cochrane Risk of Bias Tool.[19] Two reviewers (LA and AK) individually assessed each of the included articles against criteria outlined in the tool. Any discrepancies at any stage in the coding process were settled through discussion between the three reviewers (LA, AK, SS). Studies were considered at unclear risk of bias for an item when the primary article did not provide sufficient information for the reviewers to decide categorically between high or low risk of bias.

Statistical Analysis

Outcome data on medication adherence were extracted from each of the included studies at the final observation point. Authors were contacted for raw final observation point adherence data if this was not reported in published manuscripts. Trials were considered homogeneous based on the content of the intervention and comparator groups and the outcome measurement of medication adherence. Comprehensive Meta-Analysis software[20] was used to estimate the overall effect of app-delivered interventions on medication adherence using a random-effects model and to perform meta-regression using the BCTs utilised by the apps. Comprehensive Meta-analysis Software conversion function was used where required to convert extracted raw data into odds ratio and standardised mean difference between the intervention and control groups.[21] The I^2 statistic was used to estimate heterogeneity between included studies.

RESULTS

For peer review only - http://bmjopen.bmj.com/site/about/guidelines.xhtml

The initial electronic database search identified 13 259 potentially eligible studies, and two were identified from reference lists of identified articles. After removal of duplicates, 9971 citations were screened by title and abstract. We considered 83 of these to be eligible and retrieved the full-text articles. Of these, nine studies reported outcome data eligible for meta-analysis[12,13,15,16,22–26]. The screening process and reasons for exclusion are reported in Figure 1.

Study characteristics of the nine studies are displayed in Table 1. Publication year of included studies ranged from 2014-2018. The median sample size was 102 participants (range 24-412). The median follow-up period was 12 weeks (range 28 days to 16 weeks).

Mean age of participants in the intervention group was reported by 8 studies and ranged from 20.3 (SD 4.0)[22] to 73.8 (SD 8)[12]; the mean of the mean ages across these 8 studies was 56.7 years. One did not report the mean age but presented the percentage of participants whose age fell within a series of bandwidths.[15] The percentage of participants in intervention groups who were female ranged from 11%[16] to 89%[22], and the mean percentage of females among the included studies was 45.2%.

Health problem of the target population varied between studies. Five studies sampled a patient population with cardiovascular disease[12,13,16,23,26] and each of the remaining four studies enrolled patient populations with different health problems: depression[22], Parkinson's disease,[24] psoriasis[15] and multimorbidity.[25]

Outcome Measurement Methods

A number of measures of medication adherence were utilised in the included studies. Of the 9 studies, four measured adherence using the 8 Item Morisky Medication Adherence Scale[24,26,27], with one of these also measuring adherence through a medication possession ratio (see Supplementary Material file, Shah et al, 2016).[13] For this study, the Morisky Medication Adherence Scale data was extracted for the meta-analysis as this was most comparable to the data

For peer review only - http://bmjopen.bmj.com/site/about/guidelines.xhtml

available for other studies. A further study measured the 4 Item Morisky Medication Adherence Scale.[25] Of the remaining studies, two reported adherence according to pill count[22,23], one the A14 self-report scale of medication adherence[12] and one through a digital chip record fitted within the medication dispenser (Table 1).[15]

Meta-analysis

Meta-analysis was performed, pooling results from the nine eligible studies which reported data on 988 participants, to estimate the effect of mobile apps to improve medication adherence.[12,13,15,16,22–26] The result of this meta-analysis indicated that patients who participated in medication adherence interventions delivered by mobile apps were more likely to adhere to prescribed medications (OR 2.120, 95% CI 1.635-2.747) than those that did not use such interventions. The I^2 value was 9.8 indicating low statistical heterogeneity between the studies included in the meta-analysis. The forest plot of the meta-analysis can be seen in Figure 2. The weighting of each individual study in the meta-analysis can be seen in eTable 1, and raw data extracted for the meta-analysis can be found in eTable 2.

A focussed meta-analysis was performed, pooling results from the 5 studies which reported medication adherence according to the Morisky Medication Adherence Scale.[13,16,24–26] The result of this also indicated that patients using medication adherence interventions delivered by mobile apps reported higher adherence to prescribed medications (OR 1.83, 95% CI 1.42-2.36).

Behaviour Change Techniques

The most common BCT utilised by apps was ‘Tailored”, which was utilised by all studies. The next most common was ‘Prompts and Cues’, utilised by seven studies[12,15,16,22–25] and ‘Report whether or not the behaviour was performed’ which was utilised by six studies.[12,16,22–25] Information on the BCTs coded for each of the interventions and definitions of each of the BCTs is available in eTables 3 and 4. Agreement between reviewers for classification of BCTs utilised by the

For peer review only - http://bmjopen.bmj.com/site/about/guidelines.xhtml

interventions was assessed using the kappa statistic. This revealed a 'very good' level of agreement (kappa = 0.902, 95% CI 0.835 – 0.969). All BCTs coded for each of the study interventions are provided in eTable3. Those coded in more than three but less than 6 of the 9 studies were included in the meta-regression; this did not reveal any significant associations between the BCTs utilised and effect size.

Risk of bias at primary study level

A summary of the risk of bias analysis can be seen in Table 2**.** Overall, all studies were deemed as unclear in their risk of bias.

The most common source of risk of bias amongst the studies was the absence of blinding of participants and personnel to the outcome measure. The second most common risk of bias arose from a lack of objective measurements of medication adherence. This was due to the use of self-report measures of adherence: six of the studies included in the meta-analysis reported adherence based on a self-assessment scale[12,13,16,24–26], and one study each used patient-entered medication logs in an app[22], an electronically chipped medicated foam dispenser[15] and pill counts at clinic visits.[23]

Self-assessment scales are a form of self-report and therefore are a source of potential bias, including those of social desirability, and those arising from selective recall and the duration of the recall period.[28] Two studies included in this review, which measured adherence through participant self-report, sought to compare their subjective measurements of adherence with serum measurements of either drug-reactivity units or a medication's anticoagulant effect.[13,23] One study showed that serum measurement of the medication's anticoagulant effect was similar between both the intervention and comparator groups, despite adherence measures being better in the intervention group.[23] The other study revealed that drug-reactivity units were worse in the

For peer review only - http://bmjopen.bmj.com/site/about/guidelines.xhtml

intervention group than the control group, which conflicted with measured adherence outcome data.[13] However, the presence of a drug biomarker does not equate to compliance and the absence does not equate to non-compliance.[29] Indeed, drug metabolism should be taken into account for such measures of medication adherence, as physiological state and metabolic rate vary among individuals.[30]

Publication Bias

A funnel plot of effect size estimates of each of the studies included in the meta-analysis is provided in the online supplementary file (eFigure 1). Formal analysis of plot symmetry was not performed owing to the total number of studies included being less than 10.[31] Visual inspection reveals moderate symmetry of the plot.

DISCUSSION

Principal Findings

We conducted a rigorous systematic review of the literature to investigate whether mobile device apps demonstrate efficacy in supporting medication adherence. A random-effects meta-analysis of the pooled results from all nine studies indicated that people who use medication reminder apps are significantly more likely to adhere to their medications than those who do not, but the results should be interpreted with caution owing to data for 6 of the 9 studies included in the meta-analysis being based on self-reported measures of adherence.

Intervention effects reported between the studies varied, as shown in the forest plot (Figure 2). All nine studies reported that their mobile app-delivered interventions demonstrated efficacy at supporting medication adherence, although in five studies the interventions did not have a statistically significant effect.[12,13,16,22,25] Although the I^2 measure of statistical heterogeneity was low, and meta-regression of the BCTs utilised by the studies was not able to account for variance in effect observed between studies, there are other potential sources of methodological

For peer review only - http://bmjopen.bmj.com/site/about/guidelines.xhtml

and clinical heterogeneity which may account for this. One potential explanation might be the variation in adherence measurement. This may be illustrated by three studies which incorporated five of the same BCTs[23–25] but where one[23] reported better medication adherence (OR 10.633, 95% CI 2.339-48.344) than the other two (OR 1.946, 95% CI 1.094-3.461 and OR 1.251, 95% CI 0.612-2.559).[24,25] In Labovitz et al’s study, the medication adherence was measured by pill counts at clinic follow up visits[23], whereas in the other two studies, adherence was measured by retrospective self-scoring on a medication adherence scale[24,25] It could be argued that both of these different measures of adherence are vulnerable to bias with pill counts potentially being vulnerable to expectation bias and retrospective self-scores being vulnerable to recall bias. We subsequently performed a focused meta-analysis of the five studies which measured adherence using the Morisky Medication Adherence Scale to investigate whether this influenced the effect size observed. Whilst the result of meta-analysis remained significant, the effect size was lower. This may support the use of real-time or prospective adherence monitoring over retrospective self-report for future research in the field of medication adherence.

A further possible explanation for variance in effect size might be techniques, beyond those reported in the BCT taxonomy, utilised by the apps. For example, in the study by Labovitz et al, the app intervention employed artificial intelligence to identify the participant, their medication and ingestion of the medication, using the camera of the mobile device and software algorithms. This could be regarded as a highly tailored intervention and one might hypothesise that the degree of tailoring could influence the effect size observed. A further example of a highly tailored intervention was seen in the study by Lakshminarayana et al. All of the app-based interventions in the included studies were tailored to the prescribed medication regimen, but in this instance the tailoring went beyond the prescribed medication regimen, to include tailoring to the patients’ intentional and non-intentional non-adherence, beliefs about medication taking, mood, cognitive impairment, symptom control, quality of life, age and disease duration. A significant effect size was observed in this study,

For peer review only - http://bmjopen.bmj.com/site/about/guidelines.xhtml

and this may support a hypothesis that highly tailored apps are more effective at supporting medication adherence, than minimally tailored apps.

Further factors which may have contributed to variance in the effect size seen between studies relate to the participants, including disease burden, non-adherence behaviours, perceived benefit from the medication and any side effects. Interpretation of the overall result of the meta-analysis and generalisability to the wider population should therefore be cautious.

Strengths and Weaknesses of the Primary Studies

Based on the assessment of the risk of bias undertaken as part of this review, the evidence included in this meta-analysis is of moderate quality. The most common weaknesses in this evidence are small sample size, lack of objectivity of the outcome measurement, a lack of blinding of participants and personnel and limited follow up time.

Strengths and Weaknesses at Review Level

Our comprehensive literature search and search of the PROSPERO Database indicate this is the first systematic review and meta-analysis to investigate the efficacy of mobile apps to support medication adherence. This offers new knowledge to the field; however, as such there are no comparable studies to which the results of this review can be compared.

The pooling of data from a group of studies which used a variety of methods for measurement of medication adherence and targeted at patients with different healthcare problems is a limitation of this review. As further studies in this field are published it will become more feasible to conduct subgroup analysis or meta-regression analyses against variables such as age, health condition, targeted medication, intervention content and app characteristics. A standardised protocol for measuring adherence and reporting the content and characteristics of app interventions may facilitate further analysis of the latter two variables.

For peer review only - http://bmjopen.bmj.com/site/about/guidelines.xhtml

This review cannot report on the likely sustainability of the effect on medication adherence given the short study duration of the studies included in this review; across the 9 studies, the maximum follow up period was 16 weeks. Furthermore, the optimal frequency and duration of app use that is required to achieve improvements in medication adherence is not known. Long term and large-scale research is required in this field to addresses the question regarding sustainability of the observed effect found in this meta-analysis.

This systematic review and meta-analysis included studies with a range of ages of participants. The lowest mean age of participants in the included studies was 20.9 years and the highest was 70.9 years. The ubiquity and use of mobile devices varies across age groups[7], and whilst the majority of participants in these studies were aged 50 and over, further research to establish the acceptability and usability of adherence apps among different age groups adults would be valuable.

Finally, the reviewers set out to perform a meta-regression of the BCTs utilised by app interventions in the included studies.[11] However, this did not reveal any significant association between the BCTs utilised and observed effect size. This may be owing to the low estimate of heterogeneity and the small number of eligible included studies.

Conclusions

This meta-analysis indicates that medication adherence interventions delivered by smartphone apps can be efficacious in improving patients' adherence to their prescribed medications. However, when considering whether to recommend an app to patients, these results should be interpreted with caution owing to variance in effect size seen between the studies, the small sample sizes taken from different patient populations, heterogeneity among included studies and the prominence of self-reported scales to measure medication adherence. Further research should aim to first establish a standard protocol for the measuring and reporting of adherence in the context of evaluating digital

For peer review only - http://bmjopen.bmj.com/site/about/guidelines.xhtml

interventions. Subsequently, further research investigating intervention characteristics associated with an app’s effect and the sustainability of any effect gained would add to this field.

.

For peer review only

For peer review only - http://bmjopen.bmj.com/site/about/guidelines.xhtml

References

1 Osterberg L, Blaschke T. Adherence to Medication. *N Engl J Med* 2005;**353**:487–97. doi:10.1056/NEJMra050100

2 Brown MT, Bussell JK. Medication Adherence: WHO Cares? *Mayo Clin Proc* 2011;**86**:304. doi:10.4065/MCP.2010.0575

3 Elliott RA, Boyd MJ, Waring J, *et al.* Understanding & evaluating the New Medicines Service in the NHS in England (029/0124). 2014. http://www.nottingham.ac.uk/~pazmjb/nms/downloads/report/files/assets/common/downloads/108842 A4 Main Report.v4.pdf (accessed 12 Feb 2018).

4 Iuga AO, McGuire MJ. Adherence and health care costs. *Risk Manag Healthc Policy* 2014;**7**:35–44. doi:10.2147/RMHP.S19801

5 Sabaté E. Evidence for action. *World Heal Organ* Published Online First: 2003.http://www.who.int/chp/knowledge/publications/adherence_introduction.pdf (accessed 12 Feb 2018).

6 Ofcom. Communications Market Report. 2018. https://www.ofcom.org.uk/__data/assets/pdf_file/0022/117256/CMR-2018-narrative-report.pdf (accessed 3 Aug 2018).

7 Ofcom. The Communications Market Report. 2015. https://www.ofcom.org.uk/__data/assets/pdf_file/0022/20668/cmr_uk_2015.pdf (accessed 12 Feb 2018).

8 Ahmed I, Ahmad NS, Ali S, *et al.* Medication Adherence Apps: Review and Content Analysis. *JMIR mHealth uHealth* 2018;**6**:e62. doi:10.2196/mhealth.6432

For peer review only

For peer review only - http://bmjopen.bmj.com/site/about/guidelines.xhtml

9 Wideman TH, Sullivan MJL, Inada S, *et al.* Behaviour Change Techniques. In: Gellman Marc D., Turner J. Rick., eds. *Encyclopedia of Behavioral Medicine*. New York, NY: : Springer New York 2013. 12. doi:10.1007/978-1-4419-1005-9_1661

10 Moher D, Liberati A, Tetzlaff J, *et al.* Preferred Reporting Items for Systematic Reviews and Meta-Analyses: The PRISMA Statement. *PLoS Med* 2009;**6**:e1000097. doi:10.1371/journal.pmed.1000097

11 Armitage L, Kassavou A, Sutton S. A systematic review of the literature with met-analysis and meta-regression: the use of smartphone applications to support medication adherence/. PROSPERO Int. Prospect. Regist. Syst. Rev. 2017. doi:CRD42017080150

12 Mertens A, Brandl C, Miron-shatz T, *et al.* A mobile application improves therapy-adherence rates in elderly patients undergoing rehabilitation. *Medicine (Baltimore)* 2016;**95**:e4446.

13 Shah V, Dileep A, Dickens C, *et al.* Patient-Centered Tablet Application for Improving Medication Adherence after a Drug-Eluting Stent. *Front public Heal* 2016;**4**:272. doi:10.3389/fpubh.2016.00272

14 DeVito Dabbs A, Song MK, Myers BA, *et al.* A Randomized Controlled Trial of a Mobile Health Intervention to Promote Self-Management After Lung Transplantation. *Am J Transplant* 2016;**16**:2172–80. doi:10.1111/ajt.13701

15 Svendsen MT, Andersen F, Andersen KH, *et al.* A smartphone application supporting patients with psoriasis improves adherence to topical treatment: a randomized controlled trial. *Br J Dermatol* 2018;**179**:1062–71. doi:10.1111/bjd.16667

16 Santo K, Singleton A, Rogers K, *et al.* Medication reminder applications to improve adherence in coronary heart disease: a randomised clinical trial. *Heart* 2018;:heartjnl-2018-313479. doi:10.1136/heartjnl-2018-313479

For peer review only - http://bmjopen.bmj.com/site/about/guidelines.xhtml

17 Michie S, Richardson M, Johnston M, *et al.* The Behavior Change Technique Taxonomy (v1) of 93 Hierarchically Clustered Techniques: Building an International Consensus for the Reporting of Behavior Change Interventions. *Ann Behav Med* 2013;**46**:81–95. doi:10.1007/s12160-013-9486-6

18 Kassavou A, Sutton S. Automated telecommunication interventions to promote adherence to cardio-metabolic medications: meta-analysis of effectiveness and meta-regression of behaviour change techniques. *Health Psychol Rev* 2018;**12**:25–42. doi:10.1080/17437199.2017.1365617

19 Higgins JP, Altman DG, Sterne JA. Chapter 8: Assessing risk of bias in included studies. https://training.cochrane.org/handbook/chapter-08 (accessed 14 Feb 2018).

20 Comprehensive Meta-Analysis Software (CMA). https://www.meta-analysis.com/ (accessed 15 Feb 2018).

21 Borenstein M, Hedges L., Higgins PT, *et al. Introduction to Meta-Analysis, Converting Among Effect Sizes*. John Wiley and Sons 2009. https://www.meta-analysis.com/downloads/Meta-analysis Converting among effect sizes.pdf (accessed 8 Mar 2019).

22 Hammonds T, Rickert K, Goldstein C, *et al.* Adherence to Antidepressant Medications: A Randomized Controlled Trial of Medication Reminding in College Students. 2015;**8481**. doi:10.1080/07448481.2014.975716

23 Labovitz DL, Shafner L, Gil MR, *et al.* Using Artificial Intelligence to Reduce the Risk of Nonadherence in Patients on Anticoagulation Therapy. *Stroke* 2017;**48**:1416–9. doi:10.1161/STROKEAHA.116.016281

24 Lakshminarayana R, Wang D, Burn D, *et al.* Using a smartphone-based self-management platform to support medication adherence and clinical consultation in Parkinson ' s disease.

For peer review only - http://bmjopen.bmj.com/site/about/guidelines.xhtml

NPJ Park Dis 2017;**3**:1–8. doi:10.1038/s41531-016-0003-z

25 Mira JJ, Navarro I, Botella F, *et al.* A Spanish pillbox app for elderly patients taking multiple medications: randomized controlled trial. *J Med Internet Res* 2014;**16**:e99. doi:10.2196/jmir.3269

26 Morawski K, Ghazinouri R, Krumme A, *et al.* Association of a Smartphone Application With Medication Adherence and Blood Pressure Control. *JAMA Intern Med* 2018;**178**:802. doi:10.1001/jamainternmed.2018.0447

27 Santo K, Chow CK, Thiagalingam A, *et al.* MEDication reminder APPs to improve medication adherence in Coronary Heart Disease (MedApp-CHD) Study : a randomised controlled trial protocol. 2017;:1–10. doi:10.1136/bmjopen-2017-017540

28 Althubaiti A. Information bias in health research: definition, pitfalls, and adjustment methods. *J Multidiscip Healthc* 2016;**9**:211–7. doi:10.2147/JMDH.S104807

29 Farmer KC. Methods for measuring and monitoring medication regimen adherence in clinical trials and clinical practice. *Clin Ther* 1999;**21**:1074–90. doi:10.1016/S0149-2918(99)80026-5

30 Lam WY, Fresco P. Medication Adherence Measures: An Overview. *Biomed Res Int* 2015;**2015**:217047. doi:10.1155/2015/217047

31 Sterne JAC, Sutton AJ, Ioannidis JPA, *et al.* Recommendations for examining and interpreting funnel plot asymmetry in meta-analyses of randomised controlled trials. *BMJ* 2011;**343**:d4002. doi:10.1136/BMJ.D4002

Sources of funding

For peer review only

For peer review only - http://bmjopen.bmj.com/site/about/guidelines.xhtml

This paper presents independent research funded by the National Institute for Health Research (NIHR) under its Programme Grants for Applied Research Programme (Grant Reference Number RP-PG-0615-20013). The views expressed are those of the authors and not necessarily those of the NIHR or the Department of Health and Social Care. The National Institute for Health Research's (NIHR) Collaboration for Leadership in Applied Health Research and Care (CLAHRC) in East of England provided salary support to LA during conduction of this review. The funders had no role in review design, data collection, data analysis, data interpretation, the writing of the manuscript, and decision to submit the manuscript for publication.

Author Contributions

All authors were involved in design of the review and undertook methodological planning. LA performed and refined the searches in consultation with a medical librarian. LA and KK performed initial screening and data extraction and SS gave screening advice where any disagreements arose. All authors contributed to performing the meta-analysis to data interpretation. LA led the writing and all authors contributed to successive drafts and approved the final manuscript.

Data Availability

All data relevant to the study are included in the article or uploaded as supplementary information

Competing Interests

The authors have no competing interests to declare

Figure Legends

Figure 1. PRISMA Flowchart

Figure 2. Forest Plot of Randomised Trials evaluating a mobile device application to support medication adherence against comparators.

For peer review only - http://bmjopen.bmj.com/site/about/guidelines.xhtml

For peer review only

For peer review only - http://bmjopen.bmj.com/site/about/guidelines.xhtml

Table 1: Study Characteristics

Source (Sample size)	Study Nation	Study Design	Health Problem of Target Population	Recruitment setting	Participant characteristics at recruitment				Application intervention	Outcome measure	Follow up interval
					Intervention group		Comparator group				
					Mean age (SD)	Female, N (%)	Mean age (SD)	Female, N (%)			
Hammonds et al, 2015 (57)	USA	RCT	Depression	College students were recruited via campus-based advertisements and the university research recruitment system	20.3 (4.0)	25 (83)	20.9 (4.7)	24 (89)	Medication reminder app	No. of participants taken ≥80% prescribed tablets per day.	25-35d
Labovitz et al, 2017 (28)	USA	RCT	Ischaemic stroke requiring secondary oral anti-coagulation	Outpatient clinic	58.3 (9.79)	6 (40)	55.5 (16.55)	9 (69)	Artificial intelligence app	Pill count	12w
Lakshmin-arayana et al, 2017 (215)	UK	RCT	Parkinson's disease	outpatient clinic	59.86 (9.13)	34 (36.2)	60.71 (10.26)	45 (42.1)	Parkinson's tracker app	Self-report: 8 Item MMAS	16w
Mertens et al, 2016 (24)	Germany	RCT Cross-over	Cardiovascular disease	Cardiac rehab groups	73.8[a]	12 (50)[a]	73.8[a]	12 (50)[a]	Medication Plan app (version 1.3)	Self-report: A14 scale	28d
Mira et al, 2014 (102)	Spain	RCT	Multi-comorbidity	Community health centres	70.9 (8)	21 (41)	72.9 (6)	23 (48)	ALICE medication self-management app	Self-report: 4 Item MMAS	3m

For peer review only - http://bmjopen.bmj.com/site/about/guidelines.xhtml

Morawski et al, 2018<br>(412)	USA	RCT	Hypertension	Social media, mobile apps, targeted advertisements and online communications	51.7 (10.5)	120 (57.4)	52.4 (10.1)	127 (62.9)	MediSAFE-BP app	Self-report: 8 Item MMAS	12w
Santo et al, 2018<br>(163)	Australia	RCT	Coronary Heart Disease	Inpatient, outpatient and rehabilitative care settings.	58.4 (9.04)	14 (13)	56.8 (8.64)	6 (11)	MedApp-CHD	Self-report: 8 Item MMAS	3m
Shah et al, 2016<br>(24)	USA	RCT	Cardiovascular stent	Patients in the immediate post-operative phase of insertion of a drug-eluting cardiovascular stent	60.5 (8.9)	6 (46)	60.6 (4.1)	4 (36)	MyIDEA education app	Medication possession ratio and Self-report: 8 Item MMAS	90d
Svendsen et al, 2018<br>(134)	Denmark	RCT	Psoriasis	Hospital posters and the dermatology outpatient clinic.	18-40 29[b]<br>41-50 21<br>51-60 26<br>61-75 24	27 (40)	18-40 32[b]<br>41-50 20<br>51-60 24<br>61-75 24	25 (38)	My Pso SmarTop Version 1.0 or Smartop number 0536776	Measured via a digital chip record in the medication dispenser.	28d

MMAS = Morisky Medication Adherence Scale.

[a]Baseline characteristics presented for whole cohort as study design was a randomised cross-over trial; [b]number of participants in each age group (years) presented as mean age not available.

Table 2: Risk of Bias Summary Table

For peer review only - http://bmjopen.bmj.com/site/about/guidelines.xhtml

Source	Allocation sequence adequately:		Free from baseline imbalance?			Blinding of participants and personnel and	Objective outcome assessment	Incomplete outcome data adequately addressed	Free from selective outcome reporting	Was the intervention independent of other bias
	Generated	Concealed	Age	Gender	Adherence					
Hammonds et al, 2015	🟡	🟡	🟢	🟢	🟢	🔴	🔴	🟢	🟢	🟢
Labovitz et al, 2017	🟡	🟡	🟡	🟡	🟡	🔴	🔴	🟢	🟢	🟢
Lakshminarayana et al, 2017	🟢	🟢	🟡	🟢	🟢	🔴	🟡	🟢	🟡	🟢
Mertens et al, 2016	🟡	🟡	🟡	🟡	🟡	🔴	🔴	🟢	🔴	🟡
Mira et al, 2014	🟡	🟡	🟡	🟢	🟢	🔴	🔴	🟡	🟢	🟢
Morawski et al, 2018	🟢	🟡	🟡	🟢	🟢	🟡	🔴	🟡	🟢	🟢
Santo et al, 2018	🟢	🟡	🟡	🟢	🟢	🔴	🔴	🟡	🟢	🟢
Shah et al, 2016	🟢	🟢	🟡	🟡	🟡	🟡	🟡	🟢	🟢	🟢
Svendsen et al, 2018	🟢	🟢	🟡	🟡	🟡	🔴	🟢	🟢	🟢	🟢

🟢 = Low risk of bias, 🟡 = Unclear risk of bias, 🔴 = High risk of bias

For peer review only - http://bmjopen.bmj.com/site/about/guidelines.xhtml

For peer review only

For peer review only - http://bmjopen.bmj.com/site/about/guidelines.xhtml

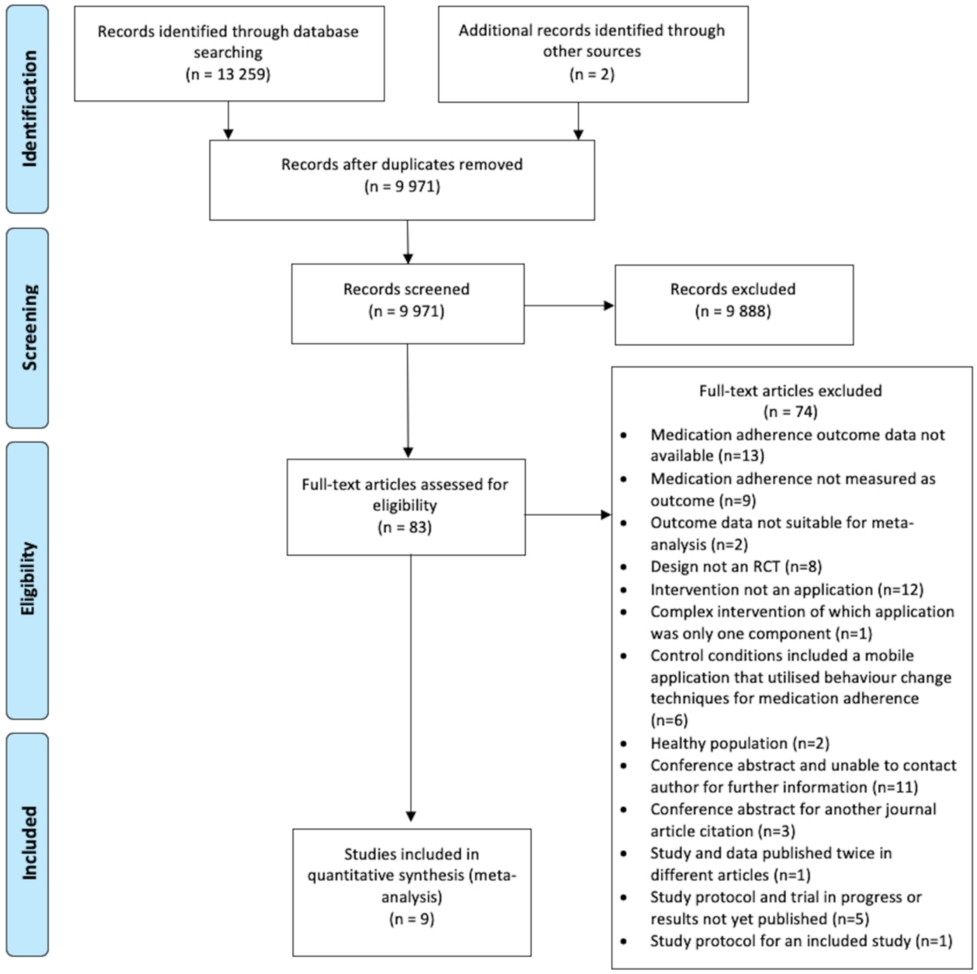

Figure 1. PRISMA Flowchart

305x306mm (300 x 300 DPI)

Study name	Statistics for each study				
	Odds ratio	Lower limit	Upper limit	Z-Value	p-Value
Hammonds 2015	3.500	0.945	12.966	1.875	0.061
Labovitz 2017	10.633	2.339	48.344	3.060	0.002
Lakshminarayana 2017	1.946	1.094	3.461	2.266	0.023
Mertens 2016	2.975	0.675	13.117	1.440	0.150
Mira 2014	1.251	0.612	2.559	0.614	0.539
Morawski 2018	1.896	1.331	2.700	3.547	0.000
Shah 2016	2.206	0.505	9.626	1.052	0.293
Svendsen 2018	3.222	1.526	6.803	3.068	0.002
Santo 2018	2.029	0.991	4.154	1.934	0.053
	2.120	1.635	2.747	5.677	0.000

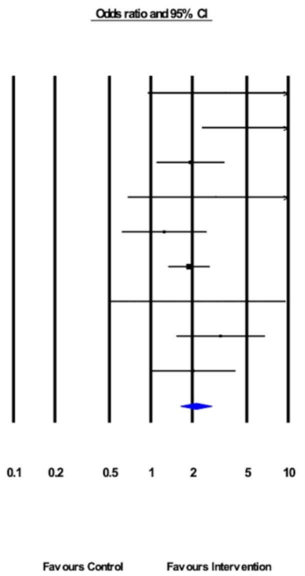

Figure 2. Forest Plot of Randomised Trials evaluating a mobile device application to support medication adherence against comparators.

SUPPLEMENTAL MATERIAL FILE

Mobile device applications to support medication adherence: A systematic review of randomised controlled trials, with meta-analysis

Author Details:

Laura Armitage, MRCGP

Aikaterini Kassavou, PhD

Stephen Sutton, PhD

For peer review only

eText 1: Medline/Pubmed Search Strategy

1. medication adherence.mp. or exp Patient Compliance/ or exp Medication Adherence/
2. nonnadherence.mp.
3. non-adherence.mp.
4. non adherence.mp.
5. concordance.mp.
6. concordant.mp.
7. 1 or 2 or 3 or 4 or 5 or 6
8. app.mp.
9. apps.mp.
10. application.mp.
11. exp Mobile Applications/ or mobile application*.mp.
12. smartphone.mp. or exp Smartphone/ or exp Cell Phones/
13. mobile*.mp.
14. phone*.mp.
15. 8 or 9 or 10 or 11 or 12 or 13 or 14
16. randomized controlled trial.mp. or exp Randomized Controlled Trial/
17. RCT.mp.
18. randomised.mp.
19. randomized.mp.
20. control*.mp.
21. 16 or 17 or 18 or 19 or 20
22. 7 and 15 and 21
23. limit 22 to (english language and yr="1990 - 2017")

For peer review only - http://bmjopen.bmj.com/site/about/guidelines.xhtml

eFigure 1: Funnel Plot of effect estimates from studies included in meta-analysis

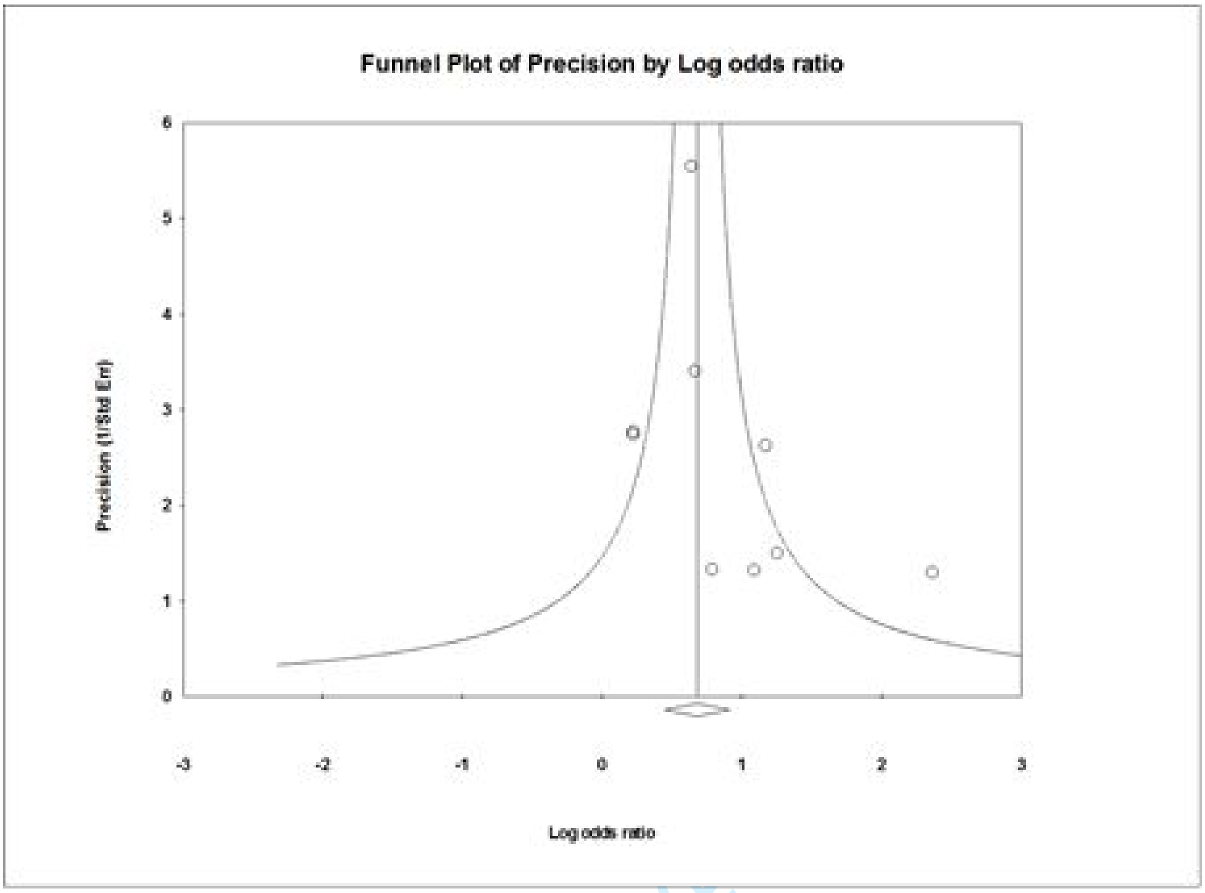

eTable 1: Relative weighting of each study included in the 9 study meta-analysis

Study	Weight (random)	Relative weight
Hammonds et al, 2015	2.16	3.79
Labovitz et al, 2017	1.63	2.86
Lakshminarayana et al, 2017	9.76	17.10
Mertens et al, 2018	1.70	2.97
Mira et al, 2014	6.69	11.72
Morawski et al, 2018	20.58	36.03
Santo et al, 2018	6.68	11.69
Shah et al, 2016	1.72	3.01
Svendsen et al, 2018	6.19	10.84

For peer review only

eTable 2: Raw data extracted for each study for meta-analysis

Study	Adherence measurement	Intervention group				Comparator group			
		Mean (SD)	N	Events	Totals	Mean (SD)	N	Events	Totals
Hammonds et al, 2015	Number of participants taken ≥80% prescribed tablets per day	-	-	12	20	-	-	6	20
Labovitz et al, 2017	Mean cumulative adherence, measured by pill count	97.2 (4.4)	15	-	-	90.6 (5.8)	12	-	-
Lakshminaray-ana et al, 2017	MMAS-8	6.3 (1.52)	68	-	-	5.74 (1.53)	90	-	-
Mertens et al, 2018	A14 Scale	53.96 (2.01)	12	-	-	52.6 (2.49)	12	-	-
Mira et al, 2014	MMAS-4	7.4 (0.9)	51	-	-	7.3 (0.7)	48	-	-
Morawski et al, 2018	MMAS-8	6.3 (1.6)	209	-	-	5.7 (1.8)	202	-	-
Santo et al, 2018	MMAS-8	7.02 (n/a)	50	-	-	6.63 (n/a)	51	-	-
Shah et al, 2016	MMAS-8	2.2 (1.2)	5	-	-	2.1 (1)	3	-	-
Svendsen et al, 2018	Number of patients having applied medication ≥80% days in treatment period	-	-	39	59	-	-	23	61

A14 = A14 Medication Adherence Scale; MMAS-8 = 8 Item Morisky Medication Adherence Scale; MMAS 4 = 4 Item Morisky Medication Adherence Scale

For peer review only

eTable 3: Behaviour Change Techniques utilised by in applications in the included studies.

Study	BCTs Coded							
	Tailored	Prompts and cues	Report whether behaviour was performed	Monitoring of behaviour by others without feedback	Feedback on behaviour	Social support (unspecified)	Habit formation	Goal setting of behaviour
Hammonds et al, 2015	+	+	+	-	-	-	-	-
Labovitz et al, 2017	+	+	+	+	+	-	-	-
Lakshminarayana et al, 2017	+	+	+	+	+	-	-	-
Mertens et al, 2016	+	+	+	-	-	-	-	-
Mira et al, 2014	+	+	+	+	+	-	+	+
Morawski et al, 2018	+	-	+	-	+	+	+	+
Santo et al, 2018	+	-	+	-	+	+	+	-
Shah et al, 2016	+	-	-	-	-	+	-	-
Svendsen et al, 2018	+	-	-	-	+	-	-	-

Those Behaviour Change Techniques coded in 3 but not more than 6 studies were included for meta-regression.

For peer review only - http://bmjopen.bmj.com/site/about/guidelines.xhtml

eTable 4: Definitions of Behaviour Change Techniques coded as present in applications of the included studies and examples of each.

Behaviour Change Technique	Definition	Examples
Tailored	Use information gained about the person to define the content, frequency or format of an intervention directed to the behaviour.	Shah et al, 2016: information about the participant including their symptoms and coronary artery catheterising procedure were recorded and used to tailor the information delivered to the participant via the MyIDEA application.[1]<br><br>Mira et al, 2014: The ALICE app delivered a customised system of alerts and reminders, according to their personalised prescription to remind participants when to take their medication.[2]
Prompts and Cues	*Introduce or define environmental or social stimulus with the purpose of prompting or cueing the behaviour. The prompt and cue would normally occur at the time or place of performance.*[3]	Mira et al, 2014: The ALICE app delivered a customised system of alerts and reminders, according to their personalised prescription to remind participants when to take their medication.[2]<br><br>Lakshminarayana et al, 2017: The app included a reminder system for participants to set up and allow medication alerts.[4]
Report whether or not behaviour was performed	Ask a person to report when they have performed a behaviour without the requirement of monitoring or recording the behaviour.[5]	Santo et al, 2018: Reminders from the application could be snoozed, rescheduled and/or marked as 'taken' or 'missed dose'.[6]<br><br>Hammonds et al, 2015: Participants indicated when they had taken their medication by responding to the alert message.[7]

For peer review only - http://bmjopen.bmj.com/site/about/guidelines.xhtml

Monitoring of behaviour by others without feedback	*Observe or record behaviour with the person's knowledge as part of a behaviour change strategy.*[3]	Labovitz et al, 2017: Clinic staff received automated text messages or emails if doses were missed, late or based on incorrect usage.[8]<br><br>Lakshminarayana et al, 2017: Clinicians had access to a dedicated web portal where they could see the participants' progress over the trial period.[9]
Feedback on behaviour	*Monitor and provide informative or evaluative feedback on performance of the behaviour (e.g. form, frequency, duration, intensity.*[3]	Mira et al, 2014: The ALICE app reported to the participant medication that they had taken that day and those that the participant had not taken.[2]<br><br>Santo et al, 2018: The app included a feature of medication adherence statistics.[10]
Social support (unspecified)	*Advise on, arrange or provide social support (e.g. from friends, relatives, colleagues, buddies or staff) or non-contingent praise or reward for performance of the behaviour. It includes encouragement and counselling, but only when it is directed at the behaviour.*[3]	Morawski et al, 2018: Users were able to designate a "Medfriend" who was granted access to the person's medication taking history and received alerts when doses were missed and could provide peer support.[11]<br><br>Santo et al, 2018: The app included the ability to export and share information with others such as family members and the ability to alert them if the participant missed a dose.[10]
Habit formation	*Prompt rehearsal and repetition of the behaviour in the same context repeatedly so that the context elicits the behaviour.*[3]	Mira et al 2014: alerts and reminders encouraged participants to put healthy habits into practice, e.g. the association of taking their medicines with particular meals or daily activities.[2]<br><br>Santo et al, 2018: Scheduled reminders occurred up to three times at 10 minute intervals.[10]

For peer review only - http://bmjopen.bmj.com/site/about/guidelines.xhtml

Goal setting of behaviour	*Set or agree a goal defined in terms of the behaviour to be achieved.*[3]	Mira et al, 2014: The ALICE app included a function for the participant to store details of their prescriptions and related instructions.[2]<br><br>Morawski et al, 2018: Users were able to enter their medication lists manually, along with their preferred timing of administration.[11]

For peer review only

References

1 Shah V, Dileep A, Dickens C, *et al.* Patient-Centered Tablet Application for Improving Medication Adherence after a Drug-Eluting Stent. *Front public Heal* 2016;**4**:272. doi:10.3389/fpubh.2016.00272

2 Mira JJ, Navarro I, Botella F, *et al.* A Spanish pillbox app for elderly patients taking multiple medications: randomized controlled trial. *J Med Internet Res* 2014;**16**:e99. doi:10.2196/jmir.3269

3 Michie S, Richardson M, Johnston M, *et al.* The Behavior Change Technique Taxonomy (v1) of 93 Hierarchically Clustered Techniques: Building an International Consensus for the Reporting of Behavior Change Interventions. *Ann Behav Med* 2013;**46**:81–95. doi:10.1007/s12160-013-9486-6

4 Lakshminarayana R, Wang D, Burn D, *et al.* Using a smartphone-based self-management platform to support medication adherence and clinical consultation in Parkinson ' s disease. *NPJ Park Dis* 2017;**3**:1–8. doi:10.1038/s41531-016-0003-z

5 Kassavou A, Sutton S. Automated telecommunication interventions to promote adherence to cardio-metabolic medications: meta-analysis of effectiveness and meta-regression of behaviour change techniques. *Health Psychol Rev* 2018;**12**:25–42. doi:10.1080/17437199.2017.1365617

6 Santo K, Singleton A, Rogers K, *et al.* Medication reminder applications to improve adherence in coronary heart disease: a randomised clinical trial. *Heart* 2018;:heartjnl-2018-313479. doi:10.1136/heartjnl-2018-313479

7 Hammonds T, Rickert K, Goldstein C, *et al.* Adherence to Antidepressant Medications: A Randomized Controlled Trial of Medication Reminding in

For peer review only - http://bmjopen.bmj.com/site/about/guidelines.xhtml

College Students. 2015;**8481**. doi:10.1080/07448481.2014.975716

8 Labovitz DL, Shafner L, Gil MR, *et al.* Using Artificial Intelligence to Reduce the Risk of Nonadherence in Patients on Anticoagulation Therapy. *Stroke* 2017;**48**:1416–9. doi:10.1161/STROKEAHA.116.016281

9 Lakshminarayana R, Wang D, Burn D, *et al.* and adherence tools to manage Parkinson ' s disease (SMART-PD): study protocol for a randomised controlled trial (v7 ; 15 August 2014). 2014;:1–11.

10 Santo K, Chow CK, Thiagalingam A, *et al.* MEDication reminder APPs to improve medication adherence in Coronary Heart Disease (MedApp-CHD) Study : a randomised controlled trial protocol. 2017;:1–10. doi:10.1136/bmjopen-2017-017540

11 Morawski K, Ghazinouri R, Krumme A, *et al.* Association of a Smartphone Application With Medication Adherence and Blood Pressure Control. *JAMA Intern Med* 2018;**178**:802. doi:10.1001/jamainternmed.2018.0447

For peer review only

For peer review only - http://bmjopen.bmj.com/site/about/guidelines.xhtml

PRISMA 2009 Checklist

Section/topic	#	Checklist item	Reported on page #
**TITLE**			
Title	1	Identify the report as a systematic review, meta-analysis, or both.	1
**ABSTRACT**			
Structured summary	2	Provide a structured summary including, as applicable: background; objectives; data sources; study eligibility criteria, participants, and interventions; study appraisal and synthesis methods; results; limitations; conclusions and implications of key findings; systematic review registration number.	2-3
**INTRODUCTION**			
Rationale	3	Describe the rationale for the review in the context of what is already known.	4-5
Objectives	4	Provide an explicit statement of questions being addressed with reference to participants, interventions, comparisons, outcomes, and study design (PICOS).	6
**METHODS**			
Protocol and registration	5	Indicate if a review protocol exists, if and where it can be accessed (e.g., Web address), and, if available, provide registration information including registration number.	5
Eligibility criteria	6	Specify study characteristics (e.g., PICOS, length of follow-up) and report characteristics (e.g., years considered, language, publication status) used as criteria for eligibility, giving rationale.	6
Information sources	7	Describe all information sources (e.g., databases with dates of coverage, contact with study authors to identify additional studies) in the search and date last searched.	5
Search	8	Present full electronic search strategy for at least one database, including any limits used, such that it could be repeated.	Supplementary file p 2
Study selection	9	State the process for selecting studies (i.e., screening, eligibility, included in systematic review, and, if applicable, included in the meta-analysis).	6
Data collection process	10	Describe method of data extraction from reports (e.g., piloted forms, independently, in duplicate) and any processes for obtaining and confirming data from investigators.	6-7
Data items	11	List and define all variables for which data were sought (e.g., PICOS, funding sources) and any assumptions and simplifications made.	6-8 and Table 1, p21
Risk of bias in individual studies	12	Describe methods used for assessing risk of bias of individual studies (including specification of whether this was done at the study or outcome level), and how this information is to be used in any data synthesis.	7
Summary measures	13	State the principal summary measures (e.g., risk ratio, difference in means).	7
Synthesis of results	14	Describe the methods of handling data and combining results of studies, if done, including measures of consistency (e.g., I^2) for each meta-analysis.	7-8

For peer review only

For peer review only - http://bmjopen.bmj.com/site/about/guidelines.xhtml

PRISMA 2009 Checklist

Section/topic	#	Checklist item	Reported on page #
Risk of bias across studies	15	Specify any assessment of risk of bias that may affect the cumulative evidence (e.g., publication bias, selective reporting within studies).	7
Additional analyses	16	Describe methods of additional analyses (e.g., sensitivity or subgroup analyses, meta-regression), if done, indicating which were pre-specified.	7
**RESULTS**			
Study selection	17	Give numbers of studies screened, assessed for eligibility, and included in the review, with reasons for exclusions at each stage, ideally with a flow diagram.	8
Study characteristics	18	For each study, present characteristics for which data were extracted (e.g., study size, PICOS, follow-up period) and provide the citations.	P8-9 and Table 1, p 21
Risk of bias within studies	19	Present data on risk of bias of each study and, if available, any outcome level assessment (see item 12).	P10 and Table 2, p 23
Results of individual studies	20	For all outcomes considered (benefits or harms), present, for each study: (a) simple summary data for each intervention group (b) effect estimates and confidence intervals, ideally with a forest plot.	Figure 2, (and eTable 1 for weighting of each study and eTable 2 for raw extracted data found in supplementary file, p 4-5)
Synthesis of results	21	Present results of each meta-analysis done, including confidence intervals and measures of consistency.	9
Risk of bias across studies	22	Present results of any assessment of risk of bias across studies (see Item 15).	11 and efigure 1 on p 3 of supplementary material file
Additional analysis	23	Give results of additional analyses, if done (e.g., sensitivity or subgroup analyses, meta-regression [see Item 16]).	11
**DISCUSSION**			

For peer review only - http://bmjopen.bmj.com/site/about/guidelines.xhtml

PRISMA 2009 Checklist

Summary of evidence	24	Summarize the main findings including the strength of evidence for each main outcome; consider their relevance to key groups (e.g., healthcare providers, users, and policy makers).	11
Limitations	25	Discuss limitations at study and outcome level (e.g., risk of bias), and at review-level (e.g., incomplete retrieval of identified research, reporting bias).	13
Conclusions	26	Provide a general interpretation of the results in the context of other evidence, and implications for future research.	14
**FUNDING**			
Funding	27	Describe sources of funding for the systematic review and other support (e.g., supply of data); role of funders for the systematic review.	19

From: Moher D, Liberati A, Tetzlaff J, Altman DG, The PRISMA Group (2009). Preferred Reporting Items for Systematic Reviews and Meta-Analyses: The PRISMA Statement. PLoS Med 6(7): e1000097. doi:10.1371/journal.pmed1000097

For more information, visit: **www.prisma-statement.org**.

For peer review only

For peer review only - http://bmjopen.bmj.com/site/about/guidelines.xhtml

BMJ Open

Do mobile device apps designed to support medication adherence demonstrate efficacy? A systematic review of randomised controlled trials, with meta-analysis

Journal:	*BMJ Open*
Manuscript ID	bmjopen-2019-032045.R3
Article Type:	Original research
Date Submitted by the Author:	22-Dec-2019
Complete List of Authors:	Armitage, Laura; University of Oxford, Nuffield Department of Primary Care Health Sciences;<br>Kassavou, Aikaterini; University of Cambridge, Public Health and Primary Care ; University of Cambridge,<br>Sutton, Stephen; University of Cambridge,
**Primary Subject Heading**:	Public health
Secondary Subject Heading:	General practice / Family practice, Patient-centred medicine
Keywords:	Telemedicine < BIOTECHNOLOGY & BIOINFORMATICS, PRIMARY CARE, PUBLIC HEALTH, World Wide Web technology < BIOTECHNOLOGY & BIOINFORMATICS, GENERAL MEDICINE (see Internal Medicine)

SCHOLARONE™ Manuscripts

I, the Submitting Author has the right to grant and does grant on behalf of all authors of the Work (as defined in the below author licence), an exclusive licence and/or a non-exclusive licence for contributions from authors who are: i) UK Crown employees; ii) where BMJ has agreed a CC-BY licence shall apply, and/or iii) in accordance with the terms applicable for US Federal Government officers or employees acting as part of their official duties; on a worldwide, perpetual, irrevocable, royalty-free basis to BMJ Publishing Group Ltd ("BMJ") its licensees and where the relevant Journal is co-owned by BMJ to the co-owners of the Journal, to publish the Work in this journal and any other BMJ products and to exploit all rights, as set out in our licence.

The Submitting Author accepts and understands that any supply made under these terms is made by BMJ to the Submitting Author unless you are acting as an employee on behalf of your employer or a postgraduate student of an affiliated institution which is paying any applicable article publishing charge ("APC") for Open Access articles. Where the Submitting Author wishes to make the Work available on an Open Access basis (and intends to pay the relevant APC), the terms of reuse of such Open Access shall be governed by a Creative Commons licence – details of these licences and which Creative Commons licence will apply to this Work are set out in our licence referred to above.

Other than as permitted in any relevant BMJ Author's Self Archiving Policies, I confirm this Work has not been accepted for publication elsewhere, is not being considered for publication elsewhere and does not duplicate material already published. I confirm all authors consent to publication of this Work and authorise the granting of this licence.

For peer review only

Title

Do mobile device apps designed to support medication adherence demonstrate efficacy? A systematic review of randomised controlled trials, with meta-analysis

Word Count of Manuscript Text: 3522

Author Details:

Laura Armitage, MRCGP[1,2,3]

Aikaterini Kassavou, PhD[1]

Stephen Sutton, PhD[1]

Affiliations:

1. Department of Public Health and Primary Care, University of Cambridge, UK
2. National Institute for Health Research, Collaboration for Leadership in Applied Health Research and Care, East of England, UK
3. Nuffield Department of Primary Care Health Sciences, University of Oxford, UK

Author for Correspondence:

Laura Armitage

Email: laura.armitage@phc.ox.ac.uk Tel: +447791366355

Nuffield Department of Primary Care Health Sciences,

Radcliffe Primary Care Building, Radcliffe Observatory Quarter,

Woodstock Road,

Oxford,

OX2 6GG

For peer review only

ABSTRACT

Objectives

To estimate the efficacy of app-based interventions designed to support medication adherence and investigate which behaviour change techniques (BCTs) utilised by apps are associated with efficacy.

Design

Systematic review of randomised controlled trials (RCTs), with meta-analysis.

Setting

Medline/PubMed, PsycINFO, CINAHL, Embase and Web of Science were searched from 1990 to November 2018 for RCTs conducted in any health care setting.

Participants

Studies of participants of any age, taking prescribed medication for any health condition, for any duration.

Intervention

An app-based intervention delivered through a smartphone, tablet computer or personal digital assistant to help, support or advise about medication adherence.

Comparator

One of (i) usual care; (ii) a control app which did not use any BCTs to improve medication adherence or (iii) a non-app based comparator.

Primary and Secondary Outcome Measures

For peer review only

For peer review only - http://bmjopen.bmj.com/site/about/guidelines.xhtml

The primary outcome was the pooled effect size of changes in medication adherence. The secondary outcome was an association between BCTs utilised by apps and effect size.

Results

The initial search identified 13,259 citations. After title and abstract screening, full text articles of 83 studies were screened for eligibility. Nine RCTs with 1159 recruited participants were included. Mean age of participants was >50 years in all but one study. Health conditions of target populations included cardiovascular disease, depression, Parkinson's disease, psoriasis and multimorbidity. Meta-analysis indicated that patients who use mobile apps to support medication taking are more likely to self-report adherence to medications (OR 2.120, 95% CI 1.635-2.747, N=988) than those in comparator groups. Meta-regression of the BCTs did not reveal any significant associations with effect size.

Conclusions

App-based medication adherence interventions may have a positive effect on patient adherence. Larger scale studies are required to further evaluate this effect, including long-term sustainability, and intervention and participant characteristics that are associated with efficacy and app usage.

Article Summary – Strengths and Limitations of this Study

- This systematic review and meta-analysis of randomised controlled trials offers new information on the efficacy of mobile device apps to support medication adherence across a range of medical conditions.
- This review is reported according to the PRISMA Statement.

For peer review only - http://bmjopen.bmj.com/site/about/guidelines.xhtml

- A sensitive search strategy was designed and screening of articles and data extraction was performed independently by one clinical researcher and one non-clinical researcher.
- The review protocol was prospectively registered and the scope of the review, inclusion and exclusion criteria remained unchanged throughout.
- Two reviewers independently considered abstracts and full text articles for inclusion and extracted data and assessed included studies for risk of bias in the same way.

For peer review only

For peer review only - http://bmjopen.bmj.com/site/about/guidelines.xhtml

MANUSCRIPT TEXT

Introduction

Adherence to medication is defined as the extent to which patients take medications as prescribed and agreed with their health care providers.[1] The therapeutic benefit of prescribed medications is limited owing to an estimated 50% of patients not adhering to medications.[2] The annual cost of non-adherence is estimated to exceed £930 million in England[3] and between $100 and $300 billion in the US.[4] Furthermore, in 2003 the Adherence to Long-term Therapy report published by the World Health Organisation stated that unless medication adherence is addressed, advances in biomedical technology will not achieve their potential.[5]

Interventions for improving adherence should be reliable, contemporary, acceptable and readily available to the person. Mobile devices can meet these needs and in 2018, Ofcom reported that 78% of adults and 95% of 16-24 year olds in the UK own a smartphone, demonstrating that smartphones are a growing part of modern life.[6] Patients who are prescribed multiple medications are commonly in the older age group, where the percentage of smartphone users may be lower. However, smartphone ownership in those aged over 65 more than trebled between the years 2012 and 2015 from 5% to 18%, and is set to continue to increase. [7]

Hundreds of apps are now available to patients to support them in taking regular medication.[8] Such apps frequently utilise behaviour change techniques (BCTs) to promote improvements in adherence. A BCT is defined as an active component of an intervention that is designed to change behaviour.[9] To date, the efficacy of smartphone apps to support patient adherence to regular prescribed medications has not been evaluated within the scientific literature using rigorous quantitative review. We have performed a novel systematic review with meta-analysis to evaluate the efficacy of smartphone apps in supporting medication adherence.

For peer review only - http://bmjopen.bmj.com/site/about/guidelines.xhtml

The objectives of this review are (i) to establish whether apps designed to support medication adherence demonstrate efficacy and (ii) to identify the intervention characteristics and Behaviour Change Techniques (BCTs) associated with efficacy. The conclusions drawn have potential to inform future large-scale public health interventions for the improvement of medication adherence.

METHODS

This systematic review is reported according to the PRISMA statement.[10] The protocol for the review is registered with and published on the PROSPERO database of systematic reviews (registration number: CRD42017080150).[11] The scope of the review, inclusion and exclusion criteria remained unchanged throughout.

Patient and Public Involvement

This research was done without patient involvement. Patients were not invited to comment on the study design and were not consulted to develop patient relevant outcomes or interpret the results. Patients were not invited to contribute to the writing or editing of this document for readability or accuracy.

Data Sources

Medline/PubMed, PsycINFO, CINAHL, Embase and Web of Science electronic databases were searched from 1990 to November 2018. A sensitive search strategy was designed based on preliminary searches, relevant papers and key words. Key search terms included adherence, non-adherence, smart phone app and randomised control trials (RCTs). An example detailed search strategy for the Medline/PubMed database can be found in supplementary information, (eText 1). Reference lists of included studies were screened for additional relevant trials.

For peer review only - http://bmjopen.bmj.com/site/about/guidelines.xhtml

Two reviewers (LA and AK) independently screened all citations by title and abstract and excluded those which were irrelevant or clearly did not meet eligibility criteria. Full text articles were obtained for all remaining citations and screened against the eligibility criteria by the same reviewers. Any discrepancies at any stage in the screening process were settled through discussion between LA, AK and a third reviewer (SS).

Study Selection

RCTs relevant to this review were those that investigated the use of an app on a mobile device to support medication adherence. Inclusion criteria were as follows:

(i) Participants of any age who were taking one or more prescribed medications for any health condition, for any duration.

(ii) An intervention group which received an app-based intervention delivered through a smartphone, tablet computer or personal digital assistant to help, support or advise about medication adherence.

(iii) A comparator group which received usual care, a control condition which did not utilise an app,[12] or a control app which did not include any BCTs (for example an app that was a non-health related game[13]).

(iv) Medication adherence data reported for both the intervention and comparator groups.

Exclusion criteria were as follows:

(i) Complex interventions of which an app was just one component.

(ii) Interventions delivered by text messaging or telemedicine.

(iii) Outcome assessed was adherence to treatment components other than prescribed medication. For example one study reported outcome data as a composite score, combining adherence to other aspects of the medical regimen (such as clinic attendance and laboratory work) with adherence to medication.[14]

For peer review only - http://bmjopen.bmj.com/site/about/guidelines.xhtml

(iv) A study population of healthy recruits who were required to adhere to a placebo medication.

(v) Comparator group received an adherence intervention in another format, such as a different medication adherence app.

Published articles were required to be in English language.

Data Extraction and synthesis

Data were extracted independently by two reviewers (LA and AK) using a custom data extraction form. Participant baseline characteristics were extracted, including age and sex. Raw data on medication adherence were extracted either from the published articles or by contacting the authors of the primary studies by e-mail. When two measures of adherence were reported, that which was considered most comparable to the outcome measures of the other included studies was used for meta-analysis. For example, one study reported adherence according to both the number of times an electronically chipped dispenser was actioned by the patient and the weight of the dispensing cannister at study follow up, and the reviewers agreed that the action of dispensing was most comparable to that of the other included studies.[15] Authors were contacted by email for any other data required which were not available in the published articles. Where more than one app intervention was tested, data for the intervention considered most comparable to the other studies was extracted for meta-analysis. For example, one study investigated a 'basic app' (which provided a uni-directional once daily reminder) as well as an 'advanced app' (which provided customisable and interactive features) versus usual care.[16] The reviewers agreed that the advanced app was most comparable to the interventions of the other studies and so data pertaining to this intervention were extracted. BCTs that were utilised in the app-based interventions were coded according to the Behaviour Change Taxonomy.[17] This is a structured taxonomy of techniques utilised by interventions to change behaviour. The BCT Taxonomy does not have a code relating to performance of behaviour and therefore where participants were required to enter whether or not

For peer review only - http://bmjopen.bmj.com/site/about/guidelines.xhtml

medication was taken, this was coded as ‘Report whether or not the behaviour was performed’. Similarly, the Taxonomy does not have a code relating to tailoring of an intervention and therefore where a study used information gained about a person to define the content, frequency or format of an intervention, this was coded as a ‘Tailored’ intervention.[18]

The included trials were assessed for risk of bias using the Cochrane Risk of Bias Tool.[19] Two reviewers (LA and AK) individually assessed each of the included articles against criteria outlined in the tool. Any discrepancies at any stage in the coding process were settled through discussion between the three reviewers (LA, AK, SS). Studies were considered at unclear risk of bias for an item when the primary article did not provide sufficient information for the reviewers to decide categorically between high or low risk of bias.

Statistical Analysis

Outcome data on medication adherence were extracted from each of the included studies at the final observation point. Authors were contacted for raw final observation point adherence data if these was not reported in published manuscripts. Trials were considered homogeneous based on the content of the intervention and comparator groups and the outcome measurement of medication adherence. Comprehensive Meta-Analysis software[20] was used to estimate the overall effect of app-delivered interventions on medication adherence using a random-effects model and to perform meta-regression using the BCTs utilised by the apps. Comprehensive Meta-analysis Software conversion function was used where required to convert extracted raw data into odds ratio and standardised mean difference between the intervention and control groups.[21] The I^2 statistic was used to estimate heterogeneity between included studies.

RESULTS

For peer review only - http://bmjopen.bmj.com/site/about/guidelines.xhtml

The initial electronic database search identified 13 259 potentially eligible studies, and two were identified from reference lists of identified articles. After removal of duplicates, 9971 citations were screened by title and abstract. We considered 83 of these to be eligible and retrieved the full-text articles. Of these, nine studies reported outcome data eligible for meta-analysis[12,13,15,16,22–26]. The screening process and reasons for exclusion are reported in Figure 1.

Study characteristics of the nine studies are displayed in Table 1. Publication year of included studies ranged from 2014-2018. The median sample size was 102 participants (range 24-412). The median follow-up period was 12 weeks (range 28 days to 16 weeks).

Mean age of participants in the intervention group was reported by 8 studies and ranged from 20.3 (SD 4.0)[22] to 73.8 (SD 8)[12]; the mean of the mean ages across these 8 studies was 56.7 years. One did not report the mean age but presented the percentage of participants whose age fell within a series of bandwidths.[15] The percentage of participants in intervention groups who were female ranged from 11%[16] to 89%[22], and the mean percentage of females among the included studies was 45.2%.

Health problem of the target population varied between studies. Five studies sampled a patient population with cardiovascular disease[12,13,16,23,26] and each of the remaining four studies enrolled patient populations with different health problems: depression[22], Parkinson's disease,[24] psoriasis[15] and multimorbidity.[25]

Outcome Measurement Methods

A number of measures of medication adherence were utilised in the included studies. Of the 9 studies, four measured adherence using the 8-Item Morisky Medication Adherence Scale[24,26,27], with one of these also measuring adherence through a medication possession ratio (see Supplementary Material file, Shah et al, 2016).[13] For this study, the Morisky Medication Adherence Scale data were extracted for the meta-analysis as this was most comparable to the data

For peer review only - http://bmjopen.bmj.com/site/about/guidelines.xhtml

available for other studies. A further study measured adherence using the 4-Item Morisky Medication Adherence Scale.[25] Of the remaining studies, two reported adherence according to pill count[22,23], one the A14 self-report scale of medication adherence[12] and one through a digital chip record fitted within the medication dispenser (Table 1).[15]

Meta-analysis

Meta-analysis was performed, pooling results from the nine eligible studies which reported data on 988 participants, to estimate the effect of mobile apps to improve medication adherence.[12,13,15,16,22–26] The result of this meta-analysis indicated that patients who participated in medication adherence interventions delivered by mobile apps were more likely to adhere to prescribed medications (OR 2.120, 95% CI 1.635-2.747) than those that did not use such interventions. The I^2 value was 9.8 indicating low statistical heterogeneity between the studies included in the meta-analysis. The forest plot of the meta-analysis can be seen in Figure 2. The weighting of each individual study in the meta-analysis can be seen in eTable 1, and raw data extracted for the meta-analysis can be found in eTable 2.

A focussed meta-analysis was performed, pooling results from the 5 studies which reported medication adherence according to the Morisky Medication Adherence Scale.[13,16,24–26] The result of this also indicated that patients using medication adherence interventions delivered by mobile apps reported higher adherence to prescribed medications (OR 1.83, 95% CI 1.42-2.36).

Behaviour Change Techniques

The most common BCT utilised by apps was 'Tailored", which was utilised by all studies. The next most common was 'Prompts and Cues', utilised by seven studies[12,15,16,22–25] and 'Report whether or not the behaviour was performed' which was utilised by six studies.[12,16,22–25] Information on the BCTs coded for each of the interventions and definitions of each of the BCTs is available in eTables 3 and 4. Agreement between reviewers for classification of BCTs utilised by the

For peer review only - http://bmjopen.bmj.com/site/about/guidelines.xhtml

interventions was assessed using the kappa statistic. This revealed a 'very good' level of agreement (kappa = 0.902, 95% CI 0.835 – 0.969). All BCTs coded for each of the study interventions are provided in eTable3. Those coded in more than three but less than 6 of the 9 studies were included in the meta-regression; this did not reveal any significant associations between the BCTs utilised and effect size.

Risk of bias at primary study level

A summary of the risk of bias analysis can be seen in Table 2**.** Overall, all studies were deemed as unclear in their risk of bias.

The most common source of risk of bias amongst the studies was the absence of blinding of participants and personnel to the outcome measure. The second most common risk of bias arose from a lack of objective measurements of medication adherence. This was due to the use of self-report measures of adherence: six of the studies included in the meta-analysis reported adherence based on a self-assessment scale[12,13,16,24–26], and one study each used patient-entered medication logs in an app[22], an electronically chipped medicated foam dispenser[15] and pill counts at clinic visits.[23]

Self-assessment scales are a form of self-report and therefore are a source of potential bias, including those of social desirability, and those arising from selective recall and the duration of the recall period.[28] Two studies included in this review, which measured adherence through participant self-report, sought to compare their subjective measurements of adherence with serum measurements of either drug-reactivity units or a medication's anticoagulant effect.[13,23] One study showed that serum measurement of the medication's anticoagulant effect was similar between both the intervention and comparator groups, despite adherence measures being better in the intervention group.[23] The other study revealed that drug-reactivity units were worse in the

For peer review only - http://bmjopen.bmj.com/site/about/guidelines.xhtml

intervention group than the control group, which conflicted with measured adherence outcome data.[13] However, the presence of a drug biomarker does not equate to compliance and the absence does not equate to non-compliance.[29] Indeed, drug metabolism should be taken into account for such measures of medication adherence, as physiological state and metabolic rate vary among individuals.[30]

Publication Bias

A funnel plot of effect size estimates of each of the studies included in the meta-analysis is provided in the online supplementary file (eFigure 1). Formal analysis of plot symmetry was not performed owing to the total number of studies included being less than 10.[31] Visual inspection reveals moderate symmetry of the plot.

DISCUSSION

Principal Findings

We conducted a rigorous systematic review of the literature to investigate whether mobile device apps demonstrate efficacy in supporting medication adherence. A random-effects meta-analysis of the pooled results from all nine studies indicated that people who use medication reminder apps are significantly more likely to adhere to their medications than those who do not, but the results should be interpreted with caution owing to data for 6 of the 9 studies included in the meta-analysis being based on self-reported measures of adherence.

Intervention effects reported between the studies varied, as shown in the forest plot (Figure 2). All nine studies reported that their mobile app-delivered interventions demonstrated efficacy at supporting medication adherence, although in five studies the interventions did not have a statistically significant effect.[12,13,16,22,25] Although the I^2 measure of statistical heterogeneity was low, and meta-regression of the BCTs utilised by the studies was not able to account for variance in effect observed between studies, there are other potential sources of methodological

For peer review only - http://bmjopen.bmj.com/site/about/guidelines.xhtml

and clinical heterogeneity which may account for this. One potential explanation might be the variation in adherence measurement. This may be illustrated by three studies which incorporated five of the same BCTs[23–25] but where one[23] reported better medication adherence (OR 10.633, 95% CI 2.339-48.344) than the other two (OR 1.946, 95% CI 1.094-3.461 and OR 1.251, 95% CI 0.612-2.559).[24,25] In Labovitz et al's study, the medication adherence was measured by pill counts at clinic follow up visits[23], whereas in the other two studies, adherence was measured by retrospective self-scoring on a medication adherence scale[24,25] It could be argued that both of these different measures of adherence are vulnerable to bias with pill counts potentially being vulnerable to expectation bias and retrospective self-scores being vulnerable to recall bias. We subsequently performed a focused meta-analysis of the five studies which measured adherence using the Morisky Medication Adherence Scale to investigate whether this influenced the effect size observed. Whilst the result of meta-analysis remained significant, the effect size was lower. This may support the use of real-time or prospective adherence monitoring over retrospective self-report for future research in the field of medication adherence.

A further possible explanation for variance in effect size might be techniques, beyond those reported in the BCT taxonomy, utilised by the apps. For example, in the study by Labovitz et al, the app intervention employed artificial intelligence to identify the participant, their medication and ingestion of the medication, using the camera of the mobile device and software algorithms. This could be regarded as a highly tailored intervention and one might hypothesise that the degree of tailoring could influence the effect size observed. A further example of a highly tailored intervention was seen in the study by Lakshminarayana et al. All of the app-based interventions in the included studies were tailored to the prescribed medication regimen, but in this instance the tailoring went beyond the prescribed medication regimen, to include tailoring to the patients' intentional and non-intentional non-adherence, beliefs about medication taking, mood, cognitive impairment, symptom control, quality of life, age and disease duration. A significant effect size was observed in this study,

For peer review only - http://bmjopen.bmj.com/site/about/guidelines.xhtml

and this may support a hypothesis that highly tailored apps are more effective at supporting medication adherence, than minimally tailored apps.

Further factors which may have contributed to variance in the effect size seen between studies relate to the participants, including disease burden, non-adherence behaviours, perceived benefit from the medication and any side effects. Interpretation of the overall result of the meta-analysis and generalisability to the wider population should therefore be cautious.

Strengths and Weaknesses of the Primary Studies

Based on the assessment of the risk of bias undertaken as part of this review, the evidence included in this meta-analysis is of moderate quality. The most common weaknesses in this evidence are small sample size, lack of objectivity of the outcome measurement, a lack of blinding of participants and personnel and limited follow up time.

Strengths and Weaknesses at Review Level

Our comprehensive literature search and search of the PROSPERO Database indicate this is the first systematic review and meta-analysis to investigate the efficacy of mobile apps to support medication adherence. This offers new knowledge to the field; however, as such there are no comparable studies to which the results of this review can be compared.

The pooling of data from a group of studies which used a variety of methods for measurement of medication adherence and targeted at patients with different healthcare problems is a limitation of this review. As further studies in this field are published it will become more feasible to conduct subgroup analysis or meta-regression analyses against variables such as age, health condition, targeted medication, intervention content and app characteristics. A standardised protocol for measuring adherence and reporting the content and characteristics of app interventions may facilitate further analysis of the latter two variables.

For peer review only - http://bmjopen.bmj.com/site/about/guidelines.xhtml

This review cannot report on the likely sustainability of the effect on medication adherence given the short study duration of the studies included in this review; across the 9 studies, the maximum follow up period was 16 weeks. Furthermore, the optimal frequency and duration of app use that is required to achieve improvements in medication adherence is not known. Long term and large-scale research is required in this field to addresses the question regarding sustainability of the observed effect found in this meta-analysis.

This systematic review and meta-analysis included studies with a range of ages of participants. The lowest mean age of participants in the included studies was 20.9 years and the highest was 70.9 years. The ubiquity and use of mobile devices varies across age groups[7], and whilst the majority of participants in these studies were aged 50 and over, further research to establish the acceptability and usability of adherence apps among different age groups adults would be valuable.

Finally, the reviewers set out to perform a meta-regression of the BCTs utilised by app interventions in the included studies.[11] However, this did not reveal any significant association between the BCTs utilised and observed effect size. This may be owing to the low estimate of heterogeneity and the small number of eligible included studies.

Conclusions

This meta-analysis indicates that medication adherence interventions delivered by smartphone apps are associated with higher levels of self-reported adherence to prescribed medications. However, when considering whether to recommend an app to patients, these results should be interpreted with caution owing to variance in effect size seen between the studies, the small sample sizes taken from different patient populations, heterogeneity among included studies and the prominence of self-reported scales to measure medication adherence. Further research should aim to first establish a standard protocol for the measuring and reporting of adherence in the context of evaluating digital

For peer review only - http://bmjopen.bmj.com/site/about/guidelines.xhtml

interventions. Subsequently, further research investigating intervention characteristics associated with an app's effect and the sustainability of any effect gained would add to this field.

.

For peer review only

For peer review only - http://bmjopen.bmj.com/site/about/guidelines.xhtml

References

1 Osterberg L, Blaschke T. Adherence to Medication. *N Engl J Med* 2005;**353**:487–97. doi:10.1056/NEJMra050100

2 Brown MT, Bussell JK. Medication Adherence: WHO Cares? *Mayo Clin Proc* 2011;**86**:304. doi:10.4065/MCP.2010.0575

3 Elliott RA, Boyd MJ, Waring J, *et al.* Understanding & evaluating the New Medicines Service in the NHS in England (029/0124). 2014. http://www.nottingham.ac.uk/~pazmjb/nms/downloads/report/files/assets/common/downloads/108842 A4 Main Report.v4.pdf (accessed 12 Feb 2018).

4 Iuga AO, McGuire MJ. Adherence and health care costs. *Risk Manag Healthc Policy* 2014;**7**:35–44. doi:10.2147/RMHP.S19801

5 Sabaté E. Evidence for action. *World Heal Organ* Published Online First: 2003.http://www.who.int/chp/knowledge/publications/adherence_introduction.pdf (accessed 12 Feb 2018).

6 Ofcom. Communications Market Report. 2018. https://www.ofcom.org.uk/__data/assets/pdf_file/0022/117256/CMR-2018-narrative-report.pdf (accessed 3 Aug 2018).

7 Ofcom. The Communications Market Report. 2015. https://www.ofcom.org.uk/__data/assets/pdf_file/0022/20668/cmr_uk_2015.pdf (accessed 12 Feb 2018).

8 Ahmed I, Ahmad NS, Ali S, *et al.* Medication Adherence Apps: Review and Content Analysis. *JMIR mHealth uHealth* 2018;**6**:e62. doi:10.2196/mhealth.6432

For peer review only

For peer review only - http://bmjopen.bmj.com/site/about/guidelines.xhtml

9 Wideman TH, Sullivan MJL, Inada S, *et al.* Behaviour Change Techniques. In: Gellman Marc D., Turner J. Rick., eds. *Encyclopedia of Behavioral Medicine*. New York, NY: : Springer New York 2013. 12. doi:10.1007/978-1-4419-1005-9_1661

10 Moher D, Liberati A, Tetzlaff J, *et al.* Preferred Reporting Items for Systematic Reviews and Meta-Analyses: The PRISMA Statement. *PLoS Med* 2009;**6**:e1000097. doi:10.1371/journal.pmed.1000097

11 Armitage L, Kassavou A, Sutton S. A systematic review of the literature with met-analysis and meta-regression: the use of smartphone applications to support medication adherence/. PROSPERO Int. Prospect. Regist. Syst. Rev. 2017. doi:CRD42017080150

12 Mertens A, Brandl C, Miron-shatz T, *et al.* A mobile application improves therapy-adherence rates in elderly patients undergoing rehabilitation. *Medicine (Baltimore)* 2016;**95**:e4446.

13 Shah V, Dileep A, Dickens C, *et al.* Patient-Centered Tablet Application for Improving Medication Adherence after a Drug-Eluting Stent. *Front public Heal* 2016;**4**:272. doi:10.3389/fpubh.2016.00272

14 DeVito Dabbs A, Song MK, Myers BA, *et al.* A Randomized Controlled Trial of a Mobile Health Intervention to Promote Self-Management After Lung Transplantation. *Am J Transplant* 2016;**16**:2172–80. doi:10.1111/ajt.13701

15 Svendsen MT, Andersen F, Andersen KH, *et al.* A smartphone application supporting patients with psoriasis improves adherence to topical treatment: a randomized controlled trial. *Br J Dermatol* 2018;**179**:1062–71. doi:10.1111/bjd.16667

16 Santo K, Singleton A, Rogers K, *et al.* Medication reminder applications to improve adherence in coronary heart disease: a randomised clinical trial. *Heart* 2018;:heartjnl-2018-313479. doi:10.1136/heartjnl-2018-313479

For peer review only

For peer review only - http://bmjopen.bmj.com/site/about/guidelines.xhtml

17 Michie S, Richardson M, Johnston M, *et al.* The Behavior Change Technique Taxonomy (v1) of 93 Hierarchically Clustered Techniques: Building an International Consensus for the Reporting of Behavior Change Interventions. *Ann Behav Med* 2013;**46**:81–95. doi:10.1007/s12160-013-9486-6

18 Kassavou A, Sutton S. Automated telecommunication interventions to promote adherence to cardio-metabolic medications: meta-analysis of effectiveness and meta-regression of behaviour change techniques. *Health Psychol Rev* 2018;**12**:25–42. doi:10.1080/17437199.2017.1365617

19 Higgins JP, Altman DG, Sterne JA. Chapter 8: Assessing risk of bias in included studies. https://training.cochrane.org/handbook/chapter-08 (accessed 14 Feb 2018).

20 Comprehensive Meta-Analysis Software (CMA). https://www.meta-analysis.com/ (accessed 15 Feb 2018).

21 Borenstein M, Hedges L., Higgins PT, *et al. Introduction to Meta-Analysis, Converting Among Effect Sizes*. John Wiley and Sons 2009. https://www.meta-analysis.com/downloads/Meta-analysis Converting among effect sizes.pdf (accessed 8 Mar 2019).

22 Hammonds T, Rickert K, Goldstein C, *et al.* Adherence to Antidepressant Medications: A Randomized Controlled Trial of Medication Reminding in College Students. 2015;**8481**. doi:10.1080/07448481.2014.975716

23 Labovitz DL, Shafner L, Gil MR, *et al.* Using Artificial Intelligence to Reduce the Risk of Nonadherence in Patients on Anticoagulation Therapy. *Stroke* 2017;**48**:1416–9. doi:10.1161/STROKEAHA.116.016281

24 Lakshminarayana R, Wang D, Burn D, *et al.* Using a smartphone-based self-management platform to support medication adherence and clinical consultation in Parkinson ' s disease.

For peer review only - http://bmjopen.bmj.com/site/about/guidelines.xhtml

NPJ Park Dis 2017;**3**:1–8. doi:10.1038/s41531-016-0003-z

25 Mira JJ, Navarro I, Botella F, *et al.* A Spanish pillbox app for elderly patients taking multiple medications: randomized controlled trial. *J Med Internet Res* 2014;**16**:e99. doi:10.2196/jmir.3269

26 Morawski K, Ghazinouri R, Krumme A, *et al.* Association of a Smartphone Application With Medication Adherence and Blood Pressure Control. *JAMA Intern Med* 2018;**178**:802. doi:10.1001/jamainternmed.2018.0447

27 Santo K, Chow CK, Thiagalingam A, *et al.* MEDication reminder APPs to improve medication adherence in Coronary Heart Disease (MedApp-CHD) Study : a randomised controlled trial protocol. 2017;:1–10. doi:10.1136/bmjopen-2017-017540

28 Althubaiti A. Information bias in health research: definition, pitfalls, and adjustment methods. *J Multidiscip Healthc* 2016;**9**:211–7. doi:10.2147/JMDH.S104807

29 Farmer KC. Methods for measuring and monitoring medication regimen adherence in clinical trials and clinical practice. *Clin Ther* 1999;**21**:1074–90. doi:10.1016/S0149-2918(99)80026-5

30 Lam WY, Fresco P. Medication Adherence Measures: An Overview. *Biomed Res Int* 2015;**2015**:217047. doi:10.1155/2015/217047

31 Sterne JAC, Sutton AJ, Ioannidis JPA, *et al.* Recommendations for examining and interpreting funnel plot asymmetry in meta-analyses of randomised controlled trials. *BMJ* 2011;**343**:d4002. doi:10.1136/BMJ.D4002

Sources of funding

For peer review only - http://bmjopen.bmj.com/site/about/guidelines.xhtml

This paper presents independent research funded by the National Institute for Health Research (NIHR) under its Programme Grants for Applied Research Programme (Grant Reference Number RP-PG-0615-20013). The views expressed are those of the authors and not necessarily those of the NIHR or the Department of Health and Social Care. The National Institute for Health Research's (NIHR) Collaboration for Leadership in Applied Health Research and Care (CLAHRC) in East of England provided salary support to LA during conduction of this review. The funders had no role in review design, data collection, data analysis, data interpretation, the writing of the manuscript, and decision to submit the manuscript for publication.

Author Contributions

All authors were involved in design of the review and undertook methodological planning. LA performed and refined the searches in consultation with a medical librarian. LA and KK performed initial screening and data extraction and SS gave screening advice where any disagreements arose. All authors contributed to performing the meta-analysis to data interpretation. LA led the writing and all authors contributed to successive drafts and approved the final manuscript.

Data Availability

All data relevant to the study are included in the article or uploaded as supplementary information

Competing Interests

The authors have no competing interests to declare

Figure Legends

Figure 1. PRISMA Flowchart

Figure 2. Forest Plot of Randomised Trials evaluating a mobile device app to support medication adherence against comparators.

For peer review only

For peer review only

For peer review only - http://bmjopen.bmj.com/site/about/guidelines.xhtml

Table 1: Study Characteristics

Source (Sample size)	Study Nation	Study Design	Health Problem of Target Population	Recruitment setting	Participant characteristics at recruitment				App intervention	Outcome measure	Follow up interval
					Intervention group		Comparator group				
					Mean age (SD)	Female, N (%)	Mean age (SD)	Female, N (%)			
Hammonds et al, 2015 (57)	USA	RCT	Depression	College students were recruited via campus-based advertisements and the university research recruitment system	20.3 (4.0)	25 (83)	20.9 (4.7)	24 (89)	Medication reminder app	No. of participants taken ≥80% prescribed tablets per day.	25-35d
Labovitz et al, 2017 (28)	USA	RCT	Ischaemic stroke requiring secondary oral anti-coagulation	Outpatient clinic	58.3 (9.79)	6 (40)	55.5 (16.55)	9 (69)	Artificial intelligence app	Pill count	12w
Lakshmin-arayana et al, 2017 (215)	UK	RCT	Parkinson's disease	outpatient clinic	59.86 (9.13)	34 (36.2)	60.71 (10.26)	45 (42.1)	Parkinson's tracker app	Self-report: 8-Item MMAS	16w
Mertens et al, 2016 (24)	Germany	RCT Cross-over	Cardiovascular disease	Cardiac rehab groups	73.8[a]	12 (50)[a]	73.8[a]	12 (50)[a]	Medication Plan app (version 1.3)	Self-report: A14 scale	28d
Mira et al, 2014 (102)	Spain	RCT	Multi-comorbidity	Community health centres	70.9 (8)	21 (41)	72.9 (6)	23 (48)	ALICE medication self-management app	Self-report: 4-Item MMAS	3m

For peer review only - http://bmjopen.bmj.com/site/about/guidelines.xhtml

Morawski et al, 2018<br><br>(412)	USA	RCT	Hypertension	Social media, mobile apps, targeted advertisements and online communications	51.7 (10.5)		120 (57.4)	52.4 (10.1)		127 (62.9)	MediSAFE-BP app	Self-report: 8-Item MMAS	12w
Santo et al, 2018<br><br>(163)	Australia	RCT	Coronary Heart Disease	Inpatient, outpatient and rehabilitative care settings.	58.4 (9.04)		14 (13)	56.8 (8.64)		6 (11)	MedApp-CHD	Self-report: 8-Item MMAS	3m
Shah et al, 2016<br><br>(24)	USA	RCT	Cardiovascular stent	Patients in the immediate post-operative phase of insertion of a drug-eluting cardiovascular stent	60.5 (8.9)		6 (46)	60.6 (4.1)		4 (36)	MyIDEA education app	Medication possession ratio and Self-report: 8-Item MMAS	90d
Svendsen et al, 2018<br><br>(134)	Denmark	RCT	Psoriasis	Hospital posters and the dermatology outpatient clinic.	18-40<br>41-50<br>51-60<br>61-75	29[b]<br>21<br>26<br>24	27 (40)	18-40<br>41-50<br>51-60<br>61-75	32[b]<br>20<br>24<br>24	25 (38)	My Pso SmarTop Version 1.0 or Smartop number 0536776	Measured via a digital chip record in the medication dispenser.	28d

MMAS = Morisky Medication Adherence Scale.
[a]Baseline characteristics presented for whole cohort as study design was a randomised cross-over trial; [b]number of participants in each age group (years) presented as mean age not available.

Table 2: Risk of Bias Summary Table

For peer review only - http://bmjopen.bmj.com/site/about/guidelines.xhtml

Source	Allocation sequence adequately:		Free from baseline imbalance?			Blinding of participants and personnel and	Objective outcome assessment	Incomplete outcome data adequately addressed	Free from selective outcome reporting	Was the intervention independent of other bias
	Generated	Concealed	Age	Gender	Adherence					
Hammonds et al, 2015	🟡	🟡	🟢	🟢	🟢	🔴	🔴	🟢	🟢	🟢
Labovitz et al, 2017	🟡	🟡	🟡	🟡	🟡	🔴	🔴	🟢	🟢	🟢
Lakshminarayana et al, 2017	🟢	🟢	🟡	🟢	🟢	🔴	🟡	🟢	🟡	🟢
Mertens et al, 2016	🟡	🟡	🟡	🟡	🟡	🔴	🔴	🟢	🔴	🟡
Mira et al, 2014	🟡	🟡	🟡	🟢	🟢	🔴	🔴	🟡	🟢	🟢
Morawski et al, 2018	🟢	🟡	🟡	🟢	🟢	🟡	🔴	🟡	🟢	🟢
Santo et al, 2018	🟢	🟡	🟡	🟢	🟢	🔴	🔴	🟡	🟢	🟢
Shah et al, 2016	🟢	🟢	🟡	🟡	🟡	🟡	🟡	🟢	🟢	🟢
Svendsen et al, 2018	🟢	🟢	🟡	🟡	🟡	🔴	🟢	🟢	🟢	🟢

🟢 = Low risk of bias, 🟡 = Unclear risk of bias, 🔴 = High risk of bias

For peer review only

For peer review only - http://bmjopen.bmj.com/site/about/guidelines.xhtml

For peer review only

For peer review only - http://bmjopen.bmj.com/site/about/guidelines.xhtml

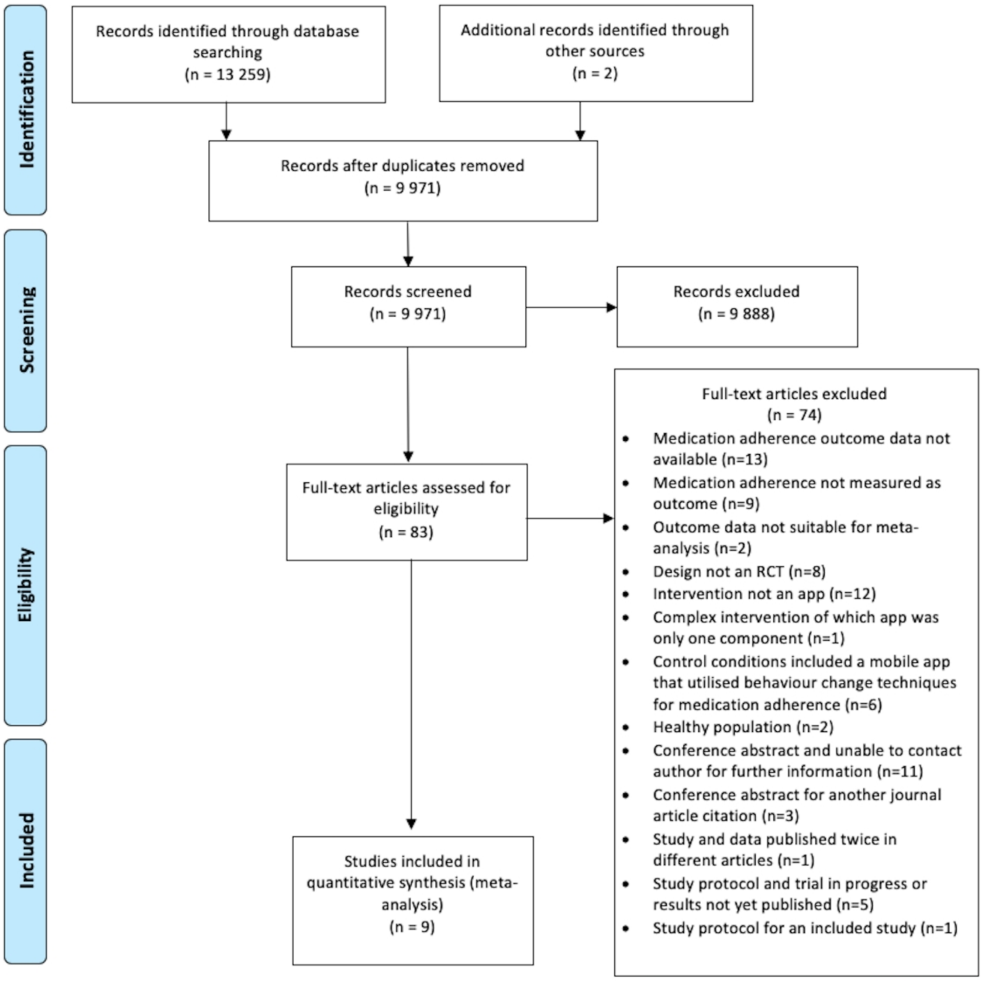

Figure 1. PRISMA Flowchart

Study name	Statistics for each study				
	Odds ratio	Lower limit	Upper limit	Z-Value	p-Value
Hammonds 2015	3.500	0.945	12.966	1.875	0.061
Labovitz 2017	10.633	2.339	48.344	3.060	0.002
Lakshminarayana 2017	1.946	1.094	3.461	2.266	0.023
Mertens 2016	2.975	0.675	13.117	1.440	0.150
Mira 2014	1.251	0.612	2.559	0.614	0.539
Morawski 2018	1.896	1.331	2.700	3.547	0.000
Shah 2016	2.206	0.505	9.626	1.052	0.293
Svendsen 2018	3.222	1.526	6.803	3.068	0.002
Santo 2018	2.029	0.991	4.154	1.934	0.053
	2.120	1.635	2.747	5.677	0.000

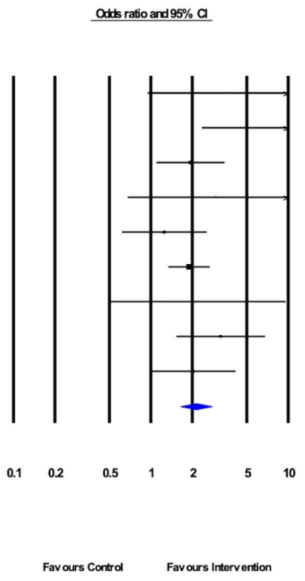

Figure 2. Forest Plot of Randomised Trials evaluating a mobile device app to support medication adherence against comparators.

SUPPLEMENTAL MATERIAL FILE

Mobile device apps to support medication adherence:

A systematic review of randomised controlled trials,

with meta-analysis

For peer review only

Author Details:

Laura Armitage, MRCGP

Aikaterini Kassavou, PhD

Stephen Sutton, PhD

For peer review only - http://bmjopen.bmj.com/site/about/guidelines.xhtml

eText 1: Medline/Pubmed Search Strategy

1. medication adherence.mp. or exp Patient Compliance/ or exp Medication Adherence/
2. nonnadherence.mp.
3. non-adherence.mp.
4. non adherence.mp.
5. concordance.mp.
6. concordant.mp.
7. 1 or 2 or 3 or 4 or 5 or 6
8. app.mp.
9. apps.mp.
10. application.mp.
11. exp Mobile Applications/ or mobile application*.mp.
12. smartphone.mp. or exp Smartphone/ or exp Cell Phones/
13. mobile*.mp.
14. phone*.mp.
15. 8 or 9 or 10 or 11 or 12 or 13 or 14
16. randomized controlled trial.mp. or exp Randomized Controlled Trial/
17. RCT.mp.
18. randomised.mp.
19. randomized.mp.
20. control*.mp.
21. 16 or 17 or 18 or 19 or 20
22. 7 and 15 and 21
23. limit 22 to (english language and yr="1990 - 2017")

For peer review only - http://bmjopen.bmj.com/site/about/guidelines.xhtml

eFigure 1: Funnel Plot of effect estimates from studies included in meta-analysis

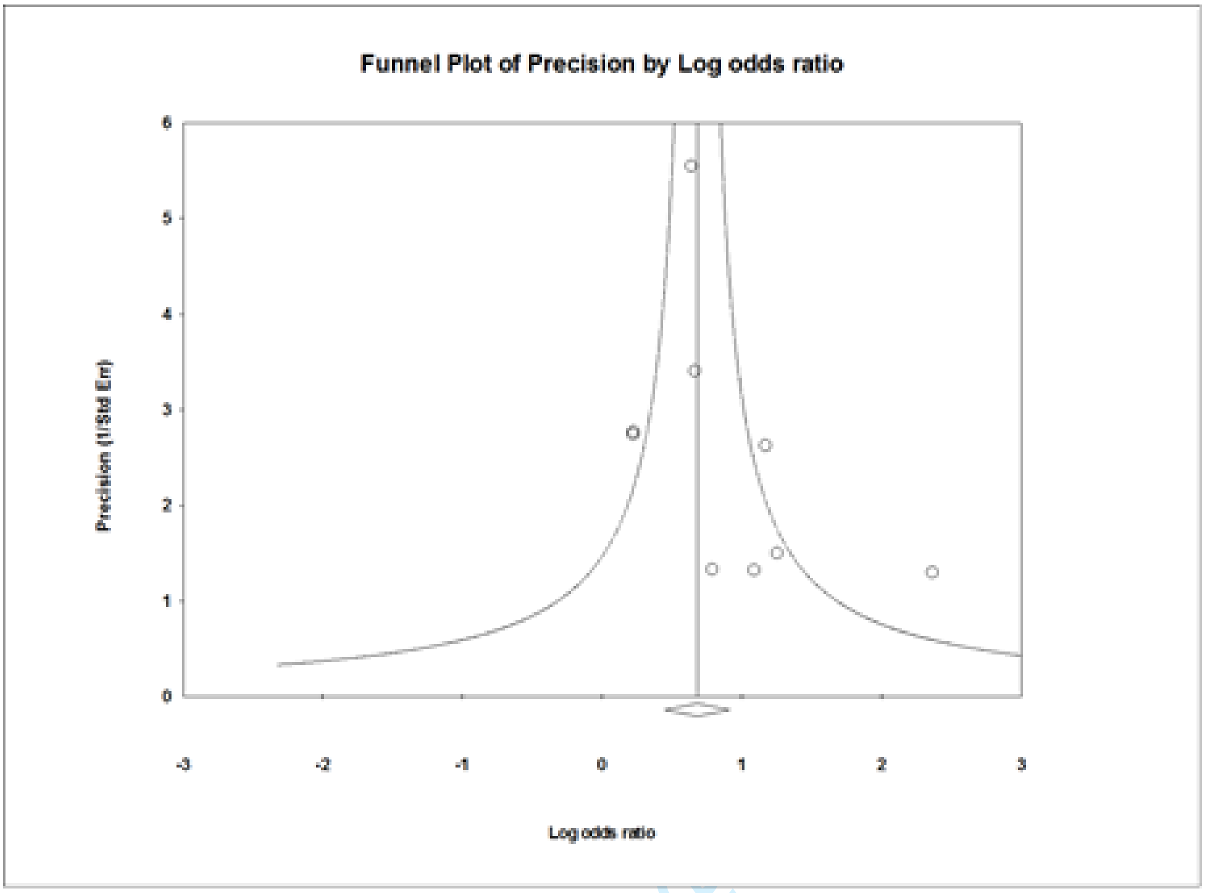

eTable 1: Relative weighting of each study included in the 9 study meta-analysis

Study	Weight (random)	Relative weight
Hammonds et al, 2015	2.16	3.79
Labovitz et al, 2017	1.63	2.86
Lakshminarayana et al, 2017	9.76	17.10
Mertens et al, 2018	1.70	2.97
Mira et al, 2014	6.69	11.72
Morawski et al, 2018	20.58	36.03
Santo et al, 2018	6.68	11.69
Shah et al, 2016	1.72	3.01
Svendsen et al, 2018	6.19	10.84

For peer review only

eTable 2: Raw data extracted for each study for meta-analysis

Study	Adherence measurement	Intervention group				Comparator group			
		Mean (SD)	N	Events	Totals	Mean (SD)	N	Events	Totals
Hammonds et al, 2015	Number of participants taken ≥80% prescribed tablets per day	-	-	12	20	-	-	6	20
Labovitz et al, 2017	Mean cumulative adherence, measured by pill count	97.2 (4.4)	15	-	-	90.6 (5.8)	12	-	-
Lakshminaray-ana et al, 2017	MMAS-8	6.3 (1.52)	68	-	-	5.74 (1.53)	90	-	-
Mertens et al, 2018	A14 Scale	53.96 (2.01)	12	-	-	52.6 (2.49)	12	-	-
Mira et al, 2014	MMAS-4	7.4 (0.9)	51	-	-	7.3 (0.7)	48	-	-
Morawski et al, 2018	MMAS-8	6.3 (1.6)	209	-	-	5.7 (1.8)	202	-	-
Santo et al, 2018	MMAS-8	7.02 (n/a)	50	-	-	6.63 (n/a)	51	-	-
Shah et al, 2016	MMAS-8	2.2 (1.2)	5	-	-	2.1 (1)	3	-	-
Svendsen et al, 2018	Number of patients having applied medication ≥80% days in treatment period	-	-	39	59	-	-	23	61

A14 = A14 Medication Adherence Scale; MMAS-8 = 8 Item Morisky Medication Adherence Scale; MMAS 4 = 4 Item Morisky Medication Adherence Scale

For peer review only

For peer review only - http://bmjopen.bmj.com/site/about/guidelines.xhtml

eTable 3: Behaviour Change Techniques utilised by in apps in the included studies.

Study	BCTs Coded							
	Tailored	Prompts and cues	Report whether behaviour was performed	Monitoring of behaviour by others without feedback	Feedback on behaviour	Social support (unspecified)	Habit formation	Goal setting of behaviour
Hammonds et al, 2015	+	+	+	-	-	-	-	-
Labovitz et al, 2017	+	+	+	+	+	-	-	-
Lakshminarayana et al, 2017	+	+	+	+	+	-	-	-
Mertens et al, 2016	+	+	+	-	-	-	-	-
Mira et al, 2014	+	+	+	+	+	-	+	+
Morawski et al, 2018	+	-	+	-	+	+	+	+
Santo et al, 2018	+	-	+	-	+	+	+	-
Shah et al, 2016	+	-	-	-	-	+	-	-
Svendsen et al, 2018	+	-	-	-	+	-	-	-

Those Behaviour Change Techniques coded in 3 but not more than 6 studies were included for meta-regression.

For peer review only - http://bmjopen.bmj.com/site/about/guidelines.xhtml

eTable 4: Definitions of Behaviour Change Techniques coded as present in apps of the included studies and examples of each.

Behaviour Change Technique	Definition	Examples
Tailored	Use information gained about the person to define the content, frequency or format of an intervention directed to the behaviour.	Shah et al, 2016: information about the participant including their symptoms and coronary artery catheterising procedure were recorded and used to tailor the information delivered to the participant via the MyIDEA application.[1]<br><br>Mira et al, 2014: The ALICE app delivered a customised system of alerts and reminders, according to their personalised prescription to remind participants when to take their medication.[2]
Prompts and Cues	"Introduce or define environmental or social stimulus with the purpose of prompting or cueing the behaviour. The prompt and cue would normally occur at the time or place of performance".[3]	Mira et al, 2014: The ALICE app delivered a customised system of alerts and reminders, according to their personalised prescription to remind participants when to take their medication.[2]<br><br>Lakshminarayana et al, 2017: The app included a reminder system for participants to set up and allow medication alerts.[4]
Report whether or not behaviour was performed	Ask a person to report when they have performed a behaviour without the requirement of monitoring or recording the behaviour.[5]	Santo et al, 2018: Reminders from the application could be snoozed, rescheduled and/or marked as 'taken' or 'missed dose'.[6]<br><br>Hammonds et al, 2015: Participants indicated when they had taken their medication by responding to the alert message.[7]

For peer review only

Monitoring of behaviour by others without feedback	"Observe or record behaviour with the person's knowledge as part of a behaviour change strategy".[3]	Labovitz et al, 2017: Clinic staff received automated text messages or emails if doses were missed, late or based on incorrect usage.[8]<br><br>Lakshminarayana et al, 2017: Clinicians had access to a dedicated web portal where they could see the participants' progress over the trial period.[9]
Feedback on behaviour	"Monitor and provide informative or evaluative feedback on performance of the behaviour (e.g. form, frequency, duration, intensity".[3]	Mira et al, 2014: The ALICE app reported to the participant medication that they had taken that day and those that the participant had not taken.[2]<br><br>Santo et al, 2018: The app included a feature of medication adherence statistics.[10]
Social support (unspecified)	"Advise on, arrange or provide social support (e.g. from friends, relatives, colleagues, buddies or staff) or non-contingent praise or reward for performance of the behaviour. It includes encouragement and counselling, but only when it is directed at the behaviour".[3]	Morawski et al, 2018: Users were able to designate a "Medfriend" who was granted access to the person's medication taking history and received alerts when doses were missed and could provide peer support.[11]<br><br>Santo et al, 2018: The app included the ability to export and share information with others such as family members and the ability to alert them if the participant missed a dose.[10]
Habit formation	"Prompt rehearsal and repetition of the behaviour in the same context repeatedly so that the context elicits the behaviour".[3]	Mira et al 2014: alerts and reminders encouraged participants to put healthy habits into practice, e.g. the association of taking their medicines with particular meals or daily activities.[2]<br><br>Santo et al, 2018: Scheduled reminders occurred up to three times at 10 minute intervals.[10]

For peer review only - http://bmjopen.bmj.com/site/about/guidelines.xhtml
For peer review only

Goal setting of behaviour	"Set or agree a goal defined in terms of the behaviour to be achieved".[3]	Mira et al, 2014: The ALICE app included a function for the participant to store details of their prescriptions and related instructions.[2]<br><br>Morawski et al, 2018: Users were able to enter their medication lists manually, along with their preferred timing of administration.[11]

References

1 Shah V, Dileep A, Dickens C, *et al.* Patient-Centered Tablet Application for Improving Medication Adherence after a Drug-Eluting Stent. *Front public Heal* 2016;**4**:272. doi:10.3389/fpubh.2016.00272

2 Mira JJ, Navarro I, Botella F, *et al.* A Spanish pillbox app for elderly patients taking multiple medications: randomized controlled trial. *J Med Internet Res* 2014;**16**:e99. doi:10.2196/jmir.3269

3 Michie S, Richardson M, Johnston M, *et al.* The Behavior Change Technique Taxonomy (v1) of 93 Hierarchically Clustered Techniques: Building an International Consensus for the Reporting of Behavior Change Interventions. *Ann Behav Med* 2013;**46**:81–95. doi:10.1007/s12160-013-9486-6

4 Lakshminarayana R, Wang D, Burn D, *et al.* Using a smartphone-based self-management platform to support medication adherence and clinical consultation in Parkinson ' s disease. *NPJ Park Dis* 2017;**3**:1–8. doi:10.1038/s41531-016-0003-z

5 Kassavou A, Sutton S. Automated telecommunication interventions to promote adherence to cardio-metabolic medications: meta-analysis of effectiveness and meta-regression of behaviour change techniques. *Health Psychol Rev* 2018;**12**:25–42. doi:10.1080/17437199.2017.1365617

6 Santo K, Singleton A, Rogers K, *et al.* Medication reminder applications to improve adherence in coronary heart disease: a randomised clinical trial. *Heart* 2018;:heartjnl-2018-313479. doi:10.1136/heartjnl-2018-313479

7 Hammonds T, Rickert K, Goldstein C, *et al.* Adherence to Antidepressant Medications: A Randomized Controlled Trial of Medication Reminding in

For peer review only - http://bmjopen.bmj.com/site/about/guidelines.xhtml

College Students. 2015;**8481**. doi:10.1080/07448481.2014.975716

8 Labovitz DL, Shafner L, Gil MR, *et al.* Using Artificial Intelligence to Reduce the Risk of Nonadherence in Patients on Anticoagulation Therapy. *Stroke* 2017;**48**:1416–9. doi:10.1161/STROKEAHA.116.016281

9 Lakshminarayana R, Wang D, Burn D, *et al.* and adherence tools to manage Parkinson ' s disease (SMART-PD): study protocol for a randomised controlled trial (v7 ; 15 August 2014). 2014;:1–11.

10 Santo K, Chow CK, Thiagalingam A, *et al.* MEDication reminder APPs to improve medication adherence in Coronary Heart Disease (MedApp-CHD) Study : a randomised controlled trial protocol. 2017;:1–10. doi:10.1136/bmjopen-2017-017540

11 Morawski K, Ghazinouri R, Krumme A, *et al.* Association of a Smartphone Application With Medication Adherence and Blood Pressure Control. *JAMA Intern Med* 2018;**178**:802. doi:10.1001/jamainternmed.2018.0447

For peer review only

PRISMA 2009 Checklist

Section/topic	#	Checklist item	Reported on page #
**TITLE**			
Title	1	Identify the report as a systematic review, meta-analysis, or both.	1
**ABSTRACT**			
Structured summary	2	Provide a structured summary including, as applicable: background; objectives; data sources; study eligibility criteria, participants, and interventions; study appraisal and synthesis methods; results; limitations; conclusions and implications of key findings; systematic review registration number.	2-3
**INTRODUCTION**			
Rationale	3	Describe the rationale for the review in the context of what is already known.	4-5
Objectives	4	Provide an explicit statement of questions being addressed with reference to participants, interventions, comparisons, outcomes, and study design (PICOS).	6
**METHODS**			
Protocol and registration	5	Indicate if a review protocol exists, if and where it can be accessed (e.g., Web address), and, if available, provide registration information including registration number.	5
Eligibility criteria	6	Specify study characteristics (e.g., PICOS, length of follow-up) and report characteristics (e.g., years considered, language, publication status) used as criteria for eligibility, giving rationale.	6
Information sources	7	Describe all information sources (e.g., databases with dates of coverage, contact with study authors to identify additional studies) in the search and date last searched.	5
Search	8	Present full electronic search strategy for at least one database, including any limits used, such that it could be repeated.	Supplementary file p 2
Study selection	9	State the process for selecting studies (i.e., screening, eligibility, included in systematic review, and, if applicable, included in the meta-analysis).	6
Data collection process	10	Describe method of data extraction from reports (e.g., piloted forms, independently, in duplicate) and any processes for obtaining and confirming data from investigators.	6-7
Data items	11	List and define all variables for which data were sought (e.g., PICOS, funding sources) and any assumptions and simplifications made.	6-8 and Table 1, p21
Risk of bias in individual studies	12	Describe methods used for assessing risk of bias of individual studies (including specification of whether this was done at the study or outcome level), and how this information is to be used in any data synthesis.	7
Summary measures	13	State the principal summary measures (e.g., risk ratio, difference in means).	7
Synthesis of results	14	Describe the methods of handling data and combining results of studies, if done, including measures of consistency (e.g., I^2) for each meta-analysis.	7-8

For peer review only

PRISMA 2009 Checklist

Section/topic	#	Checklist item	Reported on page #
Risk of bias across studies	15	Specify any assessment of risk of bias that may affect the cumulative evidence (e.g., publication bias, selective reporting within studies).	7
Additional analyses	16	Describe methods of additional analyses (e.g., sensitivity or subgroup analyses, meta-regression), if done, indicating which were pre-specified.	7
**RESULTS**			
Study selection	17	Give numbers of studies screened, assessed for eligibility, and included in the review, with reasons for exclusions at each stage, ideally with a flow diagram.	8
Study characteristics	18	For each study, present characteristics for which data were extracted (e.g., study size, PICOS, follow-up period) and provide the citations.	P8-9 and Table 1, p 21
Risk of bias within studies	19	Present data on risk of bias of each study and, if available, any outcome level assessment (see item 12).	P10 and Table 2, p 23
Results of individual studies	20	For all outcomes considered (benefits or harms), present, for each study: (a) simple summary data for each intervention group (b) effect estimates and confidence intervals, ideally with a forest plot.	Figure 2,<br>(and eTable 1 for weighting of each study and eTable 2 for raw extracted data found in supplementary file, p 4-5)
Synthesis of results	21	Present results of each meta-analysis done, including confidence intervals and measures of consistency.	9
Risk of bias across studies	22	Present results of any assessment of risk of bias across studies (see Item 15).	11 and efigure 1 on p 3 of supplementary material file
Additional analysis	23	Give results of additional analyses, if done (e.g., sensitivity or subgroup analyses, meta-regression [see Item 16]).	11
**DISCUSSION**			

For peer review only

PRISMA 2009 Checklist

Summary of evidence	24	Summarize the main findings including the strength of evidence for each main outcome; consider their relevance to key groups (e.g., healthcare providers, users, and policy makers).	11
Limitations	25	Discuss limitations at study and outcome level (e.g., risk of bias), and at review-level (e.g., incomplete retrieval of identified research, reporting bias).	13
Conclusions	26	Provide a general interpretation of the results in the context of other evidence, and implications for future research.	14
**FUNDING**			
Funding	27	Describe sources of funding for the systematic review and other support (e.g., supply of data); role of funders for the systematic review.	19

From: Moher D, Liberati A, Tetzlaff J, Altman DG, The PRISMA Group (2009). Preferred Reporting Items for Systematic Reviews and Meta-Analyses: The PRISMA Statement. PLoS Med 6(7): e1000097. doi:10.1371/journal.pmed1000097

For more information, visit: **www.prisma-statement.org**.

Page 2 of 2

For peer review only
